# Phylogenomic analysis of *Wolbachia* genomes from the Darwin Tree of Life biodiversity genomics project

**DOI:** 10.1371/journal.pbio.3001972

**Published:** 2023-01-23

**Authors:** Emmelien Vancaester, Mark Blaxter

**Affiliations:** Tree of Life, Wellcome Sanger Institute, Hinxton, United Kingdom; Instituto Gulbenkian de Ciencia, PORTUGAL

## Abstract

The Darwin Tree of Life (DToL) project aims to sequence all described terrestrial and aquatic eukaryotic species found in Britain and Ireland. Reference genome sequences are generated from single individuals for each target species. In addition to the target genome, sequenced samples often contain genetic material from microbiomes, endosymbionts, parasites, and other cobionts. *Wolbachia* endosymbiotic bacteria are found in a diversity of terrestrial arthropods and nematodes, with supergroups A and B the most common in insects. We identified and assembled 110 complete *Wolbachia* genomes from 93 host species spanning 92 families by filtering data from 368 insect species generated by the DToL project. From 15 infected species, we assembled more than one *Wolbachia* genome, including cases where individuals carried simultaneous supergroup A and B infections. Different insect orders had distinct patterns of infection, with Lepidopteran hosts mostly infected with supergroup B, while infections in Diptera and Hymenoptera were dominated by A-type *Wolbachia*. Other than these large-scale order-level associations, host and *Wolbachia* phylogenies revealed no (or very limited) cophylogeny. This points to the occurrence of frequent host switching events, including between insect orders, in the evolutionary history of the *Wolbachia* pandemic. While supergroup A and B genomes had distinct GC% and GC skew, and B genomes had a larger core gene set and tended to be longer, it was the abundance of copies of bacteriophage WO who was a strong determinant of *Wolbachia* genome size. Mining raw genome data generated for reference genome assemblies is a robust way of identifying and analysing cobiont genomes and giving greater ecological context for their hosts.

## Abstract 2

Wolbachia are common bacterial endosymbionts that manipulate reproductive biology in their hosts. This study assembles the genomes of 110 Wolbachia coincidentally sequenced alongside their insect hosts, finding a rich diversity of host manipulation loci.

## Introduction

The natural world is a complex web of interactions between living species. These interactions can be mutualistic, commensal, pathogenic, parasitic, predatory, or inconsequential, but each individual lives alongside a rich diversity of cobionts. Most eukaryotes associate intimately with a specific microbiota and are commonly infected by a range of microbial and other pathogens. For some microbial associates, the distinction between mutualism and pathogenicity or parasitism is fuzzy. For example, *Wolbachia* (Proteobacteria; Alphaproteobacteria; Rickettsiales; Anaplasmataceae; Wolbachieae) are found living intracellularly in a range of terrestrial arthropods and nematodes. No free-living *Wolbachia* are known: The association is essential for their survival. In contrast, infection with *Wolbachia* can be beneficial to hosts but is not usually essential.

*Wolbachia* were first identified as mosquito endobacteria that were maternally transmitted, through the oocyte, and that induced a range of reproductive manipulations on their hosts [1,2]. The most common manipulation by *Wolbachia* is to induce cytoplasmic incompatibility (CI). Under CI, infected females are able to mate productively with all males, but uninfected females are only able to mate with uninfected males (as mating with CI-inducing *Wolbacha*-infected males results in zygotic death). This asymmetry in fitness can drive spread of the CI-inducing *Wolbachia*. Other reproductive manipulations include feminisation of genetic males [3], male killing [4], and induction of parthenogenesis in females [5]. All these manipulations promote the transmission of infected oocytes to the next host generation and thus boost the spread of *Wolbachia*. In most species that can be infected, populations are a mix of infected and infection-free individuals, and hosts can evolve to resist infection [6,7]. While *Wolbachia* are often described as reproductive parasites, association with *Wolbachia* can sometimes have beneficial effects, providing nutritional supplementation to phloem-feeding Hemiptera [8] and enhancing host immunity to viruses and *Plasmodium* parasites [9]. Indeed, the host immunity-boosting phenotype may explain the initial spread of *Wolbachia* in previously uninfected populations. In nematodes, elimination of *Wolbachia* induces host sterility, and antibiotic treatment is an effective addition to pharmacological treatment of human-infecting, *Wolbachia*-positive filarial nematodes [10].

*Wolbachia* infection of terrestrial arthropods is very common, with nearly half of all insect species predicted to be infected [11]. *Wolbachia* can be classified using molecular phylogenetic analyses into a series of supergroups [12,13]. Supergroups C, D, and J are found only in filarial nematodes; supergroups E and F are found in both nematodes and insects; and supergroups A, B, and S (and others for which full genome data are not available) are found only in arthropods. Supergroups A and B are the most common *Wolbachia* found in terrestrial insects.

Analysis of *Wolbachia* biology has been expanded by the determination of genome sequences for many isolates. The genome sequences for *Wolbachia* from over 90 host species are publicly available, and mining of host genomic raw sequence data identified a large number of additional partial genomes [14,15]. This understanding, that cobiont genomes can be assembled from the “contamination” present in the data generated for a target host, has been especially useful for the unculturable *Wolbachia*. We now have the opportunity to survey for the presence of *Wolbachia* genomes at an unprecedented scale, as the Darwin Tree of Life (DToL) project aims to sequence all described terrestrial and aquatic eukaryotic species found in Britain and Ireland [16]. This project is using high-accuracy long read and chromatin conformation long range sequencing to generate and release publicly available chromosomal genome assemblies, meeting exact standards of contiguity and completeness, for thousands of protists, fungi, plants, and animals. Several hundred terrestrial arthropod assemblies are already available (https://portal.darwintreeoflife.org). The DToL project sequences genomes from individual, wild-caught specimens of target species, and thus will also generate data for the cobiome present in each specimen at the time of sampling. For many smaller-bodied insects, the whole organism is extracted. Where *Wolbachia* disseminates widely within an organism, it is inevitable that cobiont genomes will be sequenced alongside the host genome.

Using k-mer classification tools, it is possible to efficiently and correctly separate out cobiont data from that of the host and to deliver clean host assemblies [17–19]. The cobiont data are then available for independent assembly and analysis. Here, we present a survey of the first 368 terrestrial arthropod genome datasets produced in DToL for the presence of *Wolbachia* and assemble over 100 new *Wolbachia* genomes. We use these to explore patterns and processes in bacterial genome evolution and coevolution of *Wolbachia* with its hosts and with its own bacteriophage parasites. Lepidopteran hosts were mostly infected with supergroup B, while infections in Diptera and Hymenoptera were mainly caused by A-type *Wolbachia*. However, host and *Wolbachia* phylogenies revealed no (or very limited) cophylogeny. We show that while B genomes tended to be longer compared to supergroup A, genome size in *Wolbachia* is correlated with the level of integration of its double-stranded bacteriophage WO.

## Results

### Screening a diverse set of insect genome data for *Wolbachia* infections

**Fig 1:**
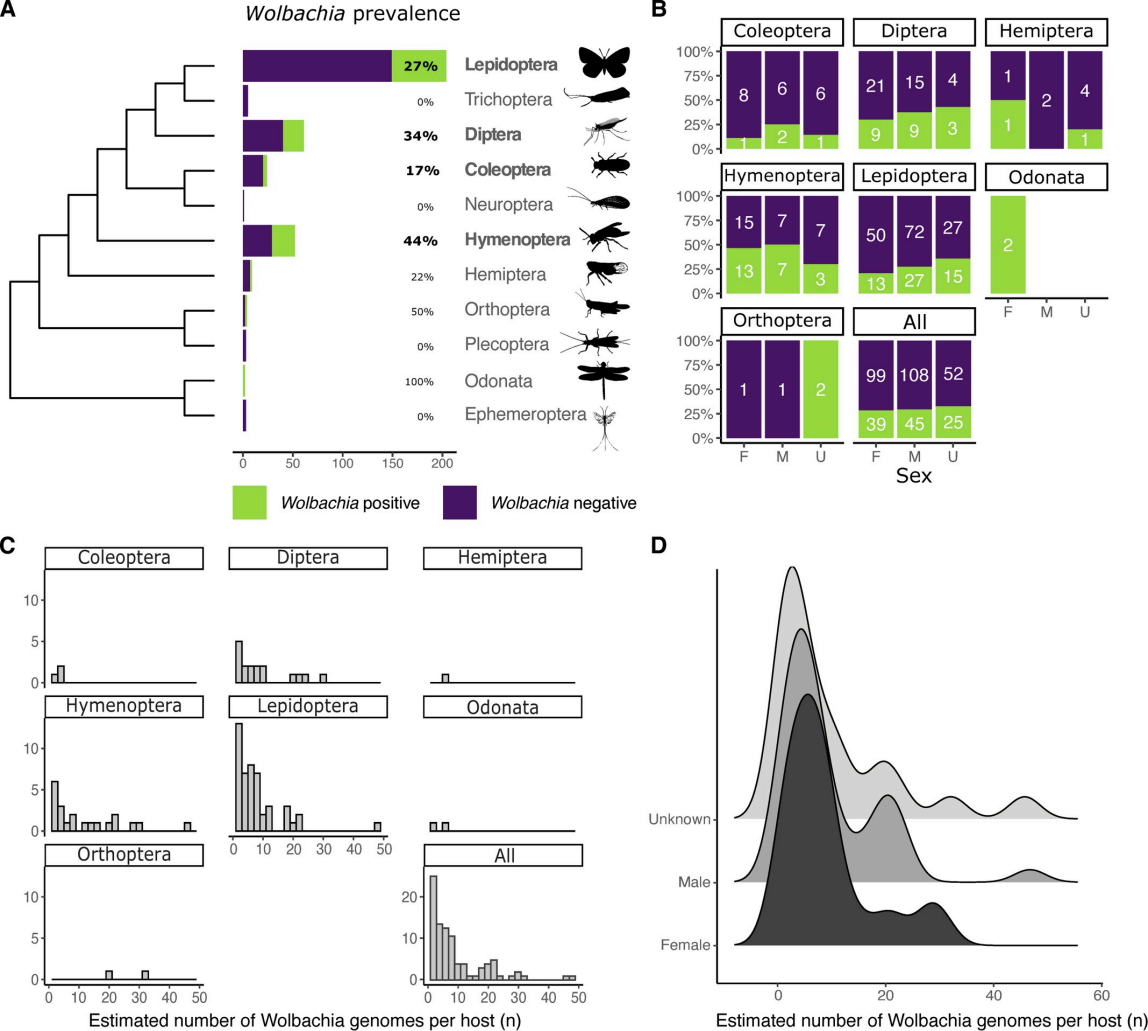
Prevalence and relative abundance of *Wolbachia* in DToL insect genomes. **(A, B)** Prevalence of *Wolbachia* in insect hosts, split by taxonomic order (**A**) and by sex (**B**). The cladogram of insect ordinal relationships is based on Misof and colleagues [28]. Orders with more than 10 analysed species are shown in bold. Silhouettes are from PhyloPic (http://phylopic.org/). Sex of insects was classified as F (female), M (male), or U (unknown, where not recorded on collection). The data underlying this Figure can be found in S1 Data. **(C, D)** The estimated number of *Wolbachia* genomes per copy of the host nuclear genome split by taxonomic order (**C**) and by sex (**D**). The data underlying this Figure can be found in S1 Data.

We screened raw genomic sequence data and primary assemblies for 368 insect species (204 Lepidoptera, 61 Diptera, 52 Hymenoptera, 24 Coleoptera, 9 Hemiptera, 5 Trichoptera, 4 Orthoptera, 3 Ephemeroptera, 3 Plecoptera, 2 Odonata, and 1 Neuroptera) generated by DToL for the presence of *Wolbachia* (S1 Table) using the small subunit ribosomal RNA (SSU rRNA) as a marker gene. *Wolbachia* SSU sequences were detected in 111 (30%) of the species. This level of infection is not reflective of total incidence, the proportion of host species susceptible to infection, as only one individual was analysed for each taxon screened. *Wolbachia* prevalence, the proportion of infected individuals in a population, and infection intensity vary between species and between populations within a species [20,21]. Therefore, the true incidence of infection within the insect biota surveyed by DToL is likely much higher. However, the measured incidence of infection is similar to previous survey-based estimates (approximately 22%) [22,23] but, as expected, is lower than estimates deploying mathematical models to account for sampling bias (40% to 50%) [11,24]. Infection incidence was lower in Coleoptera (4/24, 17%) compared to Lepidoptera (55/204, 27%), Diptera (21/61, 34%), and Hymenoptera (23/52, 44%) (Fig 1A).

Although maternal inheritance requires that *Wolbachia* are predominantly localised in the germline, tropism to somatic cell types has been shown to be highly regulated during host development [25,26]. We did not observe a bias in infection level by analysed tissue type (S1 Fig), or by gender, with an equal prevalence of infection in samples identified as female (39/138, 28%) and male (45/153, 29%) (Fig 1B). While the DToL project aims to sequence eukaryotes from across Britain and Ireland, 82% of the samples screened were sampled from the Wytham Woods Ecological Observatory, Oxfordshire (https://www.wythamwoods.ox.ac.uk/) [27]. No correlation between sampling location and infection level was detected, with 29% of all samples collected in Wytham Woods being *Wolbachia* positive, reflective of the overall incidence level (S2 Fig).

The DToL species were sequenced using PacBio Sequel II HiFi highly accurate long read platform, generating consensus raw reads of 10 to 20 kb with base level accuracy of >99% (approximately Q30 to Q40). These long, accurate reads are ideal for assembly, particularly for bacterial genomes where the information content per base is higher than in repeat-rich eukaryotes. The average sequence length of HiFi reads identified as being derived from *Wolbachia* was 12 kb, indistinguishable from host HiFi reads. We separated and assembled all *Wolbachia* reads in each positive sample and screened these assemblies to identify complete genomes. We generated 110 complete genomes, from 93 species, of which 77 were circular (S2 Table). The average completeness of these genomes, assessed using BUSCO, was 99.3%, with a mean duplication level of 0.37%. The mean genome size of the new genomes was 1.47 Mb, which is significantly larger than the average genome size of public *Wolbachia* genomes (1.32 Mb; Wilcoxon rank sum test, *p*-value = 4.576 × 10^−9^) (S3 Fig). This is likely because it is possible to assemble across repeated loci (such as integrated *Wolbachia* phage) with the long, accurate HiFi reads. The mean number of contigs generated for the 33 genomes that could not be circularised was 2.12 (ranging from 1 to 6).

The dataset includes the first complete circular *Wolbachia* genomes assembled from two insect orders, Odonata (dragonflies and damselflies) and Orthoptera (grasshoppers and crickets). Both species of dragonfly surveyed (Odonata) harboured *Wolbachia* (Fig 1A). The largest circular *Wolbachia* genome generated, 2.19 Mb, was isolated from the blue-tailed damselfly. This is the longest complete *Wolbachia* genome yet reported (S3 Fig). Although in most samples infection by only a single *Wolbachia* strain was detected, 15 of 93 specimens (16%) were infected with at least two *Wolbachia* genomes. Within *Phalera bucephala* (Lepidoptera) and *Lasioglossum morio* (Hymenoptera), three genomes were assembled, while all other coinfections involved two strains.

Having chromosomally complete insect host genomes, as well as complete *Wolbachia*, allows for the estimation of the relative numbers of *Wolbachia* genomes per host genome. *Wolbachia* proliferation seems to be tightly controlled and a relative abundance below ten *Wolbachia* genomes per host nuclear genome was observed in most infected hosts. Particularly high abundances were observed in *Thymelicus sylvestris* and *Athalia cordata* (48 and 47 *Wolbachia* per host genome, respectively) (S2 Table) (Fig 1C). The mean relative abundance in different taxonomic orders lay between 3 and 12, except for the two crickets (Orthoptera), *Chorthippus brunneus* and *Chorthippus parallelus*, which have a 33 and 20 *Wolbachia* genome copies per host genome, respectively (Fig 1C). No significant difference was observed between relative *Wolbachia* abundance and sex of the host (Fig 1D), with both male and female having a mean between nine and ten copies.

### *Wolbachia* phylogeny suggests frequent host switching events

**Fig 2:**
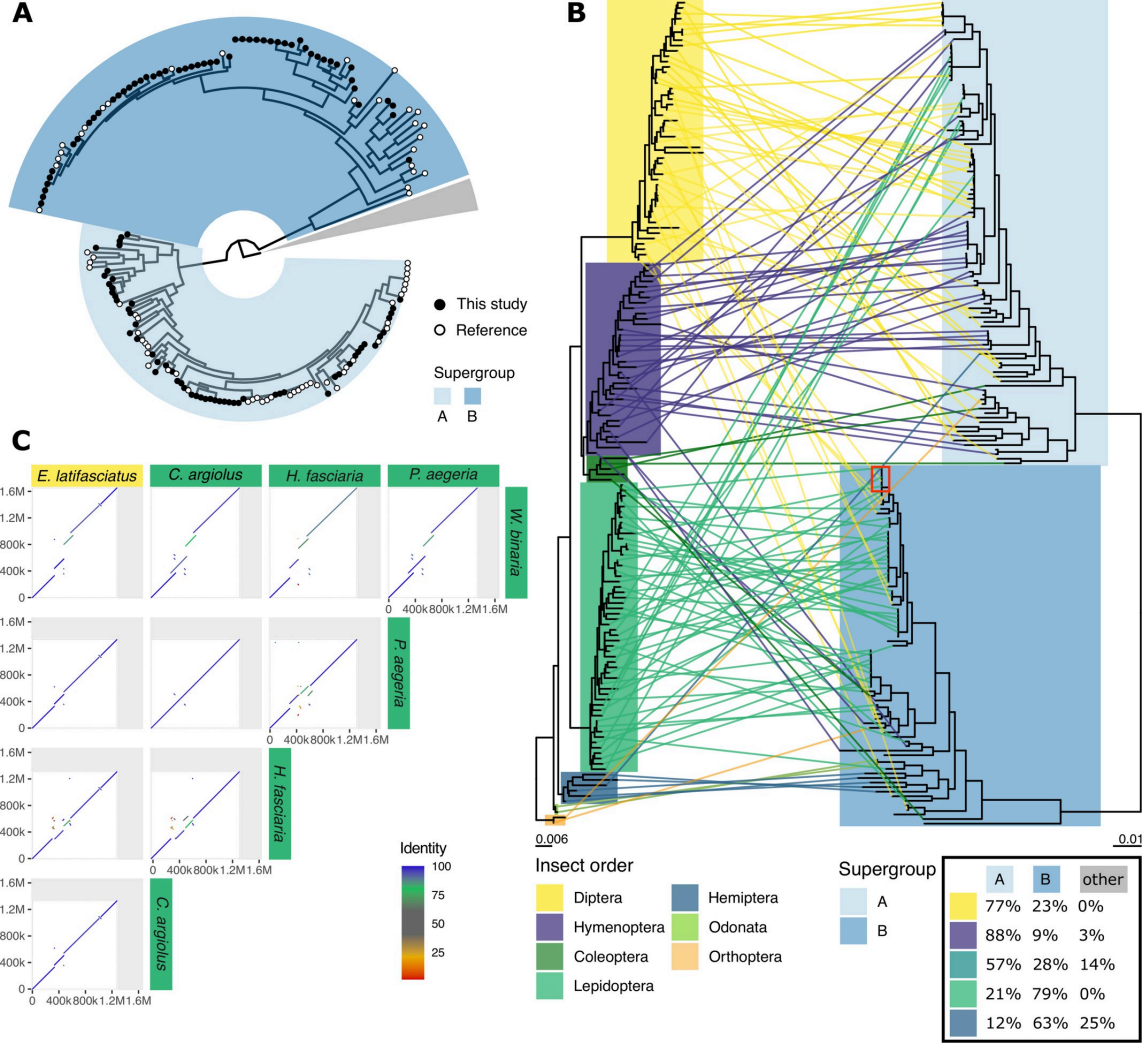
*Wolbachia* DToL genomes expand known phylogeny. **(A)** Circular phylogeny of supergroup A and B *Wolbachia*, visualised with the root placed between the A and B supergroups and the remaining supergroups (C, D, E, F, J, S; nodes collapsed as grey wedge), highlighting newly sequenced genomes (black tip labels) and genomes from public databases (white). (**B)** Incongruence between host topology (left) and supergroup A and B *Wolbachia* topology (right) is shown as a tanglegram. Overview of the supergroups infecting diverse insect orders is given in a table (inset, bottom right). A red box is drawn to point to a host switching event; see panel C. **(C)** Example of a host switching event, where the *Wolbachia* of the hoverfly *Eupeodes latifasciatus* has high nuclear sequence identity and genome colinearity to four *Wolbachia* genomes assembled from Lepidoptera.

We selected 93 high-contiguity and high-completeness *Wolbachia* genomes from the public INSDC databases, including genomes from *Wolbachia* infecting Nematoda (13 genomes), Arachnida (4), Isopoda (1), and several orders of Hexapoda (75) (S3 Table). Adding the 110 newly assembled genomes yielded a dataset of over 200 high-quality assemblies. We annotated all protein-coding genes in those genomes using Prodigal [29] and clustered the predicted protein sets into orthologous groups using OrthoFinder2 [30]. The resulting 634 near-single copy genes were used to infer a phylogeny of *Wolbachia* (Figs 2A and S4). From this phylogeny, we assigned each genome to the previously defined *Wolbachia* supergroups [12,13]. All newly assembled *Wolbachia* genomes belonged to either supergroup A or B. While Lepidoptera were predominantly infected with supergroup B *Wolbachia* (42/53, 80%), *Wolbachia* supergroup A was most frequent in all other insect classes (46/57, 81%). It has been previously observed that supergroup B is the most common *Wolbachia* type in Lepidoptera [22,31–33]. Of the 15 species where coinfections occurred, *Endotricha flammealis*, *Phalera bucephala*, *Philonthus cognatus*, *Protocalliphora azurea*, and *Sphaerophoria taeniata* were coinfected with strains from both A and B supergroups, and the other ten coinfections were of distinct strains within the same supergroup (S2 Table).

*Wolbachia* generally do not show strict cophylogeny with their hosts [7,21]. This pattern was also observed when comparing host and *Wolbachia* phylogenies for the supergroup A and B genomes (Fig 2B). Closely related insect species may be infected by dissimilar *Wolbachia* strains, and, conversely, closely related *Wolbachia* can infect a diverse set of insects. For example, the *Wolbachia* strains infecting the hoverfly *Eupeodes latifasciatus* and four Lepidoptera (*Pararge aegeria*, *Celastrina argiolus*, *Hylaea fasciara*, and *Watsonella binaria*) (Fig 2C) share over 99% nucleotide identity. Although horizontal transmission seems to have been a dominant pattern in the evolutionary history of *Wolbachia*, the propensity of Lepidoptera to be infected by *Wolbachia* type B underlines the importance of distribution by cospeciation. Because most of our new samples came from a single site (Wytham Woods Genomic Observatory), we were also able to explore the horizontal transfer of *Wolbachia* between hosts in a local context. Wytham Woods–derived *Wolbachia* were no more likely to be related than any other *Wolbachia* subset (S5 Fig).

### Intrinsic properties of *Wolbachia* distinguish supergroups

**Fig 3:**
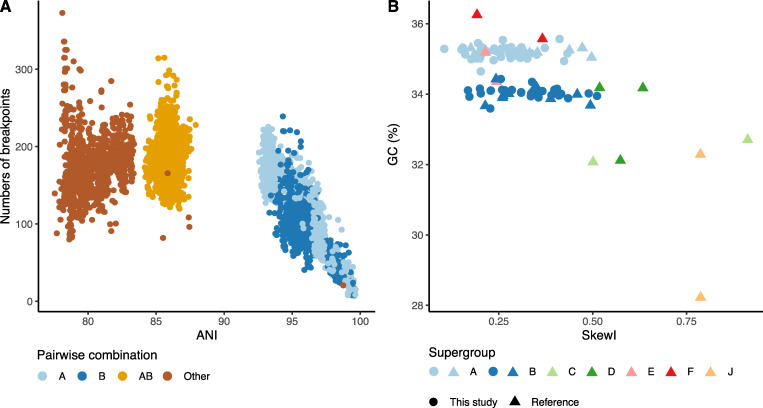
Comparative genomics of *Wolbachia*. **(A)** Whole-genome average nucleotide identity (ANI) plotted against the number of breakpoints in comparisons within A supergroup genomes, within B, between A and B and between other supergroup *Wolbachia*. The data underlying this Figure can be found in S1 Data. **(B)** Index of skewness compared to GC content for all circularised *Wolbachia* genomes. The data underlying this Figure can be found in S1 Data.

The completeness of the new genomes and, in particular, the circular assemblies achieved for 77 of them permits analyses of genome properties that are not possible with fragmented and partial genomes. All circularised genomes, including those from public databases, were rotated to start at the presumed origin of replication. The average pairwise whole-genome nucleotide identity between all *Wolbachia* genomes ranged between 77.3% and 100.0%, with at least 92.8% and 93.5% identity within supergroups A and B, respectively (Fig 3A). The number of breakpoints interrupting pairwise whole-genome alignments was counted, normalised for the total alignable length, and compared to average nucleotide identity (ANI) of the compared genomes (Fig 3A). A significant correlation was observed between nucleotide divergence and the number of breakpoints in supergroups A (0.90, *p* < 2.2 × 10^−16^, Spearman correlation) and B (0.69, *p* < 2.2 × 10^−16^, Spearman correlation) (Fig 3A). This broad range of nucleotide diversity, even within a supergroup, is indicative of the low level of conserved synteny within supergroups and the level of rearrangements occurring.

Stable bacterial genomes accumulate more guanines than cytosines on the strand in the direction of replication. This phenomenon, GC skew, arises due to differential mutation pressures on leading versus lagging strands. Genomes that have undergone frequent rearrangement are expected to have lower overall GC skew, which can be summarised across the genome as a single metric, SkewI [34]. Genomes from supergroups A and B had distinct GC contents (Fig 3B), with supergroup A having a higher mean GC (35.2%, standard deviation 0.15%), compared to B (34.0%, standard deviation 0.16%) (two-sample *t* test *p*-value < 2.2 × 10^−16^). Genomes from other supergroups had distinct GC content, often very different from A and B genomes, but as so few examples have been sequenced, general patterns are not discernible. In both A and B supergroups, SkewI values were relatively low, but genomes from Wolbachia from nematode hosts (C, D, J) had higher SkewI values (Fig 3B). A high degree of GC skew was previously reported in supergroup C *Wolbachia* strains infecting filarial nematodes [35], and these genomes also have low rearrangement levels and high gene-level synteny. In supergroups A and B, the low level of skew is associated with high levels of chromosomal rearrangement (Fig 3A).

### Conservation and diversity in gene content of *Wolbachia*

*Wolbachia*, because they are sheltered within the cells of their hosts, may be relatively isolated from other bacteria and thus have somewhat closed pan-genomes. One route to acquisition and sharing of new genes is through the *Wolbachia* phage (WO phage), which alongside the essential phage particle structural genes carry a cargo of genes that have been implicated in host manipulation. We reannotated all 203 *Wolbachia* with the same, standard gene finding toolkit, Prodigal, to normalise annotations. While this may have lost careful manual revision in previously determined gene sets, it avoids issues of data incompatibility. Gene number correlated with genome size and the average gene number in the newly assembled set of supergroup A and B *Wolbachia* was larger than in A and B genomes from the public databases (S6 Fig). Comparing all genomes, the mean number of predicted genes was larger in supergroup B (1,467) compared to A (1,385).

**Fig 4:**
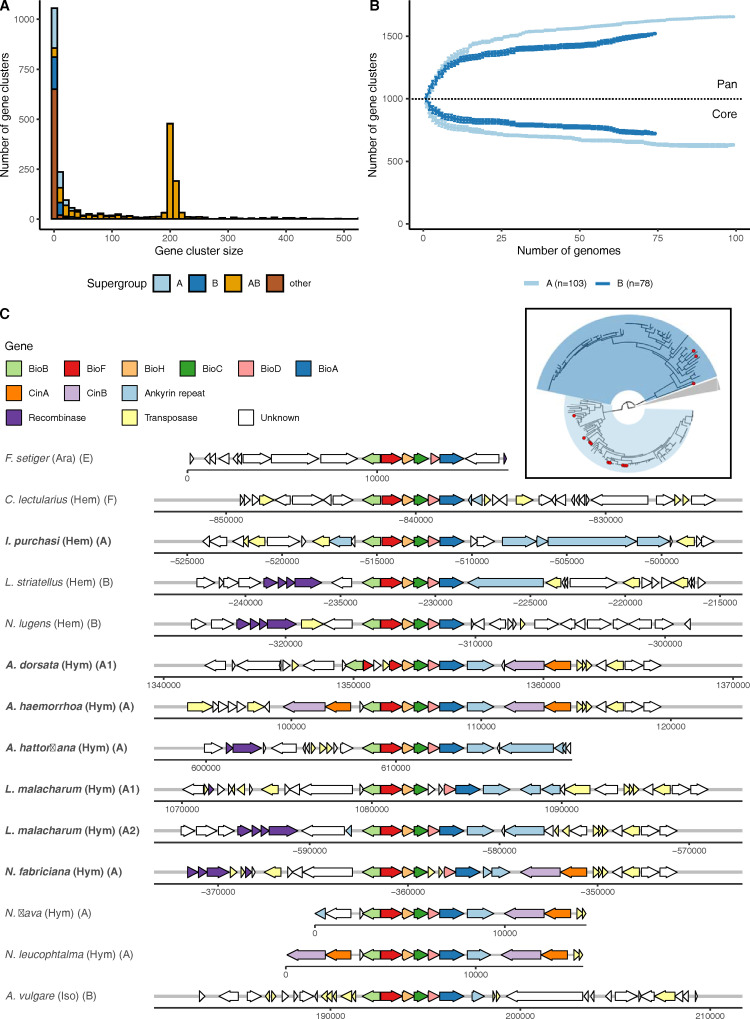
Exploration of *Wolbachia* protein-coding gene diversity. **(A)** Histogram of protein family size per supergroup. The data underlying this Figure can be found in S1 Data. **(B)** Rarefaction analysis of pan- and core proteomes of supergroups A and B, based on 500,000 random addition-order permutations of co-occurring orthogroups excluding novel genes. The data underlying this Figure can be found in S1 Data. **(C)** Synteny of the biotin cluster shows conserved gene order and punctuated pattern of species presence (inset, species with biotin cluster present are highlighted with red circles).

We used OrthoFinder with default settings to define clusters of orthologous proteins across all *Wolbachia* genomes. Each genome contained between 0 and 184 novel, strain-specific genes (average 19). These novel genes were shorter than all genes (average gene length overall was 875 nucleotides or approximately 290 amino acids, while novel genes averaged 434 nucleotides or approximately 145 amino acids). As expected, supergroups that were not well represented often contained more strain-specific genes. For example, wCfeT from supergroup E (which infects cat fleas, *Ctenocephalides felis*) uniquely encoded genes for pantothenate (panC-panG-panD-panB) [36] and thiamine (thiG-thiC) biosynthesis. Nonetheless, out of the ten genomes with most strain-specific genes, seven belonged to either supergroup A or B. These novel genes were not preferentially associated with WO phage regions (S7 Fig), but the majority (78%) had annotations that associated them with transposon and mobile element function. This suggests that much of the novelty is associated with mobile elements other than WO phage, but we note that the expansion in gene number may be due to mobile element-driven pseudogenisation. Other than clusters with one or two members, the most frequently observed cluster sizes were 203 ± 2. These clusters contained the single-copy (and near-single-copy) orthologs deployed in phylogenetic analyses (Fig 4A). Overall, the majority of the proteins encoded in the *Wolbachia* genomes were members of orthology clusters that were present in at least 95% of all strains.

The abundant sampling of supergroup A and B genomes allowed us to address and compare the sizes of the core- and pan-proteomes of these groups. The larger genome and proteome size found in supergroup B was reflected in a larger core proteome (Fig 4B), but supergroup A had a larger pan-proteome (Fig 4B). While the core proteomes differed, very few of the protein families that were part of each supergroup’s core proteome were unique to that supergroup. One supergroup-restricted set of protein families was found to comprise the operon for arginine transport (ArtM, ArtQ, and ArtP and the repressor of arginine degradation ArgR) [37], which was uniquely detected and conserved in supergroup A (present in 83/103 or 80% of all *Wolbachia* A genomes). Although the periplasmic arginine-specific binding protein (ArtI or ArtJ) was not detected, the presence of this ATP-binding cassette-type (ABC) transporter suggests that these *Wolbachia* are acquiring arginine from their hosts.

The operon-producing biotin (vitamin B7) [38] was detected in seven of the 110 new genomes, all belonging to supergroup A (Fig 4C). One derived from *Icerya purchasi* (Hemiptera), and six were from Hymenoptera (two from *Lasioglossus malacharum*, which carried two strains, and single strains from three *Andrena* and a *Nomada* species). The biotin synthesis cluster has been described previously from a restricted but diverse set of supergroups, including two A genomes from additional *Nomada* bee hosts. This distribution suggests possible ecological linkage [39], as *Andrena* bees are kleptoparasitised by *Nomada* cuckoo bees and phylogenetic analyses of both the biotin gene clusters and the *Wolbachia* core proteomes show close relationships between these clusters of genomes (S8 Fig). The gene cluster is strongly conserved in physical organisation of all six necessary genes (bioA-D,F,H). In the genomic region immediately surrounding the operon, we identified recombinase and transposase genes, as well as ankyrin repeat containing genes and toxin–antitoxin CI Cin gene pairs. In three genomes (from *Andrena dorsata*, *Nomada fabricium*, and one of the *L*. *malacharum* strains), the operon was independently disrupted by transposases. The region containing the biotin operon thus has the hallmarks of a “virulence island” that may be mobile between genomes and may have accrued additional genes (ankyrin, Cin) that hitchhike with the biotin operon.

### WO prophage insertions expand genome size

**Fig 5:**
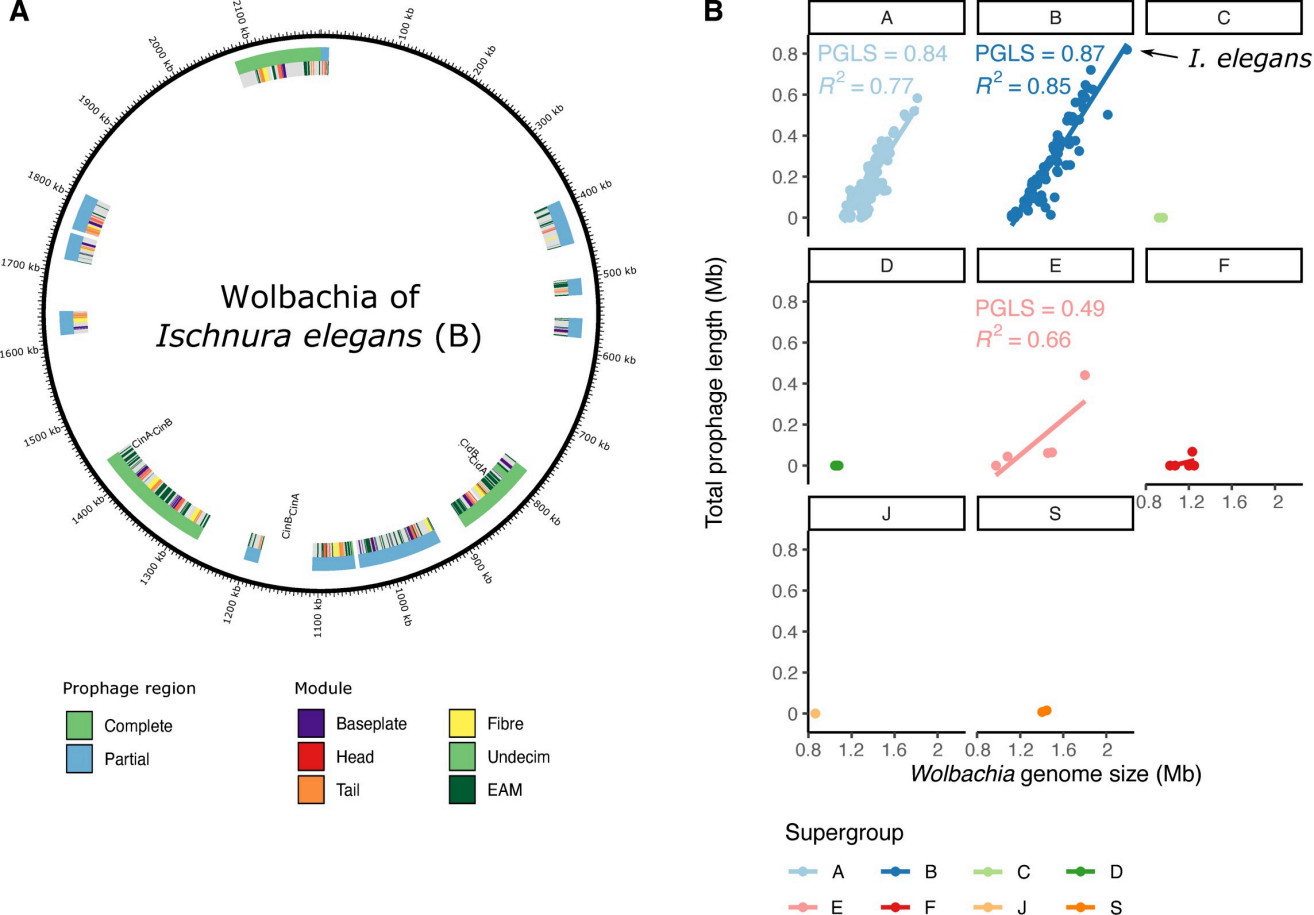
WO prophage in *Wolbachia*. **(A)** Annotation of the WO prophage integrated in the genome of the *Wolbachia* strain infecting *Ischnura elegans*. **(B)** *Wolbachia* genome size is strongly correlated with integrated prophage span in supergroups with WO phage association. Phylogenetic generalised least squares (PGLS) analyses were performed to assess the correlation between prophage length and genome size in a phylogenetically aware manner. The data underlying this Figure can be found in S1 Data.

*Wolbachia* can itself be infected by double-stranded DNA temperate bacteriophages, WO phage, which can integrate in the genome of its host as a prophage. Four modules are necessary for construction and function of phage particles during the lytic stage: head, baseplate, tail, and fibre, and inserted and pseudogenised *WO* phage can be identified and discriminated based on the presence and completeness of these components. Regions of a *Wolbachia* genome flanked by WO phage modules are likely to form components that are transduced by the phage during infection of new cells, “cargo” loci that form the eukaryotic association module (EAM) [40,41]. All the *Wolbachia* genomes were screened for prophage regions using essential module genes from previously annotated WO insertions (S4 Table). Prophage regions were deemed putatively complete when all four modules were observed with at least 80% of genes of each module present. An abundance of putative intact and pseudogenised WO phage were identified. For example, the supergroup B *Wolbachia* from *Ischneura elegans* (the bluetail damselfly; the largest *Wolbachia* genome assembled) contained three putative intact prophage and nine WO phage fragments (Fig 5A) summing to 0.8 Mb of the genome.

The fraction of total prophage region in each genome ranged from 0% to 38%. Nematode-associated *Wolbachia* typically are not infected by WO phage [42], and no prophage regions were detected in genomes of supergroups C, D, J, and nematode-infecting F (Fig 5B). A significant correlation was found between genome size and WO prophage span in supergroups A and B (Fig 5B). This association was robust to correction for phylogenetic relatedness of the genomes (model fit increased to 0.84 and 0.87, respectively, with *p*-values <10^−16^).

### Toxins are often associated with mobile elements

We identified several potential cargo genes within intact and fragmented prophage. These included transposases and integrases associated with mobile elements, and other loci previously associated with eukaryotic manipulation, such as CI loci and ankyrin repeat containing genes, as expected from the EAM model [40,41].

*Wolbachia* produces a suite of toxins [43] that can have dramatic effects on their hosts, such as CI. The CI phenotype is caused by two adjacent genes, CifA and CifB, which function as a toxin–antitoxin pair [44,45]. Phylogenetic analysis classified most *Wolbachia* Cif gene pairs into four types (I to IV) [46]. A fifth type (V) is much more variable in structure. The toxin component can have nuclease activity (in which case the gene pair is frequently referred to as CinA-CinB), deubiquitinase (CidA-CidB), or both (CndA,CndB) [47]. All type II, III, and IV pairs have nuclease domains, while all type I have deubiquitinase and most have nuclease [46]. Three hundred and five full-length and likely functional Cif pairs were detected in 140 of the 181 (77%) supergroup A and B genomes. One Cif pair was detected in most genomes, but many had several, with seven copies in the *Wolbachia* strain infecting the holly tortrix moth (*Rhopobota naevana*). Most of the gene pairs contained a deubiquitinase domain (type I, Cid) (87) or belonged to type V (90), while the other three types occurred in roughly equal proportions (II: 39, III: 44, IV: 34) (S9 and S10 Figs). Many pairs (213/305; 70%) were located in the predicted EAM of the prophage.

Loci encoding additional toxins such as RelE/RelB and latrotoxin were identified in multiple *Wolbachia* genomes, frequently in prophage regions (175/586 [30%], 130/256 [51%] genes, respectively) (summarised in S5 Table). The Tc pore-forming toxin complex, which consists of two genes TcA (S11 Fig) and TcB-C (S12 Fig), was detected in a limited number of A and B supergroup genomes and also showed a predisposition to occur within prophage (42/69 [61%] and 19/35 [54%], respectively). Additional toxin-encoding loci had limited presence in different subgroups and were not associated with prophage regions. ParD/ParE (S13 and S14 Figs) only occurred in supergroups A, B, and E, and FIC (S15 Fig) only occurred in supergroups A, E, F, and S. The type IV toxin–antitoxin gene pair AbiEii/AbiGii-AbiEi, which protects against the spread of phage infection [48], was only detected in two genomes in supergroup E. It is noteworthy that these two genomes had very low levels of prophage-derived DNA (4.3% of their genome span).

## Discussion

Isolation of cobiont genomes, and specifically *Wolbachia* genomes, from shotgun high-throughput sequencing data has been established for many years [49]. In the field of prokaryotic and eukaryotic microbial metagenomics, metagenome-assembled genomes (MAGs) are likely to be the only way to access many unculturable microbial genomes, even if the species they derive from are hyperabundant [50,51]. The abundance of raw sequencing data in the International Nucleotide Sequence Database Collaboration (INSDC) databases has been an attractive prospecting ground for microbial associates of eukaryotic target species. To date, most raw data available for such searches have been short reads from Illumina and other platforms. These reads are too short to partition efficiently into bins corresponding to putative distinct genomes. Preliminary assembly of such datasets is more likely to be able to separate cobionts from target genomes. These approaches have been applied to hunt for *Wolbachia* with a recent tour de force generating nearly 1,200 *Wolbachia* MAGs from publicly available data [14]. However, these MAGs suffer from the expected issues of low completeness (due to low effective coverage), fragmentation (due to coverage and sequence repeat issues), undetected contamination, and inability to distinguish coinfecting strains. Moreover, the biased nature of public data meant that these derived from only 37 different host species.

We generated 110 *Wolbachia* assemblies from 368 terrestrial arthropod HiFi datasets, and 77 of these were fully circular genome assemblies. The genomes were uniformly of high completeness (S3 Fig). Due to the high intrinsic base quality of HiFi reads (Q30 to Q40; from one error in 1,000 to one error in 10,000), we were able to distinguish insertions of *Wolbachia* DNA into the host genome from true components of the *Wolbachia* genome and to independently assemble even closely related strains with confidence. As we were screening raw data from a biodiversity genomics programme that aims to sample a wide phylogenetic diversity of hosts, the new *Wolbachia* genomes presented here more than double the number of different host species from which *Wolbachia* genomes have been assembled. The assembled genomes include the first complete representatives isolated from Odonata (damselflies) and Orthoptera (crickets). In 16 additional datasets, we identified likely *Wolbachia* content but were not able to produce credible genome assemblies (see S1 Data and S2 Table). This was usually because the *Wolbachia* sequence was present in very low effective coverage (approximately threefold), but in some samples, no credible assembly was generated despite high coverage. These datasets may contain multiple recombining strains or contain large insertions in the host genome and deserve further exploration.

The distribution of *Wolbachia* in insect hosts is a function of the balance between retention through cospeciation (vertical transmission of *Wolbachia* to daughters of the host species), acquisition through horizontal transmission (where strains move between host species), and events of loss. Transmission among insect hosts was the dominant pattern underpinning *Wolbachia* distribution. We note that previous work has suggested that horizontal transmission rather than cospeciation may even explain the presence of closely related *Wolbachia* infecting closely related taxa. For example, genomic divergence between closely related *Wolbachia* in sister *Drosophila* species was too low to be the product of independent evolution since the last common ancestor of the flies [52,53]. However, we identified two features of the distribution, one local and one general, which are of note. Lepidoptera were more likely to be infected with supergroup B *Wolbachia* than A, and Hymenoptera, Diptera, and Coleoptera were more likely to be infected with supergroup A strains. Multilocus sequence typing (MLST) has previously shown that supergroup B is the most common *Wolbachia* type in Lepidoptera [22,31–33]. This suggests some nonexclusive specialisation of *Wolbachia* on their hosts, which may be driven by the interaction of *Wolbachia* and host genetics and/or a distinct set of ecological transmission routes in each insect group. Many of our genomes derived from insects were collected at one site, the Wytham Woods Genomic Observatory (S2 Fig), but this subset was no more closely related than other genomes from widely separated sites (S5 Fig). It is likely that the mobility of hosts, including through seasonal migration, means that sampling from one geographical site is a valid approximation of more global sampling. Close ecological association between host species may promote sharing of *Wolbachia* isolates and localised genetic exchange, for example, within predator–prey systems. The close similarity of *Wolbachia* genomes from *Andrena* solitary bees and their *Nomada* cuckoo bee kleptoparasites (Fig 4C, inset) and the shared occurrence of the biotin synthesis operon (Fig 4C) may be a case of transmission within an ecological network. The presence of the biotin operon in *Wolbachia* of insects that largely or solely feed on low-protein plant fluids (nectar or phloem) suggests that *Wolbachia* may be offering nutritional support to their hosts [54] and thus that this cluster of genomes may have been positively selected for their mutualist tendencies.

*Wolbachia* can promote reproductive success of their female hosts [1,2], and thus their own Darwinian fitness, through reproductive manipulations such as CI. The loci underpinning CI are a diverse set of toxin–antitoxin gene pairs. Our survey of *Wolbachia* identified many additional CI gene pairs, mainly of the I Cid type and mostly associated with WO phage. Many genomes had more than one toxin–antitoxin pair, and some individual hosts were infected with multiple *Wolbachia* strains carrying different CI gene pairs. These CI genes likely mediate conflict between *Wolbachia* strains and the ecosystem of toxin deposition and rescue in individual zygotes must be complex [46,55,56]. Interestingly, we identified CI gene pairs next to 5 of the 14 biotin synthesis operons, suggesting that the mobile elements that transduce this presumably mutualist physiology are also engaged in CI conflict.

One striking feature of the genomes assembled from the HiFi reads was that their average span was approximately 10% greater than the average size of previously assembled *Wolbachia* genomes. As we also observed a correlation between content of WO phage in the genome and genome size (Fig 5B), we speculate that the lower average size of previous assemblies may be because the presence of near-identical segments of phage and other mobile elements led to collapse of repeats and artificial underestimation of true genome size. This underestimation of genome size may also have biased understanding of WO phage diversity and of the diversity of genes that can be transduced by the phage. WO phage carry genes necessary for production of phage particles and cargo genes that have been hypothesised to form an EAM [40,41]. The increased genome size and increased resolution of WO phage copies might also mean increased gene content and diversity and an increased set of common EAM loci. We estimated the pan-proteome of A and B supergroup strains and found that the supergroup A had a higher pan-proteome but a smaller core proteome than supergroup B. Coupled with the observation of host-association bias between these supergroups, and other major genomic features such as GC proportion, this suggests that these divergent groups have followed very distinct evolutionary trajectories, despite evidence for transduction of loci between supergroups, and perhaps have evolved distinct physiologies and host-manipulation or host-cooperation strategies. We note that the ANI between A and B supergroup strains, and between strains from all supergroups, is relatively low (within-supergroup identity >93%, between-supergroup identity <88%). This pattern of significant phylogenetic separation between supergroups suggests, as others have noted, that these supergroups have the features expected of bacterial species [37].

The DToL project [16] is one of a growing constellation of biodiversity genomics initiatives worldwide that, under the banner of the Earth BioGenome Project [57], intend to “sequence life for the future of life” (https://www.earthbiogenome.org). These projects, based around ecological, regional, or taxonomic lists of target species, will lay the foundations for biological research, bioindustry, and conservation for the next decades. While their focus is to generate reference genomes for eukaryotic species, these projects will also yield critical resources for the study of the microbial cobionts—mutualists, pathogens, parasites, and commensals—which live on and in eukaryotic organisms. Our understanding of *Wolbachia* and other common endosymbionts will thrive on a rich harvest of cobiont genomes from the tens to hundreds of thousands of host genomes that will be generated in the next decade. The assembly of 110 high-quality *Wolbachia* genomes shows the power of the long read data now being generated and the analytic approach that allowed these low complexity metagenomes to be effectively separated into their constituent parts. Analysis of these genomes revealed a propensity to infect different insect orders among supergroups, while simultaneously pinpointing to several host switching events during the course of the *Wolbachia* pandemic. Moreover, we observed that genome size in *Wolbachia* is correlated with the abundance of copies of bacteriophage WO.

## Methods

### Detection and assembly of *Wolbachia* genomes from DToL species data

DToL raw data are generated from whole or partial single specimens and thus contain sequence from any cobionts in or on the specimen at the time of sampling. We screened data for 368 insect genomes generated by the DToL project [16] for the presence of the intracellular endosymbiont *Wolbachia* (S1 Table) using a marker gene scan approach by searching for the SSU rRNA locus. The prokaryotic 16S rRNA alignment from RFAM (RF00177) [58] was transformed into a HMMER profile, and the profile was used to screen contigs with nhmmscan [59]. We defined a positive match as having an e-value <10^−150^ or an aligned length of >1,000 nucleotides. Putative positive regions were extracted from the sequences and compared to the SILVA SSU database (version 138.1) [60] using sina [61]. Matches were filtered to retain only those with >90% identity. Taxonomic classification of each positive was determined via a consensus rule of 80% of the top 20 best hits, using both the NCBI [62] and SILVA [63] taxonomies.

For *Wolbachia*-positive samples, all PacBio HiFi reads were analysed using kraken2 [64] with a custom database consisting of a genome from a species closely related to the host, all RefSeq genomes of Anaplasmataceae, and reference genomes of additionally detected cobionts downloaded using NCBI datasets and masked using dustmasker [65]. Horizontal transfer of fragments of endosymbiont and organellar DNA to the nuclear genome is a common phenomenon. To avoid inadvertently classifying nuclear *Wolbachia* insertions (NUWTs) as deriving from an independent bacterial replicon, *Wolbachia* reads identified by kraken2 were mapped to the insect genome assembly, and only contigs fully covered by these reads were retained. The *Wolbachia* reads were also independently reassembled using several assembly tools: flye (version 2.9) (flye—pacbio-hifi {reads} -o {dir} -t {threads}—asm-coverage 50—genome-size 1.6m —scaffold) [66], hifiasm (version 0.14) (hifiasm -o {prefix} -t {threads} {reads} -D 10 -l 1 -s 0.999) [67], and hifiasm-meta (version 0.1-r022) (hifiasm_meta -o {prefix} -t {threads} {reads} -l 1) [68]. The several assemblies generated for each sample were ranked based on their completeness using BUSCO version 5.2.2 [69] and the Rickettsiales_odb10 dataset, alignment to reference genomes using nucmer (version 4.0.0) [70], evenness of coverage, and circularity. The best (most complete, single-contig circular preferred) assembly per sample was chosen. For samples where 10X Genomics Chromium data were available, polishing was performed using FreeBayes-called variants [71] from 10X short reads aligned with LongRanger. The host origin, span, and completeness of all *Wolbachia* detected are presented in S2 Table.

### Collation of *Wolbachia* genome dataset, gene prediction, and orthology inference

All available *Wolbachia* genomes were downloaded from NCBI GenBank on 01/02/2022 and supplemented with assemblies generated from short-read insect datasets by Scholz and colleagues [14]. This dataset contained replicate genomes for very closely related *Wolbachia* from the same host, and many fragmented and partial assemblies. Only the most contiguous assembly per host species was retained. These genomes were renamed using the schema “R_Xyz_GenSpec_§”, where Xyz is the first three letters of the insect order of the host, GenSpec is an abbreviation derived from the generic and specific epithets of the host, and § indicates the supergroup. Retained assemblies were assessed for the presence of contamination by performing a contig analysis by kraken2 using a database of only circular *Wolbachia* genomes. A list of all removed contigs can be found in S3 Table. Furthermore, we only included database-sourced *Wolbachia* genomes with at least 90% BUSCO completeness [69] and at most 3% duplication with the Rickettsiales_odb10 dataset (S3 Table). The exception to this filtering was the inclusion of genomes belonging to the most divergent supergroup S.

All of the publicly available and newly assembled genomes were annotated using Prodigal (version 2.6.3) [29]. Protein families were inferred using OrthoFinder (version 2.4.0) [30]. We identified 624 protein families, which were single-copy in more than 95% of all *Wolbachia* genomes. These were individually aligned using mafft in automatic mode (version 7.490) [72]. Individual maximum likelihood gene trees were calculated using iqtree (version 2.1.4) (iqtree -s {alignment} -nt {threads}) [73], and coalescence of these gene trees was determined using ASTRAL (version 5.7.4) [74]. The individual alignments were trimmed using trimAl (version 1.4) [75] and concatenated to form a supermatrix. This was used to infer a maximum likelihood phylogeny with iqtree using 1,000 ultrafast bootstrap approximation iterations (version 2.1.4) (iqtree -s {supermatrix} -m LG+G4 -bb 1000 -nt {threads}) [73]. The insect topology was subsampled from Chesters [76]. Incongruence in topology between the insect host and *Wolbachia*, host phylogeny was determined with ggtree in R [77].

### Intrinsic genomic properties

All circular genomes were rotated to start with HemE (OG0000716) on the positive strand, as this gene is located next to the origin of replication [78]. All pairwise alignments were calculated using nucmer (version 4.0.0) [70], and breakpoints were inferred and adjusted for the aligned coverage. Whole-genome average nucleotide diversity was calculated using FastANI (version 1.33) [79]. GC and GC skew index values were calculated for all genomes using SkewIT [34].

### Gene content analysis

To functionally annotate predicted genes, both Prokka (version 1.14.6) [80] and InterProScan (version 5.54–87.0) [81] were run. The synteny plot of the biotin locus was created using gggenes [82]. All six genes that make up the biotin locus (BioA-D, BioF, BioH) were individually aligned with mafft in automatic mode (version 7.490) [72] and transformed into a concatenated nucleotide alignment. A phylogenetic tree was built using the model GTR+F+G4 in iqtree (version 2.1.4) [73]. Genes responsible for CI were identified by a BLAST search [83] using the following genes as queries: CidA: WP_010962721.1, WP_182158704.1, WP_012673228.1, WP_006014162.1, CAQ54402.1, NZ_MUIX01000001.1_1324, OAM06111.1; CifB: WP_010962722.1, WP_182158703.1, WP_012673227.1, WP_006014164.1, CAQ54403.1, NZ_MUIX01000001.1_1323, OAM06112.1. Moreover, additional CifB type V genes were added as reference genes (Diachasma_alloeum_pair1, Diploeciton_nevermanni_pair5, wBor_pair2, wStri_pair1, wStri_pair2 and wTri-2_pair1). Only pairs of identified neighbouring genes (e-value 1 × 10^−30^, coverage 80% to 120%) were retained. Both CifA and CifB were aligned using mafft in automatic mode (version 7.490) [72], followed by maximum likelihood estimation using iqtree (version 2.1.4) (iqtree -s {alignment} -nt {threads} -bb 10000).

### WO prophage analysis

A list of known prophage sequences was generated based on annotated regions described in the literature [41,44,84] (S4 Table) for a set of genomes (R_Dip_DroSim_A, R_Hym_NasVit_A, R_Dip_DroAna_A, R_Dip_HaeIrr_A, R_Hym_CerSol_A, and R_Hym_WiePum_A) and linked to their respective gene families. Each *Wolbachia* genome was screened for continuous stretches of linked prophage genes with at most five other genes in-between, and these were annotated as prophage regions if they contained at least one gene from one of the four core phage modules (head, baseplate, tail, and fibre). This permitted detection of novel prophage-associated genes. Regions that contained at least 5 of 6 head, 7 of 8 baseplate, 5 of 6 fibre, and 5 of 6 tail module genes were deemed putatively complete. Genomic maps of prophage integration were created with circos [85]. Phylogenetic generalised least squares analyses were performed to assess the correlation between prophage length and genome size using the ape R package [86], using a Brownian model of evolution and the phylogenetic tree in Fig 2A. R squared values were calculated using the package rr2 [87].

## Supporting information

S1 Data Supplementary Data. (XLSX)

S1 Table DToL screened genomes. (PDF)

S2 Table Overview detected *Wolbachia* genomes. (PDF)

S3 Table *Wolbachia* reference genomes. (PDF)

S4 Table Prophage modules. (PDF)

S5 Table Summary toxin genes. (PDF)

S1 Fig Selected tissue and incidence of *Wolbachia*. Selected tissue and incidence of *Wolbachia* presence (green) and absence (purple) of DToL samples.
(PDF)

S2 Fig Sampling locations and incidence of *Wolbachia*. Sampling locations and incidence of *Wolbachia* presence (green) and absence (purple) of DToL samples from Britain and Ireland. The map was drawn using the maps library (version 3.4.0) in R, which imports data from the public domain (Natural Earth project) (https://www.naturalearthdata.com/downloads/50m-physical-vectors/). The size of the pie charts reflects the number of collected samples per location. Most samples came from Wytham Woods Genomic Observatory near Oxford. The data underlying this Figure can be found in S1 Data.
(PDF)

S3 Fig Contiguity and genome size distribution of *Wolbachia*. **(A**, **B)** Contiguity and genome size distribution of *Wolbachia* genomes assembled in this study (black) vs. reference genomes from other projects available in NCBI (grey). The data underlying this Figure can be found in S1 Data. (**C**) Genome size distribution of *Wolbachia*. Supergroups A (above) and B (below), in this study (black) and reference genomes from other projects available in NCBI (grey) were compared by Wilcoxon rank sum test. The data underlying this Figure can be found in S1 Data.
(PDF)

S4 Fig Phylogeny of supergroup A and B *Wolbachia*. Phylogeny of supergroup A and B *Wolbachia*, visualised with the root placed between the A and B supergroups and the remaining supergroups (C, D, E, F, J, S; nodes collapsed as grey wedge), highlighting nodes with bootstrap value higher than 80 with a black label.
(PDF)

S5 Fig Average nucleotide identity between Wytham Woods specimens. Distribution of average nucleotide identity (ANI) between pairs of *Wolbachia* genomes if specimens were both sampled from Wytham Woods (upper panel) or any other locality (lower panel). Distributions are separated by the classification of the two genomes, i.e., both belonging to supergroup A, both belonging to supergroup B, comparisons of A with B, or comparisons between other supergroups. The data underlying this Figure can be found in S1 Data.
(PDF)

S6 Fig Predicted proteome size in *Wolbachia*. Number of predicted protein-coding genes for *Wolbachia* supergroups A (above) and B (below), in this study (black) and reference genomes from other projects available in NCBI (grey) were compared by Wilcoxon rank sum test. The data underlying this Figure can be found in S1 Data.
(PDF)

S7 Fig Strain-specific proteins are not generally associated with WO phage. Percentage of protein-coding genes present in WO prophage regions versus percentage of strain-specific protein-coding genes in those regions of *Wolbachia* genomes with at least 10 strain-specific genes. Size of points is reflective of the total number of strain-specific genes. Linear regression line with confidence interval is displayed. The data underlying this Figure can be found in S1 Data.
(PDF)

S8 Fig Comparison of the phylogenies of biotin synthesis clusters and the *Wolbachia* strains that contain them. Comparison between phylogenies of *Wolbachia* genomes containing the biotin locus, based on tree in Fig 2A (left) and a phylogeny inferred from the six nucleotide genes constituting the biotin synthesis operon (BioA-D, BioF, BioH) (right). Internal nodes with bootstrap support higher than 80 are highlighted with black circles.
(PDF)

S9 Fig Phylogenetic representation of detected CifA genes. Phylogeny of CifA toxin genes, highlighting nodes with a bootstrap value higher than 80 with a circle.
(PDF)

S10 Fig Phylogenetic representation of detected CifB genes. Phylogeny of CifB toxin genes, highlighting nodes with a bootstrap value higher than 80 with a circle.
(PDF)

S11 Fig Phylogenetic representation of detected TcA genes. Phylogeny of TcA toxin genes, highlighting nodes with a bootstrap value higher than 80 with a circle.
(PDF)

S12 Fig Phylogenetic representation of detected TcB-C genes. Phylogeny of TcB-C toxin genes, highlighting nodes with a bootstrap value higher than 80 with a circle.
(PDF)

S13 Fig Phylogenetic representation of detected ParD genes. Phylogeny of ParD toxin genes, highlighting nodes with a bootstrap value higher than 80 with a circle.
(PDF)

S14 Fig Phylogenetic representation of detected ParE genes. Phylogeny of ParE toxin genes, highlighting nodes with a bootstrap value higher than 80 with a circle.
(PDF)

S15 Fig Phylogenetic representation of detected FIC genes. Phylogeny of FIC toxin genes, highlighting nodes with a bootstrap value higher than 80 with a circle.
(PDF)

## Abbreviations

ABC: ATP-binding cassette
ANI: average nucleotide identity; CI, cytoplasmic incompatibility
DToL: Darwin Tree of Life
EAM: eukaryotic association module
INSDC: International Nucleotide Sequence Database Collaboration
MAG: metagenome-assembled genome
MLST: multilocus sequence typing
SSU rRNA: small subunit ribosomal RNA

## References

[1] Yen JH, Barr AR. New Hypothesis of the Cause of Cytoplasmic Incompatibility in Culex pipiens L. Nature. 1971 Aug;232(5313):657–658. doi: 10.1038/232657a0 4937405

[2] Yen JH, Barr AR. The etiological agent of cytoplasmic incompatibility in Culex pipiens. Journal of Invertebrate Pathology. 1973 Sep;22(2):242–50. doi: 10.1016/0022-2011(73)90141-9 4206296

[3] Bordenstein SR, O’Hara FP, Werren JH. Wolbachia-induced incompatibility precedes other hybrid incompatibilities in Nasonia. Nature. 2001 Feb 8;409(6821):707–10. doi: 10.1038/35055543 11217858

[4] Hurst GDD, Jiggins FM, Hinrich Graf von der Schulenburg J, Bertrand D, West SA, Goriacheva II, et al. Male–killing Wolbachia in two species of insect. Proc R Soc Lond B. 1999 Apr 7;266(1420):735–40. doi: 10.1098/rspb.1999.0698

[5] Stouthamer R, Breeuwer JAJ, Luck RF, Werren JH. Molecular identification of microorganisms associated with parthenogenesis. Nature. 1993 Jan;361(6407):66–8. doi: 10.1038/361066a0 7538198

[6] Hornett EA, Charlat S, Duplouy AMR, Davies N, Roderick GK, Wedell N, et al. Evolution of Male-Killer Suppression in a Natural Population. PLoS Biol. 2006 Aug 22;4(9):e283. doi: 10.1371/journal.pbio.0040283 16933972 PMC1551922

[7] Werren JH, Baldo L, Clark ME. Wolbachia: master manipulators of invertebrate biology. Nat Rev Microbiol. 2008 Oct;6(10):741–51. doi: 10.1038/nrmicro1969 18794912

[8] Nikoh N, Hosokawa T, Moriyama M, Oshima K, Hattori M, Fukatsu T. Evolutionary origin of insect–*Wolbachia* nutritional mutualism. Proc Natl Acad Sci USA. 2014 Jul 15;111(28):10257–62. doi: 10.1073/pnas.1409284111 24982177 PMC4104916

[9] Pan X, Pike A, Joshi D, Bian G, McFadden MJ, Lu P, et al. The bacterium Wolbachia exploits host innate immunity to establish a symbiotic relationship with the dengue vector mosquito Aedes aegypti. ISME J. 2018 Jan;12(1):277–88. doi: 10.1038/ismej.2017.174 29099491 PMC5739022

[10] Hoerauf A, Mand S, Adjei O, Fleischer B, Büttner DW. Depletion of wolbachia endobacteria in Onchocerca volvulus by doxycycline and microfilaridermia after ivermectin treatment. Lancet. 2001 May;357(9266):1415–6. doi: 10.1016/S0140-6736(00)04581-5 11356444

[11] Zug R, Hammerstein P. Still a Host of Hosts for Wolbachia: Analysis of Recent Data Suggests That 40% of Terrestrial Arthropod Species Are Infected. PLoS ONE. 2012 Jun 7;7(6):e38544. doi: 10.1371/journal.pone.0038544 22685581 PMC3369835

[12] Zhou W, Rousset F, O’Neill S. Phylogeny and PCR–based classification of Wolbachia strains using wsp gene sequences. Proc R Soc Lond B. 1998 Mar 22;265(1395):509–15. doi: 10.1098/rspb.1998.0324 9569669 PMC1688917

[13] Glowska E, Dragun-Damian A, Dabert M, Gerth M. New Wolbachia supergroups detected in quill mites (Acari: Syringophilidae). Infect Genet Evol. 2015 Mar;30:140–6. doi: 10.1016/j.meegid.2014.12.019 25541519

[14] Scholz M, Albanese D, Tuohy K, Donati C, Segata N, Rota-Stabelli O. Large scale genome reconstructions illuminate Wolbachia evolution. Nat Commun. 2020 Dec;11(1):5235. doi: 10.1038/s41467-020-19016-0 33067437 PMC7568565

[15] Pascar J, Chandler CH. A bioinformatics approach to identifying Wolbachia infections in arthropods. PeerJ. 2018 Sep 3;6:e5486. doi: 10.7717/peerj.5486 30202647 PMC6126470

[16] The Darwin Tree of Life Project Consortium. Sequence locally, think globally: The Darwin Tree of Life Project. Proc Natl Acad Sci U S A. 2022 Jan 25;119(4):e2115642118. doi: 10.1073/pnas.2115642118 35042805 PMC8797607

[17] Challis R, Richards E, Rajan J, Cochrane G, Blaxter M. BlobToolKit–Interactive Quality Assessment of Genome Assemblies. G3 (Bethesda). 2020 Apr 1;10(4):1361–74. doi: 10.1534/g3.119.400908 32071071 PMC7144090

[18] Eren AM, Esen ÖC, Quince C, Vineis JH, Morrison HG, Sogin ML, et al. Anvi’o: an advanced analysis and visualization platform for ‘omics data. PeerJ. 2015 Oct 8;3:e1319. doi: 10.7717/peerj.1319 26500826 PMC4614810

[19] Regan T, Barnett MW, Laetsch DR, Bush SJ, Wragg D, Budge GE, et al. Characterisation of the British honey bee metagenome. Nat Commun. 2018 Dec;9(1):4995. doi: 10.1038/s41467-018-07426-0 30478343 PMC6255801

[20] Hilgenboecker K, Hammerstein P, Schlattmann P, Telschow A, Werren JH. How many species are infected with Wolbachia?–a statistical analysis of current data: Wolbachia infection rates. FEMS Microbiol Lett. 2008 Apr;281(2):215–20. doi: 10.1111/j.1574-6968.2008.01110.x 18312577 PMC2327208

[21] Ahmed MZ, Breinholt JW, Kawahara AY. Evidence for common horizontal transmission of Wolbachia among butterflies and moths. BMC Evol Biol. 2016 Dec;16(1):118. doi: 10.1186/s12862-016-0660-x 27233666 PMC4882834

[22] West SA, Cook JM, Werren JH, Godfray HCJ. *Wolbachia* in two insect host–parasitoid communities. Mol Ecol. 1998 Nov;7(11):1457–65. doi: 10.1046/j.1365-294x.1998.00467.x 9819901

[23] Duron O, Bouchon D, Boutin S, Bellamy L, Zhou L, Engelstädter J, et al. The diversity of reproductive parasites among arthropods: Wolbachia do not walk alone. BMC Biol. 2008 Dec;6(1):27. doi: 10.1186/1741-7007-6-27 18577218 PMC2492848

[24] Weinert LA, Araujo-Jnr EV, Ahmed MZ, Welch JJ. The incidence of bacterial endosymbionts in terrestrial arthropods. Proc R Soc B. 2015 May 22;282(1807):20150249. doi: 10.1098/rspb.2015.0249 25904667 PMC4424649

[25] Strunov A, Schmidt K, Kapun M, Miller WJ. Restriction of *Wolbachia* Bacteria in Early Embryogenesis of Neotropical *Drosophila* Species via Endoplasmic Reticulum-Mediated Autophagy. mBio. 2022 Apr 26;13(2):e03863–21. doi: 10.1128/mbio.03863-21 35357208 PMC9040723

[26] Kamath AD, Deehan MA, Frydman HM. Polar cell fate stimulates *Wolbachia* intracellular growth. Development. 2018 Jan 1;dev.158097. doi: 10.1242/dev.158097 29467241 PMC5897597

[27] Savill P, Perrins C, Kirby K, Fisher N. Wytham woods: Oxford’s ecological laboratory. 1. publ. in paperback. Oxford: Oxford Univ. Press; 2011. p. 263.

[28] Misof B, Liu S, Meusemann K, Peters RS, Donath A, Mayer C, et al. Phylogenomics resolves the timing and pattern of insect evolution. Science. 2014 Nov 7;346(6210):763–7. doi: 10.1126/science.1257570 25378627

[29] Hyatt D, Chen GL, LoCascio PF, Land ML, Larimer FW, Hauser LJ. Prodigal: prokaryotic gene recognition and translation initiation site identification. BMC Bioinformatics. 2010 Dec;11(1):119. doi: 10.1186/1471-2105-11-119 20211023 PMC2848648

[30] Emms DM, Kelly S. OrthoFinder: phylogenetic orthology inference for comparative genomics. Genome Biol. 2019 Dec;20(1):238. doi: 10.1186/s13059-019-1832-y 31727128 PMC6857279

[31] Russell JA, Goldman-Huertas B, Moreau CS, Baldo L, Stahlhut JK, Werren JH, et al. Specialization and geographic isolation among *Wolbachia* symbionts from ants and lycaenid butterflies. Evolution. 2009 Mar;63(3):624–40. doi: 10.1111/j.1558-5646.2008.00579.x 19054050

[32] Werren JH, Windsor DM. *Wolbachia* infection frequencies in insects: evidence of a global equilibrium? Proc R Soc Lond B. 2000 Jul 7;267(1450):1277–85. doi: 10.1098/rspb.2000.1139 10972121 PMC1690679

[33] Tagami Y, Miura K. Distribution and prevalence of Wolbachia in Japanese populations of Lepidoptera: Wolbachia in Japanese Lepidoptera. Insect Mol Biol. 2004 Jul 20;13(4):359–64. doi: 10.1111/j.0962-1075.2004.00492.x 15271207

[34] Lu J, Salzberg SL. SkewIT: The Skew Index Test for large-scale GC Skew analysis of bacterial genomes. PLoS Comput Biol. 2020 Dec 4;16(12):e1008439. doi: 10.1371/journal.pcbi.1008439 33275607 PMC7717575

[35] Comandatore F, Cordaux R, Bandi C, Blaxter M, Darby A, Makepeace BL, et al. Supergroup C Wolbachia, mutualist symbionts of filarial nematodes, have a distinct genome structure. Open Biol. 2015 Dec;5(12):150099. doi: 10.1098/rsob.150099 26631376 PMC4703054

[36] Mahmood S, Nováková E, Martinů J, Sychra O, Hypša V. Extremely reduced supergroup F *Wolbachia*: transition to obligate insect symbionts [Internet]. Evol Biol. 2021 Oct [cited 2022 Aug 31]. Available from: http://biorxiv.org/lookup/doi/10.1101/2021.10.15.464041.

[37] Ellegaard KM, Klasson L, Näslund K, Bourtzis K, Andersson SGE. Comparative Genomics of Wolbachia and the Bacterial Species Concept. PLoS Genet. 2013 Apr 4;9(4):e1003381. doi: 10.1371/journal.pgen.1003381 23593012 PMC3616963

[38] Gerth M, Bleidorn C. Comparative genomics provides a timeframe for Wolbachia evolution and exposes a recent biotin synthesis operon transfer. Nat Microbiol. 2017 Mar;2(3):16241. doi: 10.1038/nmicrobiol.2016.241 28005061

[39] Gerth M, Röthe J, Bleidorn C. Tracing horizontal Wolbachia movements among bees (Anthophila): a combined approach using multilocus sequence typing data and host phylogeny. Mol Ecol. 2013 Dec;22(24):6149–62. doi: 10.1111/mec.12549 24118435

[40] Bordenstein SR, Bordenstein SR. Eukaryotic association module in phage WO genomes from Wolbachia. Nat Commun. 2016 Dec;7(1):13155. doi: 10.1038/ncomms13155 27727237 PMC5062602

[41] Bordenstein SR, Bordenstein SR. Widespread phages of endosymbionts: Phage WO genomics and the proposed taxonomic classification of Symbioviridae. PLoS Genet. 2022 Jun 6;18(6):e1010227. doi: 10.1371/journal.pgen.1010227 35666732 PMC9203015

[42] Gavotte L, Henri H, Stouthamer R, Charif D, Charlat S, Bouletreau M, et al. A Survey of the Bacteriophage WO in the Endosymbiotic Bacteria Wolbachia. Mol Biol Evol. 2006 Nov 13;24(2):427–35. doi: 10.1093/molbev/msl171 17095536

[43] Massey JH, Newton ILG. Diversity and function of arthropod endosymbiont toxins. Trends Microbiol. 2022 Feb;30(2):185–98. doi: 10.1016/j.tim.2021.06.008 34253453 PMC8742837

[44] LePage DP, Metcalf JA, Bordenstein SR, On J, Perlmutter JI, Shropshire JD, et al. Prophage WO genes recapitulate and enhance Wolbachia-induced cytoplasmic incompatibility. Nature. 2017 Mar;543(7644):243–7. doi: 10.1038/nature21391 28241146 PMC5358093

[45] Beckmann JF, Ronau JA, Hochstrasser M. A Wolbachia deubiquitylating enzyme induces cytoplasmic incompatibility. Nat Microbiol. 2017 May;2(5):17007. doi: 10.1038/nmicrobiol.2017.7 28248294 PMC5336136

[46] Martinez J, Klasson L, Welch JJ, Jiggins FM. Life and Death of Selfish Genes: Comparative Genomics Reveals the Dynamic Evolution of Cytoplasmic Incompatibility. Mol Biol Evol. 2021 Jan 4;38(1):2–15. doi: 10.1093/molbev/msaa209 32797213 PMC7783169

[47] Beckmann JF, Bonneau M, Chen H, Hochstrasser M, Poinsot D, Merçot H, et al. The Toxin–Antidote Model of Cytoplasmic Incompatibility: Genetics and Evolutionary Implications. Trends Genet. 2019 Mar;35(3):175–85. doi: 10.1016/j.tig.2018.12.004 30685209 PMC6519454

[48] Dy RL, Przybilski R, Semeijn K, Salmond GPC, Fineran PC. A widespread bacteriophage abortive infection system functions through a Type IV toxin–antitoxin mechanism. Nucleic Acids Res. 2014 Apr;42(7):4590–605. doi: 10.1093/nar/gkt1419 24465005 PMC3985639

[49] Kumar S, Blaxter ML. Simultaneous genome sequencing of symbionts and their hosts. Symbiosis. 2011;55 (3):119–126. doi: 10.1007/s13199-012-0154-6 22448083 PMC3294205

[50] Almeida A, Nayfach S, Boland M, Strozzi F, Beracochea M, Shi ZJ, et al. A unified catalog of 204,938 reference genomes from the human gut microbiome. Nat Biotechnol. 2021 Jan;39(1):105–14. doi: 10.1038/s41587-020-0603-3 32690973 PMC7801254

[51] Parks DH, Rinke C, Chuvochina M, Chaumeil PA, Woodcroft BJ, Evans PN, et al. Recovery of nearly 8,000 metagenome-assembled genomes substantially expands the tree of life. Nat Microbiol. 2017 Nov;2(11):1533–42. doi: 10.1038/s41564-017-0012-7 28894102

[52] Conner WR, Blaxter ML, Anfora G, Ometto L, Rota-Stabelli O, Turelli M. Genome comparisons indicate recent transfer of *w* Ri-like *Wolbachia* between sister species *Drosophila suzukii* and *D*. *subpulchrella*. Ecol Evol. 2017;7(22):9391–9404. doi: 10.1002/ece3.3449 29187976 PMC5696437

[53] Turelli M, Cooper BS, Richardson KM, Ginsberg PS, Peckenpaugh B, Antelope CX, et al. Rapid Global Spread of wRi-like Wolbachia across Multiple Drosophila. Curr Biol. 2018 Mar;28(6):963–971.e8. doi: 10.1016/j.cub.2018.02.015 29526588 PMC5882237

[54] Ju JF, Bing XL, Zhao DS, Guo Y, Xi Z, Hoffmann AA, et al. Wolbachia supplement biotin and riboflavin to enhance reproduction in planthoppers. ISME J. 2020 Mar;14(3):676–87. doi: 10.1038/s41396-019-0559-9 31767943 PMC7031331

[55] Lindsey ARI, Rice DW, Bordenstein SR, Brooks AW, Bordenstein SR, Newton ILG. Evolutionary Genetics of Cytoplasmic Incompatibility Genes cifA and cifB in Prophage WO of Wolbachia. Genome Biol Evol. 2018 Feb 1;10(2):434–451. doi: 10.1093/gbe/evy012 29351633 PMC5793819

[56] Shropshire JD, Leigh B, Bordenstein SR. Symbiont-mediated cytoplasmic incompatibility: What have we learned in 50 years? eLife. 2020 Sep 25;9:e61989. doi: 10.7554/eLife.61989 32975515 PMC7518888

[57] Lewin HA, Richards S, Lieberman Aiden E, Allende ML, Archibald JM, Bálint M, et al. The Earth BioGenome Project 2020: Starting the clock. Proc Natl Acad Sci U S A. 2022 Jan 25;119(4):e2115635118. doi: 10.1073/pnas.2115635118 35042800 PMC8795548

[58] Kalvari I, Nawrocki EP, Ontiveros-Palacios N, Argasinska J, Lamkiewicz K, Marz M, et al. Rfam 14: expanded coverage of metagenomic, viral and microRNA families. Nucleic Acids Res. 2021 Jan 8;49(D1):D192–200. doi: 10.1093/nar/gkaa1047 33211869 PMC7779021

[59] Eddy SR. Accelerated Profile HMM Searches. PLoS Comput Biol. 2011 Oct 20;7(10):e1002195. doi: 10.1371/journal.pcbi.1002195 22039361 PMC3197634

[60] Quast C, Pruesse E, Yilmaz P, Gerken J, Schweer T, Yarza P, et al. The SILVA ribosomal RNA gene database project: improved data processing and web-based tools. Nucleic Acids Res. 2013 Jan 1;41(D1):D590–6. doi: 10.1093/nar/gks1219 23193283 PMC3531112

[61] Pruesse E, Peplies J, Glöckner FO. SINA: Accurate high-throughput multiple sequence alignment of ribosomal RNA genes. Bioinformatics. 2012 Jul 15;28(14):1823–9. doi: 10.1093/bioinformatics/bts252 22556368 PMC3389763

[62] Schoch CL, Ciufo S, Domrachev M, Hotton CL, Kannan S, Khovanskaya R, et al. NCBI Taxonomy: a comprehensive update on curation, resources and tools. Database. 2020 Jan 1;2020:baaa062. doi: 10.1093/database/baaa062 32761142 PMC7408187

[63] Yilmaz P, Parfrey LW, Yarza P, Gerken J, Pruesse E, Quast C, et al. The SILVA and “All-species Living Tree Project (LTP)” taxonomic frameworks. Nucleic Acids Res. 2014 Jan;42(D1):D643–8. doi: 10.1093/nar/gkt1209 24293649 PMC3965112

[64] Wood DE, Lu J, Langmead B. Improved metagenomic analysis with Kraken 2. Genome Biol. 2019 Nov 28;20(1):257. doi: 10.1186/s13059-019-1891-0 31779668 PMC6883579

[65] Morgulis A, Gertz EM, Schäffer AA, Agarwala R. A fast and symmetric DUST implementation to mask low-complexity DNA sequences. J Comput Biol. 2006 Jun;13(5):1028–40. doi: 10.1089/cmb.2006.13.1028 16796549

[66] Kolmogorov M, Yuan J, Lin Y, Pevzner PA. Assembly of long, error-prone reads using repeat graphs. Nat Biotechnol. 2019 May;37(5):540–6. doi: 10.1038/s41587-019-0072-8 30936562

[67] Cheng H, Concepcion GT, Feng X, Zhang H, Li H. Haplotype-resolved de novo assembly using phased assembly graphs with hifiasm. Nat Methods. 2021 Feb;18(2):170–5. doi: 10.1038/s41592-020-01056-5 33526886 PMC7961889

[68] Feng X, Cheng H, Portik D, Li H. Metagenome assembly of high-fidelity long reads with hifiasm-meta. Nat Methods. 2022 Jun;19(6):671–4. doi: 10.1038/s41592-022-01478-3 35534630 PMC9343089

[69] Manni M, Berkeley MR, Seppey M, Simão FA, Zdobnov EM. BUSCO Update: Novel and Streamlined Workflows along with Broader and Deeper Phylogenetic Coverage for Scoring of Eukaryotic, Prokaryotic, and Viral Genomes. Mol Biol Evol. 2021 Oct 1;38(10):4647–54. doi: 10.1093/molbev/msab199 34320186 PMC8476166

[70] Marçais G, Delcher AL, Phillippy AM, Coston R, Salzberg SL, Zimin A. MUMmer4: A fast and versatile genome alignment system. PLoS Comput Biol. 2018 Jan 26;14(1):e1005944. doi: 10.1371/journal.pcbi.1005944 29373581 PMC5802927

[71] Garrison E, Marth G. Haplotype-based variant detection from short-read sequencing [Internet]. arXiv. 2012 Jul [cited 2022 Jun 13]. Report No.: arXiv:1207.3907. Available from: http://arxiv.org/abs/1207.3907.

[72] Katoh K, Standley DM. MAFFT Multiple Sequence Alignment Software Version 7: Improvements in Performance and Usability. Mol Biol Evol. 2013 Apr 1;30(4):772–80. doi: 10.1093/molbev/mst010 23329690 PMC3603318

[73] Minh BQ, Schmidt HA, Chernomor O, Schrempf D, Woodhams MD, von Haeseler A, et al. IQ-TREE 2: New Models and Efficient Methods for Phylogenetic Inference in the Genomic Era. Mol Biol Evol. 2020 May 1;37(5):1530–4. doi: 10.1093/molbev/msaa015 32011700 PMC7182206

[74] Mirarab S, Reaz R, Bayzid MdS, Zimmermann T, Swenson MS, Warnow T. ASTRAL: genome-scale coalescent-based species tree estimation. Bioinformatics. 2014 Sep 1;30(17):i541–8. doi: 10.1093/bioinformatics/btu462 25161245 PMC4147915

[75] Capella-Gutiérrez S, Silla-Martínez JM, Gabaldón T. trimAl: a tool for automated alignment trimming in large-scale phylogenetic analyses. Bioinformatics. 2009 Aug 1;25(15):1972–3. doi: 10.1093/bioinformatics/btp348 19505945 PMC2712344

[76] Chesters D. Construction of a Species-Level Tree of Life for the Insects and Utility in Taxonomic Profiling. Syst Biol. 2017 May 1;66(3):426–439;syw099. doi: 10.1093/sysbio/syw099 27798407 PMC5837528

[77] Yu G, Lam TTY, Zhu H, Guan Y. Two Methods for Mapping and Visualizing Associated Data on Phylogeny Using *Ggtree*. Mol Biol Evol. 2018 Dec 1;35(12):3041–3043. doi: 10.1093/molbev/msy194 30351396 PMC6278858

[78] Ioannidis P, Hotopp JCD, Sapountzis P, Siozios S, Tsiamis G, Bordenstein SR, et al. New criteria for selecting the origin of DNA replication in Wolbachia and closely related bacteria. BMC Genomics. 2007 Dec;8(1):182. doi: 10.1186/1471-2164-8-182 17584494 PMC1914354

[79] Jain C, Rodriguez-R LM, Phillippy AM, Konstantinidis KT, Aluru S. High throughput ANI analysis of 90K prokaryotic genomes reveals clear species boundaries. Nat Commun. 2018 Nov 30;9(1):5114. doi: 10.1038/s41467-018-07641-9 30504855 PMC6269478

[80] Seemann T. Prokka: rapid prokaryotic genome annotation. Bioinformatics. 2014 Jul 15;30(14):2068–9. doi: 10.1093/bioinformatics/btu153 24642063

[81] Quevillon E, Silventoinen V, Pillai S, Harte N, Mulder N, Apweiler R, et al. InterProScan: protein domains identifier. Nucleic Acids Res. 2005 Jul 1;33(Web Server):W116–20. doi: 10.1093/nar/gki442 15980438 PMC1160203

[82] Wilkins D. gggenes [Internet]. Available from: https://github.com/wilkox/gggenes.

[83] Altschul SF, Gish W, Miller W, Myers EW, Lipman DJ. Basic local alignment search tool. J Mol Biol. 1990 Oct;215(3):403–10. doi: 10.1016/S0022-2836(05)80360-2 2231712

[84] Miao Y heng, Xiao J hua, Huang D wei. Distribution and Evolution of the Bacteriophage WO and Its Antagonism With Wolbachia. Front Microbiol. 2020 Nov 13;11:595629. doi: 10.3389/fmicb.2020.595629 33281793 PMC7691483

[85] Krzywinski M, Schein J, Birol I, Connors J, Gascoyne R, Horsman D, et al. Circos: an information aesthetic for comparative genomics. Genome Res. 2009 Sep;19(9):1639–45. doi: 10.1101/gr.092759.109 19541911 PMC2752132

[86] Paradis E, Schliep K. ape 5.0: an environment for modern phylogenetics and evolutionary analyses in R. Bioinformatics. 2019 Feb 1;35(3):526–528. doi: 10.1093/bioinformatics/bty633 30016406

[87] Ives AR. R2s for Correlated Data: Phylogenetic Models, LMMs, and GLMMs. Harmon L, editor. Syst Biol. 2019 Mar 1;68(2):234–251. doi: 10.1093/sysbio/syy060 30239975

