# Supplementary material for: Phylogenomic analysis of Wolbachia genomes from the Darwin Tree of Life biodiversity genomics project
Source: PLoS Biol. 2023 Jan 23;21(1):e3001972. doi: 10.1371/journal.pbio.3001972 (PMC9894559; doi:10.1371/journal.pbio.3001972)
Supplement: S1 Table — (PDF) [file pbio.3001972.s002.pdf]

S1 Table. Overview of all DToL screened genomes

Scientific name	Species name	Taxonomic order	Biospecimen ID	Sex	Tissue
Abrostola tripartita	The Spectacle	Lepidoptera	SAMEA7520667	Male	HEAD | THORAX
Acleris emargana	Notch-wing Button	Lepidoptera	SAMEA7746613	Male	WHOLE ORGANISM
Acleris sparsana	Ashy button	Lepidoptera	SAMEA8603209	Male	WHOLE ORGANISM
Acrobasis consociella	Broad-barred Knot-horn	Lepidoptera	SAMEA7701461	Unknown	WHOLE ORGANISM
Acrobasis repandana	Warted Knot-horn	Lepidoptera	SAMEA7701659	Unknown	WHOLE ORGANISM
Acronicta aceris	The sycamore	Lepidoptera	SAMEA7701532	Female	ABDOMEN
Agonopterix arenella	Brindled Flat-body	Lepidoptera	SAMEA8603192	Male	WHOLE ORGANISM
Agonopterix heracliana	Common flat-body	Lepidoptera	SAMEA7701514	Unknown	WHOLE ORGANISM
Agonopterix subpropinquella	Ruddy Flat-body	Lepidoptera	SAMEA7746629	Male	WHOLE ORGANISM
Agriopis aurantiaria	Scarce umber	Lepidoptera	SAMEA8603214	Male	THORAX
Agriphila geniculea	Elbow-stripe Grass-veneer	Lepidoptera	SAMEA8603180	Female	WHOLE ORGANISM
Agriphila straminella	Straw Grass-veneer	Lepidoptera	SAMEA7701512	Unknown	WHOLE ORGANISM
Agriphila tristella	Common grass-veneer	Lepidoptera	SAMEA8603174	Male	THORAX
Agrochola circellaris	The brick	Lepidoptera	SAMEA8603201	Male	THORAX
Agrochola macilenta	Yellow-line quaker	Lepidoptera	SAMEA8603207	Female	THORAX
Allophyes oxyacanthae	Green-brindled crescent	Lepidoptera	SAMEA8603204	Male	THORAX
Amphipyra berbera	Svensson's copper underwing	Lepidoptera	SAMEA7701493	Male	ABDOMEN
Amphipyra tragopoginis	Mouse moth	Lepidoptera	SAMEA7520174	Male	HEAD | THORAX
Anthocharis cardamines	Orange tip	Lepidoptera	SAMEA7523110	Female	WHOLE ORGANISM
Apamea monoglypha	Dark arches	Lepidoptera	SAMEA7701555	Male	ABDOMEN
Apatura iris	Purple emperor	Lepidoptera	SAMEA7523112	Unknown	WHOLE ORGANISM
Apeira syringaria	Lilac beauty	Lepidoptera	SAMEA7520685	Male	HEAD | THORAX
Aplocera efformata	Lesser treble-bar	Lepidoptera	SAMEA8603170	Female	THORAX
Aporia crataegi	Black Veined White	Lepidoptera	SAMEA7523355	Male	WHOLE ORGANISM
Aporophyla lueneburgensis	Northern deep-brown dart	Lepidoptera	SAMEA8603194	Female	THORAX
Apotomis betuletana	Birch marble	Lepidoptera	SAMEA7701588	Male	WHOLE ORGANISM
Apotomis turbidana	White-shouldered Marble	Lepidoptera	SAMEA7520681	Unknown	WHOLE ORGANISM
Archips podanus	Large Fruit-tree Tortrix	Lepidoptera	SAMEA7701540	Unknown	WHOLE ORGANISM

Archips xylosteana	Variegated Golden Tortrix	Lepidoptera	SAMEA7701541	Unknown	WHOLE ORGANISM
Argyresthia goedartella	Golden Argent	Lepidoptera	SAMEA7520176	Unknown	WHOLE ORGANISM
Aricia agestis	Brown argus	Lepidoptera	SAMEA7523300	Male	WHOLE ORGANISM
Atethmia centrago	Centre-barred sallow	Lepidoptera	SAMEA7520177	Male	HEAD | THORAX
Autographa gamma	Silver Y	Lepidoptera	SAMEA7519848	Female	HEAD | THORAX
Autographa pulchrina	Beautiful Golden Y	Lepidoptera	SAMEA7520527	Female	HEAD | THORAX
Bembecia ichneumoniformis	Six-belted clearwing	Lepidoptera	SAMEA7701282	Male	ABDOMEN
Biston betularia	Peppered moth	Lepidoptera	SAMEA7520512	Male	THORAX | ABDOMEN
Blastobasis adustella	Dingy Dowd	Lepidoptera	SAMEA7520179	Female	WHOLE ORGANISM
Blastobasis lacticolella	London Dowd	Lepidoptera	SAMEA7519826	Male	WHOLE ORGANISM
Boloria selene	Small pearl-bordered fritillary	Lepidoptera	SAMEA7523131	Female	WHOLE ORGANISM
Calamotropha paludella	Bulrush Veneer	Lepidoptera	SAMEA7746625	Male	ABDOMEN
Campaea margaritaria	Light emerald	Lepidoptera	SAMEA7701535	Male	ABDOMEN
Camptogramma bilineatum	Yellow shell	Lepidoptera	SAMEA7701528	Unknown	WHOLE ORGANISM
Caradrina clavipalpis	Pale mottled willow	Lepidoptera	SAMEA8603187	Male	THORAX
Caradrina kadenii	Clancy’s Rustic	Lepidoptera	SAMEA8534280	Female	ABDOMEN
Carcina quercana	Long-horned flat-body	Lepidoptera	SAMEA7519850	Male	WHOLE ORGANISM
Catocala fraxini	Cliften non-pareil	Lepidoptera	SAMEA8603175	Male	THORAX
Catoptria pinella	Pearl Grass-veneer	Lepidoptera	SAMEA7701506	Unknown	WHOLE ORGANISM
Celastrina argiolus	Holly brown	Lepidoptera	SAMEA7523268	Male	WHOLE ORGANISM
Chloroclysta siterata	Red-green carpet	Lepidoptera	SAMEA8603199	Male	THORAX
Chloroclystis v-ata	V-pug	Lepidoptera	SAMEA7701460	Unknown	WHOLE ORGANISM
Chrysoteuchia culmella	Garden Grass-veneer	Lepidoptera	SAMEA7701502	Male	WHOLE ORGANISM
Clostera curtula	Chocolate tip	Lepidoptera	SAMEA7520526	Female	ABDOMEN
Colias croceus	Clouded Yellow	Lepidoptera	SAMEA7523360	Female	THORAX
Colostygia pectinataria	Green carpet	Lepidoptera	SAMEA7520182	Unknown	WHOLE ORGANISM
Cosmia trapezina	The dun-bar	Lepidoptera	SAMEA7519851	Male	THORAX | ABDOMEN
Craniophora ligustri	The coronet	Lepidoptera	SAMEA7519852	Female	THORAX | ABDOMEN
Crocallis elinguaria	Scalloped oak	Lepidoptera	SAMEA7701527	Female	ABDOMEN
Cupido minimus	Small blue	Lepidoptera	SAMEA7523306	Male	WHOLE ORGANISM
Cyaniris semiargus	Mazarine blue	Lepidoptera	SAMEA7523311	Male	WHOLE ORGANISM

Cydia fagiglandana	Large Beech Piercer	Lepidoptera	SAMEA7701453	Unknown	WHOLE ORGANISM
Cydia splendana	Marbled Piercer	Lepidoptera	SAMEA7701547	Female	WHOLE ORGANISM
Deilephila porcellus	Small elephant Hawk-moth	Lepidoptera	SAMEA7520522	Male	ABDOMEN
Diachrysia chrysitis	Burnished brass moth	Lepidoptera	SAMEA8603181	Male	THORAX
Diarsia rubi	Small square-spot	Lepidoptera	SAMEA8603186	Female	THORAX
Ditula angustiorana	Red-barred Tortrix	Lepidoptera	SAMEA7701319	Unknown	WHOLE ORGANISM
Dryobotodes eremita	Brindled green	Lepidoptera	SAMEA8603190	Female	THORAX
Ecliptopera silaceata	Small phoenix	Lepidoptera	SAMEA7701534	Male	WHOLE ORGANISM
Ectropis crepuscularia	The engrailed	Lepidoptera	SAMEA7701526	Unknown	WHOLE ORGANISM
Eilema depressum	Buff footman	Lepidoptera	SAMEA7746611	Male	THORAX
Eilema sororcula	Orange Footman	Lepidoptera	SAMEA7631555	Male	WHOLE ORGANISM
Emmelina monodactyla	Common plume	Lepidoptera	SAMEA8603203	Female	WHOLE ORGANISM
Endotricha flammealis	Rosy tabby	Lepidoptera	SAMEA7519855	Female	WHOLE ORGANISM
Ennomos fuscantarius	Dusky thorn	Lepidoptera	SAMEA7520185	Male	HEAD | THORAX
Ennomos quercinarius	August thorn	Lepidoptera	SAMEA7701560	Male	ABDOMEN
Epagoge grotiana	Brown-barred Tortrix	Lepidoptera	SAMEA7701542	Unknown	WHOLE ORGANISM
Epinotia brunnichana	Large Birch Bell	Lepidoptera	SAMEA7701529	Unknown	WHOLE ORGANISM
Epirrhoe alternata	common carpet	Lepidoptera	SAMEA7701550	Unknown	WHOLE ORGANISM
Erannis defoliaria	Mottled umber	Lepidoptera	SAMEA7520367	Male	THORAX | ABDOMEN
Erebia aethiops	Scotch argus	Lepidoptera	SAMEA7523289	Female	WHOLE ORGANISM
Erebia ligea	Mountain ringlet	Lepidoptera	SAMEA7523313	Male	WHOLE ORGANISM
Erynnis tages	Dingy skipper	Lepidoptera	SAMEA7523299	Male	WHOLE ORGANISM
Eucosma campoliliana	Marbled Bell	Lepidoptera	SAMEA7701295	Unknown	WHOLE ORGANISM
Eucosma cana	Hoary Bell	Lepidoptera	SAMEA7701552	Unknown	WHOLE ORGANISM
Eulithis prunata	The Pheonix	Lepidoptera	SAMEA7701309	Male	ABDOMEN
Euphydryas aurinia	Marsh Fritillary	Lepidoptera	SAMEA7523466	Unknown	WHOLE ORGANISM
Eupithecia centaureata	Lime-speck pug	Lepidoptera	SAMEA7520186	Male	WHOLE ORGANISM
Eupithecia tripunctaria	White-spotted pug	Lepidoptera	SAMEA7701546	Unknown	WHOLE ORGANISM
Euplexia lucipara	Small angle shades	Lepidoptera	SAMEA7701470	Male	ABDOMEN
Euproctis similis	Yellow-tail	Lepidoptera	SAMEA7519909	Male	THORAX | ABDOMEN
Eupsilia transversa	satellite moth	Lepidoptera	SAMEA8563699	Female	THORAX | ABDOMEN

Fabriciana adippe	High brown fritillary	Lepidoptera	SAMEA7523308	Female	ABDOMEN
Furcula furcula	Sallow kitten	Lepidoptera	SAMEA7746637	Male	THORAX
Glaucopsyche alexis	Green-underside blue	Lepidoptera	SAMEA7524616	Male	WHOLE ORGANISM
Griposia aprilina	Merveille du jour	Lepidoptera	SAMEA8603200	Female	THORAX
Gymnoscelis rufifasciata	Double-striped pug	Lepidoptera	SAMEA7519910	Female	WHOLE ORGANISM
Habrosyne pyritoides	Buff arches	Lepidoptera	SAMEA7701298	Male	ABDOMEN
Hamearis lucina	Duke of Burgundy	Lepidoptera	SAMEA7523117	Unknown	WHOLE ORGANISM
Hecatera dysodea	Small ranunculus	Lepidoptera	SAMEA7521514	Female	THORAX
Hedya salicella	White-backed Marble	Lepidoptera	SAMEA7520688	Male	WHOLE ORGANISM
Hemaris fuciformis	broad-bordered bee hawk-moth	Lepidoptera	SAMEA5248724	Unknown	WHOLE ORGANISM
Hesperia comma	Silver-spotted skipper	Lepidoptera	SAMEA7523119	Female	WHOLE ORGANISM
Hydraecia micacea	Rosy rustic	Lepidoptera	SAMEA8603188	Female	THORAX
Hydriomena furcata	July highflyer	Lepidoptera	SAMEA7701301	Male	ABDOMEN
Hylaea fasciaria	Barred red	Lepidoptera	SAMEA7520684	Male	HEAD | THORAX
Hypena proboscidalis	The snout	Lepidoptera	SAMEA7520188	Female	HEAD | THORAX
Idaea aversata	Riband Wave	Lepidoptera	SAMEA7519834	Male	WHOLE ORGANISM
Laothoe populi	Poplar Hawk-moth	Lepidoptera	SAMEA7520519	Female	ABDOMEN
Lasiommata megera	Wall brown	Lepidoptera	SAMEA7523153	Female	WHOLE ORGANISM
Laspeyria flexula	Beautiful hook-tip	Lepidoptera	SAMEA7519836	Male	WHOLE ORGANISM
Leptidea sinapis	Wood White	Lepidoptera	SAMEA7523467	Male	WHOLE ORGANISM
Limenitis camilla	White admiral	Lepidoptera	SAMEA7523310	Female	WHOLE ORGANISM
Lobophora halterata	The Seraphim	Lepidoptera	SAMEA7520514	Female	ABDOMEN
Luperina testacea	Flounced Rustic	Lepidoptera	SAMEA8534287	Male	THORAX
Lycaena phlaeas	Small copper	Lepidoptera	SAMEA7523293	Male	WHOLE ORGANISM
Lymantria monacha	Black arches	Lepidoptera	SAMEA7519912	Male	THORAX | ABDOMEN
Lysandra bellargus	Adonis Blue	Lepidoptera	SAMEA7523471	Female	WHOLE ORGANISM
Lysandra coridon	Chalkhill blue	Lepidoptera	SAMEA7523305	Male	WHOLE ORGANISM
Macaria notata	Peacock moth	Lepidoptera	SAMEA7746623	Male	ABDOMEN
Mamestra brassicae	Cabbage moth	Lepidoptera	SAMEA7524129	Male	MID_BODY
Maniola jurtina	Meadow brown	Lepidoptera	SAMEA7523158	Female	WHOLE ORGANISM
Marasmarcha lunaedactyla	Crescent plume	Lepidoptera	SAMEA7701293	Female	WHOLE ORGANISM

Meganola albula	Kent black arches	Lepidoptera	SAMEA7701294	Male	WHOLE ORGANISM
Melanargia galathea	Marbled white	Lepidoptera	SAMEA7523296	Female	WHOLE ORGANISM
Melitaea cinxia	Glanville Fritillary	Lepidoptera	SAMEA7523475	Male	WHOLE ORGANISM
Mellicta athalia	Heath fritillary	Lepidoptera	SAMEA7523312	Female	WHOLE ORGANISM
Mesoligia furuncula	Cloaked minor	Lepidoptera	SAMEA7701289	Female	WHOLE ORGANISM
Mimas tiliae	Lime Hawk-moth	Lepidoptera	SAMEA7520521	Male	ABDOMEN
Mythimna albipuncta	White-point	Lepidoptera	SAMEA8603191	Male	THORAX
Mythimna ferrago	The clay	Lepidoptera	SAMEA7701536	Female	ABDOMEN
Mythimna impura	Smoky wainscot	Lepidoptera	SAMEA7519913	Female	THORAX | ABDOMEN
Neocochylis molliculana	Ox-tongue Conch	Lepidoptera	SAMEA7746615	Unknown	WHOLE ORGANISM
Noctua comes	Lesser yellow underwing	Lepidoptera	SAMEA7701458	Unknown	ABDOMEN
Noctua fimbriata	Broad-bordered yellow underwing	Lepidoptera	SAMEA7519914	Female	THORAX | ABDOMEN
Noctua janthe	Lesser broad-boardered yellow underwing	Lepidoptera	SAMEA7701537	Male	ABDOMEN
Noctua pronuba	Large Yellow Underwing	Lepidoptera	SAMEA7519837	Female	WHOLE ORGANISM
Notocelia uddmanniana	Bramble shoot moth	Lepidoptera	SAMEA7519916	Male	WHOLE ORGANISM
Notodonta dromedarius	Iron prominent	Lepidoptera	SAMEA7520190	Male	HEAD | THORAX
Notodonta ziczac	Pebble prominent	Lepidoptera	SAMEA7746619	Male	THORAX
Nymphalis c-album	Comma	Lepidoptera	SAMEA7523165	Female	WHOLE ORGANISM
Nymphalis io	Peacock	Lepidoptera	SAMEA7523149	Male	WHOLE ORGANISM
Nymphalis polychloros	Large Tortoiseshell	Lepidoptera	SAMEA7523477	Female	THORAX
Nymphalis urticae	Small tortoiseshell	Lepidoptera	SAMEA7523286	Female	WHOLE ORGANISM
Ochlodes sylvanus	Large skipper	Lepidoptera	SAMEA7523138	Female	WHOLE ORGANISM
Ochropleura plecta	Flame shoulder	Lepidoptera	SAMEA7520524	Female	ABDOMEN
Omphaloscelis lunosa	Lunar underwing	Lepidoptera	SAMEA8603195	Female	THORAX
Operophtera brumata	Winter moth	Lepidoptera	SAMEA8563695	Male	THORAX | ABDOMEN
Opisthograptis luteolata	Brimstone moth	Lepidoptera	SAMEA7519838	Male	WHOLE ORGANISM
Orgyia antiqua	Rusty tussock moth	Lepidoptera	SAMEA7524390	Male	THORAX | ABDOMEN
Ostrinia nubilalis	European corn borer	Lepidoptera	SAMEA7701321	Unknown	WHOLE ORGANISM
Pammene fasciana	Acorn Piercer	Lepidoptera	SAMEA7701530	Male	WHOLE ORGANISM
Pandemis cinnamomeana	White-faced tortrix	Lepidoptera	SAMEA8603177	Male	WHOLE ORGANISM
Pandemis corylana	Chequered Fruit-tree Tortrix	Lepidoptera	SAMEA7701543	Unknown	WHOLE ORGANISM

Papilio machaon	common yellow swallowtail	Lepidoptera	SAMEA7523121	Female	WHOLE ORGANISM
Parapoynx stratiotata	Ringed china-mark	Lepidoptera	SAMEA7519920	Male	WHOLE ORGANISM
Pararge aegeria	Speckled wood	Lepidoptera	SAMEA7532732	Female	WHOLE ORGANISM
Peribatodes rhomboidaria	Willow beauty	Lepidoptera	SAMEA7701524	Male	ABDOMEN
Perizoma alchemillatum	Small rivulet	Lepidoptera	SAMEA7701545	Unknown	WHOLE ORGANISM
Perizoma flavofasciatum	Sandy carpet	Lepidoptera	SAMEA7701445	Unknown	WHOLE ORGANISM
Phalera bucephala	Buff-tip	Lepidoptera	SAMEA7519921	Female	THORAX | ABDOMEN
Pheosia gnoma	Lesser swallow prominent	Lepidoptera	SAMEA7520513	Male	ABDOMEN
Pheosia tremula	Swallow prominent	Lepidoptera	SAMEA7520523	Male	THORAX | ABDOMEN
Philereme vetulata	Brown scallop	Lepidoptera	SAMEA7701300	Female	ABDOMEN
Phlogophora meticulosa	Angle shades	Lepidoptera	SAMEA7520192	Female	HEAD | THORAX
Photedes minima	Small dotted buff	Lepidoptera	SAMEA7701533	Unknown	WHOLE ORGANISM
Phragmatobia fuliginosa	Ruby tiger moth	Lepidoptera	SAMEA7701498	Male	ABDOMEN
Pieris brassicae	Large white	Lepidoptera	SAMEA7532735	Female	WHOLE ORGANISM
Pieris napi	Green veined white	Lepidoptera	SAMEA7523140	Male	WHOLE ORGANISM
Pieris rapae	Small white	Lepidoptera	SAMEA7523164	Female	WHOLE ORGANISM
Plebejus argus	Silver-studded blue	Lepidoptera	SAMEA7523294	Male	WHOLE ORGANISM
Plutella xylostella	Diamondback moth	Lepidoptera	SAMEA7520369	Male	WHOLE ORGANISM
Polyommatus icarus	Common blue	Lepidoptera	SAMEA7523143	Male	WHOLE ORGANISM
Psoricoptera gibbosella	Humped Groundling	Lepidoptera	SAMEA7746612	Unknown	WHOLE ORGANISM
Ptilodon capucinus	Coxcomb prominent	Lepidoptera	SAMEA7746620	Male	THORAX
Ptycholomoides aeriferana	Yellow Larch Tortrix	Lepidoptera	SAMEA7701538	Unknown	WHOLE ORGANISM
Pyrgus malvae	Grizzled skipper	Lepidoptera	SAMEA7523277	Male	WHOLE ORGANISM
Rhopobota naevana	Holly tortrix	Lepidoptera	SAMEA7701517	Unknown	WHOLE ORGANISM
Schrankia costaestrigalis	Pinion-streaked snout	Lepidoptera	SAMEA7520193	Male	WHOLE ORGANISM
Scotopteryx chenopodiata	Shaded broad-bar	Lepidoptera	SAMEA7701561	Unknown	WHOLE ORGANISM
Selenia dentaria	Early thorn	Lepidoptera	SAMEA7701559	Male	ABDOMEN
Sesia apiformis	Hornet moth	Lepidoptera	SAMEA7701281	Male	HEAD | THORAX
Sphinx pinastri	Pine hawkmoth	Lepidoptera	SAMEA7701449	Unknown	ABDOMEN
Spilarctia lutea	Buff ermine	Lepidoptera	SAMEA7631557	Female	ABDOMEN
Spilosoma lubricipeda	White ermine	Lepidoptera	SAMEA7520525	Male	ABDOMEN

Synanthedon vespiformis	Yellow-legged clearwing	Lepidoptera	SAMEA7701494	Male	ABDOMEN
Thumatha senex	Round-winged muslin	Lepidoptera	SAMEA7701482	Unknown	WHOLE ORGANISM
Thyatira batis	Peach blossom	Lepidoptera	SAMEA7519923	Male	THORAX | ABDOMEN
Thymelicus lineola	Essex skipper	Lepidoptera	SAMEA7523301	Female	WHOLE ORGANISM
Thymelicus sylvestris	Small skipper	Lepidoptera	SAMEA7523279	Male	WHOLE ORGANISM
Tinea semifulvella	Fulvous Clothes Moth	Lepidoptera	SAMEA7520371	Male	WHOLE ORGANISM
Tinea trinotella	Bird's-nest moth	Lepidoptera	SAMEA7519924	Male	WHOLE ORGANISM
Udea prunalis	Dusky Pearl	Lepidoptera	SAMEA7701501	Unknown	WHOLE ORGANISM
Vanessa atalanta	Red admiral	Lepidoptera	SAMEA7523145	Female	WHOLE ORGANISM
Vanessa cardui	Painted lady	Lepidoptera	SAMEA7523147	Female	WHOLE ORGANISM
Watsonalla binaria	Oak hook-tip	Lepidoptera	SAMEA7746618	Female	ABDOMEN
Xanthorhoe fluctuata	Garden carpet	Lepidoptera	SAMEA7701525	Unknown	WHOLE ORGANISM
Xestia c-nigrum	Setaceous Hebrew character	Lepidoptera	SAMEA8239458	Male	THORAX
Xestia xanthographa	Square-spot rustic	Lepidoptera	SAMEA7520195	Female	HEAD | THORAX
Yponomeuta plumbellus	Black-tipped Ermine	Lepidoptera	SAMEA7746626	Unknown	WHOLE ORGANISM
Yponomeuta sedellus	Grey Ermine	Lepidoptera	SAMEA7746622	Male	WHOLE ORGANISM
Ypsolopha scabrella	Wainscot Smudge	Lepidoptera	SAMEA7701504	Male	WHOLE ORGANISM
Ypsolopha sequella	Pied smudge	Lepidoptera	SAMEA7519929	Male	WHOLE ORGANISM
Zeiraphera isertana	Cock's-head Bell	Lepidoptera	SAMEA7701465	Unknown	WHOLE ORGANISM
Zeuzera pyrina	Leopard moth	Lepidoptera	SAMEA7701286	Male	ABDOMEN
Zygaena filipendulae	6-spot burnet	Lepidoptera	SAMEA7519846	Female	WHOLE ORGANISM
Apoderus coryli	Hazel leaf-roller	Coleoptera	SAMEA7520690	Male	WHOLE ORGANISM
Cantharis rustica		Coleoptera	SAMEA7524272	Male	HEAD | THORAX
Podabrus alpinus		Coleoptera	SAMEA7520644	Female	WHOLE ORGANISM
Rhagonycha fulva	Common red soldier beetle	Coleoptera	SAMEA7520319	Female	WHOLE ORGANISM
Dromius quadrimaculatus		Coleoptera	SAMEA7520206	Unknown	WHOLE ORGANISM
Nebria brevicollis		Coleoptera	SAMEA7520209	Female	HEAD | THORAX
Nebria salina		Coleoptera	SAMEA7524273	Female	THORAX
Ophonus ardosiacus		Coleoptera	SAMEA7746467	Female	ABDOMEN
Pterostichus madidus	Black clock beetle	Coleoptera	SAMEA7520318	Male	HEAD | THORAX
Adalia bipunctata	two-spotted ladybird beetle	Coleoptera	SAMEA9089055	Male	WHOLE ORGANISM

Chilocorus renipustulatus	kidney-spot ladybird beetle	Coleoptera	SAMEA7520200	Unknown	WHOLE ORGANISM
Coccinella septempunctata	Seven-spotted ladybird	Coleoptera	SAMEA7520205	Female	WHOLE ORGANISM
Harmonia axyridis	Harlequin ladybird	Coleoptera	SAMEA7520208	Female	WHOLE ORGANISM
Hippodamia variegata	Adonis ladybird	Coleoptera	SAMEA7849270	Unknown	WHOLE ORGANISM
Propylea quattuordecimpunctata	14-spot ladybird	Coleoptera	SAMEA7520316	Unknown	WHOLE ORGANISM
Rhinocyllus conicus		Coleoptera	SAMEA7701490	Unknown	WHOLE ORGANISM
Agrypnus murinus		Coleoptera	SAMEA7701277	Male	ABDOMEN
Melolontha melolontha	cockchafer	Coleoptera	SAMEA7524378	Male	THORAX
Malachius bipustulatus	Common malachite beetle	Coleoptera	SAMEA7520537	Female	WHOLE ORGANISM
Oedemera lurida		Coleoptera	SAMEA7520207	Unknown	WHOLE ORGANISM
Pyrochroa serraticornis	Red-headed cardinal beetle	Coleoptera	SAMEA7524259	Male	THORAX
Phosphuga atrata	Black snail beetle	Coleoptera	SAMEA7520321	Unknown	HEAD | THORAX
Ocypus olens	Devil's coach horse	Coleoptera	SAMEA7520211	Female	THORAX | ABDOMEN
Philonthus cognatus		Coleoptera	SAMEA8603235	Male	ABDOMEN
Acrocera orbiculus	Top-horned Hunchback	Diptera	SAMEA7701562	Unknown	WHOLE ORGANISM
Delia platura	Bean seed fly	Diptera	SAMEA7746747	Unknown	WHOLE ORGANISM
Machimus atricapillus		Diptera	SAMEA7849389	Male	ABDOMEN
Bombylius discolor	dotted bee fly	Diptera	SAMEA7524252	Female	THORAX
Bombylius major	dark edged bee fly	Diptera	SAMEA7524251	Male	THORAX
Bellardia pandia	Bisetose Emerald-bottle	Diptera	SAMEA7746779	Female	WHOLE ORGANISM
Lucilia richardsi		Diptera	SAMEA8603134	Unknown	THORAX
Pollenia angustigena	Narrow-cheeked Clusterfly	Diptera	SAMEA7746597	Female	THORAX
Protocalliphora azurea	Bird blowfly	Diptera	SAMEA7746778	Male	THORAX
Clusia tigrina		Diptera	SAMEA7701567	Male	WHOLE ORGANISM
Physocephala rufipes	Waisted Beegrabber	Diptera	SAMEA7746469	Unknown	THORAX
Sicus ferrugineus	Ferruginous Bee-grabber	Diptera	SAMEA7520692	Male	WHOLE ORGANISM
Thecophora atra		Diptera	SAMEA7849382	Male	HEAD | THORAX
Tipula paludosa	European crane fly	Diptera	SAMEA7520335	Female	HEAD | THORAX
Empis livida		Diptera	SAMEA8603147	Unknown	THORAX
Callomyia amoena		Diptera	SAMEA9066034	Female	WHOLE ORGANISM
Stomorhina lunata	Locust Blowfly	Diptera	SAMEA7849406	Female	THORAX

Tachina fera		Diptera	SAMEA7520333	Female	HEAD | THORAX
Sarcophaga caerulescens		Diptera	SAMEA7746589	Male	THORAX
Sarcophaga crassimargo		Diptera	SAMEA7746602	Male	WHOLE ORGANISM
Sarcophaga rosellei		Diptera	SAMEA7746603	Male	THORAX
Sarcophaga variegata		Diptera	SAMEA8603132	Male	THORAX
Scathophaga stercoraria	yellow dung fly	Diptera	SAMEA7520161	Male	HEAD | THORAX
Coremacera marginata		Diptera	SAMEA7521524	Female	THORAX
Baccha elongata	Gossamer Hoverfly	Diptera	SAMEA7520030	Female	WHOLE ORGANISM
Cheilosia pagana	Parsley Cheilosia	Diptera	SAMEA7746768	Female	WHOLE ORGANISM
Cheilosia soror	Red-horned Truffle Cheilosia	Diptera	SAMEA7520031	Female	WHOLE ORGANISM
Cheilosia vulpina	Large Burdock Cheilosia	Diptera	SAMEA7746587	Female	THORAX
Chrysotoxum bicinctum	Two-banded wasp hoverfly	Diptera	SAMEA7520032	Female	HEAD | THORAX
Chrysotoxum verralli	Verrall's wasp hoverfly	Diptera	SAMEA7520033	Female	THORAX
Criorhina berberina	Dimorphic Bear Hoverfly	Diptera	SAMEA7701563	Female	ABDOMEN
Epistrophe grossulariae	Broad-banded Epistrophe	Diptera	SAMEA8603153	Female	ABDOMEN
Episyrphus balteatus	Marmalade hoverfly	Diptera	SAMEA7520035	Unknown	HEAD | THORAX
Eristalinus sepulchralis	Small Spotty-eyed Dronefly	Diptera	SAMEA7746477	Female	THORAX
Eristalis arbustorum	Plane-faced dronefly	Diptera	SAMEA7520036	Female	HEAD | THORAX
Eristalis horticola		Diptera	SAMEA7702268	Female	WHOLE ORGANISM
Eristalis pertinax	Tapered Dronefly	Diptera	SAMEA7520039	Male	HEAD | THORAX
Eristalis tenax	Common Dronefly	Diptera	SAMEA7520042	Female	HEAD | THORAX
Eupeodes corollae		Diptera	SAMEA7524255	Female	THORAX | ABDOMEN
Eupeodes latifasciatus	Meadow Field Syrph	Diptera	SAMEA7746776	Female	THORAX
Leucozona laternaria	Dark-saddled Leucozona	Diptera	SAMEA8603164	Female	THORAX
Melanostoma mellinum	Dumpy grass hoverfly	Diptera	SAMEA7520051	Male	HEAD | THORAX
Melanostoma scalare	Slender grass hoverfly	Diptera	SAMEA7520053	Male	HEAD | THORAX
Myathropa florea	Batman hoverfly	Diptera	SAMEA7520156	Male	HEAD | THORAX
Platycheirus albimanus	White-footed hoverfly	Diptera	SAMEA7520157	Female	HEAD | THORAX
Rhingia campestris	Common Snout-hoverfly	Diptera	SAMEA7520159	Male	ABDOMEN
Scaeva pyrastri	Pied hoverfly	Diptera	SAMEA7520160	Female	HEAD | THORAX
Sphaerophoria taeniata		Diptera	SAMEA7746606	Male	THORAX | ABDOMEN

Volucella inanis	Lesser hornet hoverfly	Diptera	SAMEA7520171	Female	HEAD | THORAX
Volucella inflata	Cossus Hoverfly	Diptera	SAMEA7701275	Male	ABDOMEN
Xanthogramma pedissequum	Superb ant-hill hoverfly	Diptera	SAMEA7520951	Male	THORAX
Xylota sylvarum	Golden-tailed hoverfly	Diptera	SAMEA7520173	Male	HEAD | THORAX
Cistogaster globosa		Diptera	SAMEA7746478	Male	WHOLE ORGANISM
Gymnosoma rotundatum		Diptera	SAMEA7849381	Male	THORAX
Nowickia ferox		Diptera	SAMEA7746479	Female	THORAX
Thecocarcelia acutangulata		Diptera	SAMEA7746598	Female	THORAX
Anomoia purmunda	Hawthorn fruitfly	Diptera	SAMEA7520325	Female	WHOLE ORGANISM
Merzomyia westermanni		Diptera	SAMEA7746463	Unknown	THORAX | ABDOMEN
Terellia serratulae		Diptera	SAMEA7520334	Female	WHOLE ORGANISM
Bibio marci	St Mark's Fly	Diptera	SAMEA7524263	Male	ABDOMEN
Nephrotoma flavescens	Tiger Cranefly	Diptera	SAMEA7520954	Male	WHOLE ORGANISM
Cloeon dipterum	Pond Olive	Ephemeroptera	SAMEA7520803	Unknown	WHOLE ORGANISM
Ecdyonurus torrentis	Large brook dun	Ephemeroptera	SAMEA7520824	Unknown	THORAX | POSTERIOR_BODY
Rhithrogena germanica		Ephemeroptera	SAMEA9065858	Unknown	ANTERIOR_BODY
Acanthosoma haemorrhoidale	Hawthorn shieldbug	Hemiptera	SAMEA8563710	Male	ABDOMEN
Gonocerus acuteangulatus	Box Bug	Hemiptera	SAMEA7524254	Unknown	THORAX | ABDOMEN
Pantilius tunicatus		Hemiptera	SAMEA7520359	Unknown	HEAD | THORAX
Himacerus mirmicoides	Ant damselbug	Hemiptera	SAMEA7520349	Unknown	WHOLE ORGANISM
Notonecta glauca	Backswimmer / Water Boatman	Hemiptera	SAMEA7520812	Unknown	WHOLE ORGANISM
Icerya purchasi	cottony cushion scale	Hemiptera	SAMEA7523480	Female	WHOLE ORGANISM
Aelia acuminata	Bishop's mitre shieldbug	Hemiptera	SAMEA7520338	Male	WHOLE ORGANISM
Eurydema oleracea	Brassica shieldbug	Hemiptera	SAMEA7701485	Unknown	WHOLE ORGANISM
Planococcus citri	citrus mealybug	Hemiptera	SAMEA7523510	Female	WHOLE ORGANISM
Andrena dorsata	Short-fringed Mining Bee	Hymenoptera	SAMEA7746464	Female	THORAX
Andrena haemorrhoa	Red-tailed mining bee	Hymenoptera	SAMEA7520535	Female	ABDOMEN
Andrena hattorfiana	Large scabious mining bee	Hymenoptera	SAMEA7746468	Female	THORAX
Bombus campestris	Field cuckoo-bee	Hymenoptera	SAMEA7520482	Male	HEAD | THORAX
Bombus hortorum	Garden bumblebee	Hymenoptera	SAMEA7520483	Female	HEAD | THORAX
Bombus hypnorum	Tree bumblebee	Hymenoptera	SAMEA7520655	Male	HEAD | THORAX

Bombus pascuorum	Common carder bee	Hymenoptera	SAMEA7520484	Female	HEAD | THORAX
Bombus pratorum	Early bumblebee	Hymenoptera	SAMEA7520485	Female	HEAD | THORAX
Bombus sylvestris	Forest cuckoo bee	Hymenoptera	SAMEA7520657	Male	HEAD | THORAX
Bombus terrestris	buff-tailed bumblebee	Hymenoptera	SAMEA7520487	Female	HEAD | THORAX
Nomada fabriciana	Fabricius' Nomad Bee	Hymenoptera	SAMEA7520701	Female	WHOLE ORGANISM
Hylaeus communis	Common Yellow-face Bee	Hymenoptera	SAMEA7746754	Female	WHOLE ORGANISM
Cerceris rybyensis	Ornate Tailed Digger Wasp	Hymenoptera	SAMEA7701329	Female	ABDOMEN
Ectemnius continuus		Hymenoptera	SAMEA7520490	Female	HEAD | THORAX
Ectemnius lituratus		Hymenoptera	SAMEA7520491	Female	HEAD | THORAX
Mimumesa dahlbomi		Hymenoptera	SAMEA8603165	Male	WHOLE ORGANISM
Nysson spinosus	Large Spurred Digger Wasp	Hymenoptera	SAMEA7520702	Female	HEAD | THORAX
Pemphredon lugubris	Mournful Wasp	Hymenoptera	SAMEA8603139	Unknown	THORAX
Trypoxylon clavicerum	Club Horned Wood Borer Wasp	Hymenoptera	SAMEA7701565	Female	WHOLE ORGANISM
Myrmica sabuleti		Hymenoptera	SAMEA7520497	Female	WHOLE ORGANISM
Lasioglossum calceatum	Common furrow bee	Hymenoptera	SAMEA7849393	Unknown	WHOLE ORGANISM
Lasioglossum lativentre	Furry-claspered furrow bee	Hymenoptera	SAMEA7746765	Male	WHOLE ORGANISM
Lasioglossum leucozonium	White-zoned furrow bee	Hymenoptera	SAMEA7746759	Male	WHOLE ORGANISM
Lasioglossum malachurum	Sharp-collared furrow bee	Hymenoptera	SAMEA7746751	Female	WHOLE ORGANISM
Lasioglossum morio	Common green furrow bee	Hymenoptera	SAMEA7746456	Male	WHOLE ORGANISM
Lasioglossum pauxillum	Base-banded furrow bee	Hymenoptera	SAMEA7520494	Female	WHOLE ORGANISM
Seladonia tumulorum	Bronze furrow bee	Hymenoptera	SAMEA7746445	Male	WHOLE ORGANISM
Sphecodes ephippius	Bare-saddled blood bee	Hymenoptera	SAMEA7746758	Male	WHOLE ORGANISM
Sphecodes monilicornis	Box-headed blood bee	Hymenoptera	SAMEA7746755	Male	THORAX
Amblyteles armatorius		Hymenoptera	SAMEA7520946	Male	THORAX | ABDOMEN
Buathra laborator		Hymenoptera	SAMEA8534297	Unknown	THORAX
Ichneumon xanthorius		Hymenoptera	SAMEA7746465	Female	THORAX
Ophion luteus		Hymenoptera	SAMEA8534284	Female	ABDOMEN
Scambus nigricans		Hymenoptera	SAMEA7849231	Female	WHOLE ORGANISM
Megachile ligniseca	Wood-carving leaf-cutter bee	Hymenoptera	SAMEA7520495	Female	WHOLE ORGANISM
Megachile willughbiella	Willughby's leaf-cutter bee	Hymenoptera	SAMEA7520496	Female	WHOLE ORGANISM
Macropis europaea	Yellow loosestrife Bee	Hymenoptera	SAMEA7746440	Male	THORAX

Anoplius nigerrimus		Hymenoptera	SAMEA7746764	Unknown	THORAX
Evagetes crassicornis		Hymenoptera	SAMEA7746601	Unknown	WHOLE ORGANISM
Athalia circularis		Hymenoptera	SAMEA7746777	Unknown	WHOLE ORGANISM
Athalia cordata		Hymenoptera	SAMEA7746763	Unknown	WHOLE ORGANISM
Athalia rosae	Coleseed sawfly	Hymenoptera	SAMEA7520481	Unknown	ABDOMEN
Tenthredo livida		Hymenoptera	SAMEA7520699	Unknown	HEAD | THORAX
Tenthredo notha		Hymenoptera	SAMEA7746761	Unknown	THORAX
Tiphia femorata	Beetle killing wasp	Hymenoptera	SAMEA7520499	Female	WHOLE ORGANISM
Ancistrocerus nigricornis	Early mason-wasp	Hymenoptera	SAMEA7746762	Female	THORAX
Dolichovespula media	Median wasp	Hymenoptera	SAMEA7520488	Female	HEAD | THORAX
Dolichovespula saxonica	Saxon wasp	Hymenoptera	SAMEA7520489	Male	HEAD | THORAX
Dolichovespula sylvestris	Tree wasp	Hymenoptera	SAMEA7746475	Male	THORAX
Vespa crabro	European hornet	Hymenoptera	SAMEA7520500	Female	THORAX
Vespula germanica	German wasp	Hymenoptera	SAMEA7520501	Female	HEAD | THORAX
Vespula vulgaris	Common wasp	Hymenoptera	SAMEA7520502	Female	HEAD | THORAX
Chrysoperla carnea	Common green lacewing	Neuroptera	SAMEA7520372	Female	WHOLE ORGANISM
Ischnura elegans	Blue-tailed damselfly	Odonata	SAMEA7521125	Female	WHOLE ORGANISM
Sympetrum striolatum	Common darter	Odonata	SAMEA7520376	Female	HEAD | THORAX
Chorthippus brunneus	Field grasshopper	Orthoptera	SAMEA7520377	Unknown	HEAD
Chorthippus parallelus	Meadow grasshopper	Orthoptera	SAMEA7520378	Unknown	HEAD
Teleogryllus oceanicus	Black field cricket	Orthoptera	SAMEA8023467	Female	WHOLE ORGANISM
Meconema thalassinum	Oak bush-cricket	Orthoptera	SAMEA7520379	Male	THORAX | ABDOMEN
Leuctra nigra		Plecoptera	SAMEA7521360	Male	WHOLE ORGANISM
Nemoura dubitans		Plecoptera	SAMEA9065873	Female	WHOLE ORGANISM
Nemurella pictetii		Plecoptera	SAMEA7520996	Male	WHOLE ORGANISM
Glyphotaelius pellucidus		Trichoptera	SAMEA7520965	Male	WHOLE ORGANISM
Limnephilus lunatus		Trichoptera	SAMEA7521206	Female	WHOLE ORGANISM
Limnephilus marmoratus		Trichoptera	SAMEA7520990	Male	WHOLE ORGANISM
Limnephilus rhombicus		Trichoptera	SAMEA7849396	Male	ABDOMEN
Polycentropus irroratus		Trichoptera	SAMEA9065840	Unknown	ANTERIOR_BODY
