# Supplementary material for: Phylogenomic analysis of Wolbachia genomes from the Darwin Tree of Life biodiversity genomics project
Source: PLoS Biol. 2023 Jan 23;21(1):e3001972. doi: 10.1371/journal.pbio.3001972 (PMC9894559; doi:10.1371/journal.pbio.3001972)
Supplement: S2 Table — (PDF) [file pbio.3001972.s003.pdf]

S2 Table. Overview of all detected *Wolbachia* infections

Scientific name host	Taxonomic order host	Wolbachia supergroup	Circular / linear	Coverage	Number of contigs	Genome size (bp)	BUSCO completeness	Assembly method	Estimated number of Wolbachia genomes	Estimated number of Wolbachia genomes per host	Accesion number
Agriphila straminella	Lepidoptera	B	circular	42	1	1856314	C:99.4%[S:98.9%,D:0.5%],F:0.0%,M:0.6%,n:364	hifiasm	2	2	GCA_947250755
Agriphila tristella	Lepidoptera	B	linear	135	2	1312870	C:99.8%[S:99.5%,D:0.3%],F:0.0%,M:0.2%,n:364	flye	9	9	GCA_947179465
Aporia crataegi	Lepidoptera	B	linear	303	1	1310109	C:99.7%[S:99.7%,D:0.0%],F:0.0%,M:0.3%,n:364	flye	6	6	GCA_947250485
Apotomis betuletana	Lepidoptera	B	linear	28	1	1635810	C:99.7%[S:98.9%,D:0.8%],F:0.0%,M:0.3%,n:364	hifiasm	2	2	GCA_947250475
Apotomis turbidana	Lepidoptera	B	circular	51	1	1525756	C:99.4%[S:98.9%,D:0.5%],F:0.3%,M:0.3%,n:364	hifiasm	3	3	GCA_947251835
Archips podanus	Lepidoptera	B	circular	50	1	1806407	C:99.4%[S:98.9%,D:0.5%],F:0.3%,M:0.3%,n:364	hifiasm	4	4	GCA_947250705
Aricia agestis	Lepidoptera	B	linear	115	2	1562496	C:99.7%[S:98.9%,D:0.8%],F:0.0%,M:0.3%,n:364	hifiasm	4	4	GCA_947179385
Autographa gamma	Lepidoptera	Not well assembled - unclear number of Wolbachia strains									
Calamotropha paludella	Lepidoptera	A	circular	78	1	1594318	C:99.5%[S:99.2%,D:0.3%],F:0.0%,M:0.5%,n:364	flye	4	9	GCA_947251935
		A	circular	85	1	1378301	C:99.5%[S:99.2%,D:0.3%],F:0.0%,M:0.5%,n:364	flye	5		GCA_947251525
Campaea margaritaria	Lepidoptera	B	circular	456	1	1507682	C:99.7%[S:99.2%,D:0.5%],F:0.0%,M:0.3%,n:364	flye	19	19	GCA_947250595
Camptogramma bilineatum	Lepidoptera	B	circular	41	1	1668206	C:99.2%[S:98.4%,D:0.8%],F:0.0%,M:0.8%,n:364	hifiasm	2	2	GCA_947251825
Carcina quercana	Lepidoptera	B	linear	18	4	1572160	C:99.8%[S:99.5%,D:0.3%],F:0.0%,M:0.2%,n:364	flye	1	1	GCA_947179375
Catoptria pinella	Lepidoptera	B	circular	83	1	1667357	C:99.8%[S:99.5%,D:0.3%],F:0.0%,M:0.2%,n:364	hifiasm	3	3	GCA_947250515
Celastrina argiolus	Lepidoptera	B	circular	140	1	1329046	C:99.7%[S:99.7%,D:0.0%],F:0.0%,M:0.3%,n:364	hifiasm	9	9	GCA_947251805
Chrysoteuchia culmella	Lepidoptera	Not well assembled - unclear number of Wolbachia strains									
Colias croceus	Lepidoptera	B	circular	158	1	1396841	C:99.8%[S:99.5%,D:0.3%],F:0.0%,M:0.2%,n:364	hifiasm	5	5	GCA_947250525
Cydia splendana	Lepidoptera	A	linear	154	2	1446399	C:98.9%[S:98.6%,D:0.3%],F:0.3%,M:0.8%,n:364	flye	9	9	GCA_947179475
Emmelina monodactyla	Lepidoptera	B	circular	177	1	1385743	C:99.7%[S:99.7%,D:0.0%],F:0.0%,M:0.3%,n:364	hifiasm	9	9	GCA_947251605
Endotricha flammealis	Lepidoptera	A	circular	18	1	1691857	C:99.5%[S:99.2%,D:0.3%],F:0.0%,M:0.5%,n:364	hifiasm	2	4	GCA_947250805
		B	circular	18	1	1747616	C:99.7%[S:99.2%,D:0.5%],F:0.0%,M:0.3%,n:364	hifiasm	2		GCA_947251735
Epagoge grotiana	Lepidoptera	A	circular	315	1	1431999	C:99.2%[S:98.9%,D:0.3%],F:0.3%,M:0.5%,n:364	hifiasm	13	19	GCA_947251745
		A	circular	135	1	1781589	C:98.6%[S:98.6%,D:0.0%],F:0.5%,M:0.9%,n:364	hifiasm	6		GCA_947251775
Epirrhoe alternata	Lepidoptera	A	circular	608	1	1197406	C:98.9%[S:98.9%,D:0.0%],F:0.0%,M:1.1%,n:364	hifiasm	23	23	GCA_947251475
Erebia ligea	Lepidoptera	B	linear	33	4	1520330	C:98.9%[S:98.4%,D:0.5%],F:0.0%,M:1.1%,n:364	flye	2	2	GCA_947179575
Erynnis tages	Lepidoptera	B	linear	113	1	1532960	C:99.8%[S:99.5%,D:0.3%],F:0.0%,M:0.2%,n:364	hifiasm	8	8	GCA_947250545
Eucosma cana	Lepidoptera	B	circular	133	1	1407592	C:98.9%[S:98.4%,D:0.5%],F:0.5%,M:0.6%,n:364	hifiasm	6	6	GCA_947250645
Euphydryas aurinia	Lepidoptera	B	circular	209	1	1802478	C:99.7%[S:98.6%,D:1.1%],F:0.0%,M:0.3%,n:364	hifiasm	12	12	GCA_947250535
Eupithecia tripunctaria	Lepidoptera	A	circular	31	1	1478720	C:99.4%[S:98.9%,D:0.5%],F:0.0%,M:0.6%,n:364	hifiasm	2	2	GCA_947251595
Hamearis lucina	Lepidoptera	Not well assembled - unclear number of Wolbachia strains									
Hedya salicella	Lepidoptera	Not well assembled - unclear number of Wolbachia strains + not enough coverage									
Hesperia comma	Lepidoptera	Not well assembled - not enough coverage									
Hylaea fasciaria	Lepidoptera	B	circular	772	1	1307822	C:99.7%[S:99.7%,D:0.0%],F:0.0%,M:0.3%,n:364	flye	22	22	GCA_947251975
Hypena proboscidalis	Lepidoptera	Not well assembled - high number of detected SNPs with 10x data									
Idaea aversata	Lepidoptera	B	linear	135	2	1828659	C:99.4%[S:98.9%,D:0.5%],F:0.0%,M:0.6%,n:364	hifiasm	5	5	GCA_947179535
Laothoe populi	Lepidoptera	Not well assembled - unclear number of Wolbachia strains									
Leptidea sinapis	Lepidoptera	B	linear	77	4	1438040	C:99.5%[S:99.2%,D:0.3%],F:0.3%,M:0.2%,n:364	flye	4	4	GCA_947179455

Lycaena phlaeas	Lepidoptera	B	circular	24	1	1312316	C:99.7%[S:99.7%,D:0.0%],F:0.0%,M:0.3%,n:364	hifiasm-meta	1	1	GCA_947251875
Melanargia galathea	Lepidoptera	B	circular	94	1	1550695	C:99.7%[S:99.2%,D:0.5%],F:0.0%,M:0.3%,n:364	flye	7	7	/
		B	circular	161	1	1381694	C:99.7%[S:99.7%,D:0.0%],F:0.0%,M:0.3%,n:364	hifiasm	8	10	GCA_947250815
Nymphalis c-album	Lepidoptera	B	circular	43	1	1236246	C:99.7%[S:99.7%,D:0.0%],F:0.0%,M:0.3%,n:364	hifiasm	2		GCA_947250465
Ochlodes sylvanus	Lepidoptera	B	linear	27	2	1713863	C:99.4%[S:98.9%,D:0.5%],F:0.0%,M:0.6%,n:364	flye	2	2	GCA_947179425
Opisthograptis luteolata	Lepidoptera	Not well assembled - unclear number of Wolbachia strains									
Orgyia antiqua	Lepidoptera	B	linear	258	1	1546986	C:99.7%[S:99.2%,D:0.5%],F:0.0%,M:0.3%,n:364	flye	10	10	/
Pammene fasciana	Lepidoptera	B	circular	103	1	1266982	C:99.7%[S:99.7%,D:0.0%],F:0.0%,M:0.3%,n:364	hifiasm	6	6	GCA_947251495
Pandemis cinnamomeana	Lepidoptera	B	linear	34	4	1779235	C:99.7%[S:99.2%,D:0.5%],F:0.0%,M:0.3%,n:364	flye	2	2	GCA_947179145
Pandemis corylana	Lepidoptera	B	circular	193	1	1510264	C:99.4%[S:98.6%,D:0.8%],F:0.3%,M:0.3%,n:364	flye	8	8	GCA_947250555
Parapoynx stratiotata	Lepidoptera	B	circular	14	1	1545403	C:99.7%[S:99.2%,D:0.5%],F:0.0%,M:0.3%,n:364	hifiasm	1	1	GCA_947250635
Pararge aegeria	Lepidoptera	B	circular	107	1	1332905	C:99.7%[S:99.7%,D:0.0%],F:0.0%,M:0.3%,n:364	hifiasm	5	5	GCA_947250725
		A	circular	62	1	1271809	C:99.2%[S:98.9%,D:0.3%],F:0.0%,M:0.8%,n:364	hifiasm	4	21	GCA_947250505
		B	linear	272	1	1358027	C:99.5%[S:99.5%,D:0.0%],F:0.3%,M:0.2%,n:364	hifiasm	16		GCA_947251625
Phalera bucephala	Lepidoptera	B	linear	9	1	1462983	C:99.2%[S:98.9%,D:0.3%],F:0.3%,M:0.5%,n:364	hifiasm	1		GCA_947251535
Pheosia gnoma	Lepidoptera	A	circular	393	1	1353731	C:99.5%[S:99.5%,D:0.0%],F:0.0%,M:0.5%,n:364	hifiasm	13	13	GCA_947251565
Pheosia tremula	Lepidoptera	B	circular	243	1	1268782	C:99.2%[S:99.2%,D:0.0%],F:0.5%,M:0.3%,n:364	hifiasm	7	7	GCA_947251655
Polyommatus icarus	Lepidoptera	B	linear	333	1	1574132	C:99.8%[S:99.5%,D:0.3%],F:0.0%,M:0.2%,n:364	hifiasm	19	19	GCA_947250745
Pyrgus malvae	Lepidoptera	B	circular	254	1	1544873	C:99.1%[S:98.6%,D:0.5%],F:0.5%,M:0.4%,n:364	flye	7	7	GCA_947250605
Rhopobota naevana	Lepidoptera	B	circular	13	1	1771993	C:99.5%[S:98.4%,D:1.1%],F:0.0%,M:0.5%,n:364	hifiasm	1	1	GCA_947250615
Thymelicus sylvestris	Lepidoptera	B	linear	960	1	1286255	C:99.7%[S:99.7%,D:0.0%],F:0.0%,M:0.3%,n:364	flye	48	48	GCA_947251505
Watsonalla binaria	Lepidoptera	B	circular	218	1	1651336	C:99.7%[S:99.2%,D:0.5%],F:0.0%,M:0.3%,n:364	hifiasm	6	6	GCA_947250655
Xestia c-nigrum	Lepidoptera	B	circular	106	1	1372378	C:99.7%[S:99.7%,D:0.0%],F:0.0%,M:0.3%,n:364	hifiasm	6	6	GCA_947250675
Yponomeuta plumbellus	Lepidoptera	A	linear	181	1	1418562	C:98.9%[S:98.9%,D:0.0%],F:0.5%,M:0.6%,n:364	hifiasm	12	12	GCA_947251575
Apoderus coryli	Coleoptera	A	circular	31	1	1535196	C:99.8%[S:99.5%,D:0.3%],F:0.0%,M:0.2%,n:364	flye	1	1	GCA_947251615
Nebria salina	Coleoptera	Not well assembled - very high coverage (>1000x)									
Rhinocyllus conicus	Coleoptera	A	circular	123	1	1703908	C:99.2%[S:98.4%,D:0.8%],F:0.3%,M:0.5%,n:364	flye	5	5	GCA_947250775
		A	circular	25	1	1811324	C:99.4%[S:98.9%,D:0.5%],F:0.0%,M:0.6%,n:364	hifiasm	2	4	GCA_947251755
Philonthus cognatus	Coleoptera	B	linear	25	2	1680410	C:99.8%[S:98.4%,D:1.4%],F:0.0%,M:0.2%,n:364	hifiasm	2		GCA_947179345
Acrocera orbiculus	Diptera	A	circular	121	1	1383045	C:98.6%[S:98.6%,D:0.0%],F:0.3%,M:1.1%,n:364	hifiasm	2	2	GCA_947250015
Bombylius major	Diptera	A	circular	173	1	1556587	C:99.4%[S:98.6%,D:0.8%],F:0.0%,M:0.6%,n:364	flye	5	5	GCA_947251895
		A	circular	117	1	1434286	C:99.2%[S:99.2%,D:0.0%],F:0.3%,M:0.5%,n:364	flye	6	21	GCA_947251425
Protocalliphora azurea	Diptera	B	circular	291	1	1550351	C:99.5%[S:99.2%,D:0.3%],F:0.3%,M:0.2%,n:364	flye	15		GCA_947251865
Sicus ferrugineus	Diptera	A	circular	119	1	1273941	C:99.5%[S:99.5%,D:0.0%],F:0.3%,M:0.2%,n:364	hifiasm	5	5	GCA_947251515
Thecophora atra	Diptera	Not well assembled - unclear number of Wolbachia strains									
Coremacera marginata	Diptera	A	circular	115	1	1387347	C:99.2%[S:99.2%,D:0.0%],F:0.3%,M:0.5%,n:364	hifiasm	9	9	GCA_947251955
Baccha elongata	Diptera	Not well assembled - unclear number of Wolbachia strains									
		A	circular	86	1	1368131	C:99.4%[S:98.9%,D:0.5%],F:0.0%,M:0.6%,n:364	hifiasm	4	9	GCA_947250625
Cheilosia soror	Diptera	A	linear	96	2	1493041	C:99.2%[S:99.2%,D:0.0%],F:0.0%,M:0.8%,n:364	hifiasm	5		GCA_947179435
Cheilosia vulpina	Diptera	Not well assembled - unclear number of Wolbachia strains									
Chrysotoxum verralli	Diptera	Not well assembled - unclear number of Wolbachia strains									
		A	circular	23	1	1483878	C:99.4%[S:98.6%,D:0.8%],F:0.3%,M:0.3%,n:364	flye	2	3	GCA_947251925
Epistrophe grossulariae	Diptera	A	circular	20	1	1367569	C:99.2%[S:98.9%,D:0.3%],F:0.3%,M:0.5%,n:364	flye	1		GCA_947251435

Episyrphus balteatus	Diptera	B	circular	80	1	1402289	C:99.2%[S:98.4%,D:0.8%],F:0.5%,M:0.3%,n:364	hifiasm	6	6	GCA_947250575
Eupeodes latifasciatus	Diptera	B	circular	406	1	1274269	C:99.7%[S:99.7%,D:0.0%],F:0.0%,M:0.3%,n:364	flye	30	30	GCA_947250695
Melanostoma mellinum	Diptera	B	circular	43	1	1875946	C:99.2%[S:98.4%,D:0.8%],F:0.0%,M:0.8%,n:364	flye	3	3	GCA_947251465
Platycheirus albimanus	Diptera	A	linear	12	6	1174338	C:98.9%[S:98.9%,D:0.0%],F:0.5%,M:0.6%,n:364	flye	0	1	GCA_947179415
		A	circular	37	1	1354583	C:99.1%[S:98.6%,D:0.5%],F:0.5%,M:0.4%,n:364	flye	2	23	GCA_947250735
Sphaerophoria taeniata	Diptera	B	linear	375	3	1627501	C:99.4%[S:98.6%,D:0.8%],F:0.3%,M:0.3%,n:364	flye	21		GCA_947179355
		A	circular	44	1	1502845	C:98.6%[S:97.0%,D:1.6%],F:0.5%,M:0.9%,n:364	hifiasm	3	6	GCA_947250665
Volucella inflata	Diptera	A	linear	42	2	1346192	C:99.5%[S:99.2%,D:0.3%],F:0.0%,M:0.5%,n:364	hifiasm	3		GCA_947179175
Gymnosoma rotundatum	Diptera	A	circular	367	1	1431527	C:98.9%[S:98.4%,D:0.5%],F:0.3%,M:0.8%,n:364	flye	24	24	GCA_947250765
Anomoia purmunda	Diptera	A	circular	177	1	1485792	C:99.7%[S:99.2%,D:0.5%],F:0.0%,M:0.3%,n:364	flye	10	10	GCA_947251545
Merzomyia westermanni	Diptera	A	circular	163	1	1295458	C:99.5%[S:99.2%,D:0.3%],F:0.0%,M:0.5%,n:364	hifiasm	10	10	GCA_947251675
Bibio marci	Diptera	A	circular	91	1	1355352	C:98.7%[S:98.4%,D:0.3%],F:0.3%,M:1.0%,n:364	flye	3	3	GCA_947251645
Himacerus mirmicoides	Hemiptera	Not well assembled - unclear number of Wolbachia strains									
Icerya purchasi	Hemiptera	A	circular	88	1	1372034	C:99.4%[S:98.9%,D:0.5%],F:0.0%,M:0.6%,n:364	hifiasm	6	6	GCA_947251635
		A	circular	31	1	1366845	C:99.2%[S:99.2%,D:0.0%],F:0.3%,M:0.5%,n:364	hifiasm	2	3	GCA_947251765
Andrena dorsata	Hymenoptera	A	circular	11	1	1533669	C:99.7%[S:99.2%,D:0.5%],F:0.0%,M:0.3%,n:364	hifiasm	1		GCA_947251685
Andrena haemorrhoa	Hymenoptera	A	circular	194	1	1494006	C:98.9%[S:98.6%,D:0.3%],F:0.5%,M:0.6%,n:364	hifiasm	5	5	GCA_947250565
Andrena hattorfiana	Hymenoptera	A	linear	7	3	1409226	C:95.6%[S:95.3%,D:0.3%],F:2.7%,M:1.7%,n:364	flye	0	1	GCA_947179565
Nomada fabriciana	Hymenoptera	A	circular	321	1	1438975	C:99.5%[S:99.2%,D:0.3%],F:0.0%,M:0.5%,n:364	flye	7	7	GCA_947250795
Hylaeus communis	Hymenoptera	A	circular	1030	1	1526906	C:99.1%[S:98.6%,D:0.5%],F:0.3%,M:0.6%,n:364	flye	31	31	GCA_947251915
Ectemnius continuus	Hymenoptera	A	circular	349	1	1299221	C:99.5%[S:99.2%,D:0.3%],F:0.0%,M:0.5%,n:364	flye	8	8	GCA_947251695
Trypoxylon clavicerum	Hymenoptera	A	circular	55	1	1287268	C:98.6%[S:98.1%,D:0.5%],F:0.3%,M:1.1%,n:364	flye	1	1	GCA_947251665
Myrmica sabuleti	Hymenoptera	A	linear	49	1	1403111	C:99.2%[S:99.2%,D:0.0%],F:0.3%,M:0.5%,n:364	hifiasm	2	2	/
Lasioglossum calceatum	Hymenoptera	A	circular	10	1	1271041	C:98.7%[S:98.4%,D:0.3%],F:0.5%,M:0.8%,n:364	flye	1	1	/
Lasioglossum lativentre	Hymenoptera	A	linear	69	1	1306351	C:99.5%[S:99.2%,D:0.3%],F:0.0%,M:0.5%,n:364	flye	4	4	GCA_947251945
Lasioglossum leucozonium	Hymenoptera	Not well assembled - high number of detected SNPs with 10x data									
		A	linear	359	2	1380835	C:98.9%[S:98.6%,D:0.3%],F:0.3%,M:0.8%,n:364	flye	21	28	GCA_947179495
Lasioglossum malachurum	Hymenoptera	A	linear	119	2	1363507	C:99.5%[S:99.5%,D:0.0%],F:0.0%,M:0.5%,n:364	flye	7		GCA_947179405
		A	circular	219	1	1282688	C:99.5%[S:99.2%,D:0.3%],F:0.0%,M:0.5%,n:364	hifiasm	13	22	GCA_947250585
		A	circular	72	1	1270694	C:99.2%[S:99.2%,D:0.0%],F:0.0%,M:0.8%,n:364	hifiasm	4		GCA_947251705
Lasioglossum morio	Hymenoptera	A	linear	79	1	1284395	C:99.5%[S:99.2%,D:0.3%],F:0.0%,M:0.5%,n:364	hifiasm	5		GCA_947250495
Seladonia tumulorum	Hymenoptera	Not well assembled - unclear number of Wolbachia strains									
Sphecodes ephippius	Hymenoptera	A	linear	320	2	1317045	C:99.1%[S:98.6%,D:0.5%],F:0.0%,M:0.9%,n:364	hifiasm	15	15	GCA_947179545
		A	circular	245	1	1359653	C:98.9%[S:98.9%,D:0.0%],F:0.5%,M:0.6%,n:364	flye	14	20	GCA_947250685
Sphecodes monilicornis	Hymenoptera	A	circular	107	1	1141923	C:99.5%[S:99.5%,D:0.0%],F:0.0%,M:0.5%,n:364	flye	6		GCA_947251965
Scambus nigricans	Hymenoptera	A	circular	148	1	1150903	C:98.9%[S:98.9%,D:0.0%],F:0.3%,M:0.8%,n:364	hifiasm	9	9	GCA_947251485
Macropis europaea	Hymenoptera	A	circular	74	1	1439111	C:99.7%[S:99.7%,D:0.0%],F:0.0%,M:0.3%,n:364	hifiasm	5	5	GCA_947251555
Anoplius nigerrimus	Hymenoptera	A	circular	13	1	1345812	C:98.3%[S:97.8%,D:0.5%],F:0.8%,M:0.9%,n:364	flye	1	1	GCA_947251795
Athalia cordata	Hymenoptera	B	circular	2302	1	1345842	C:99.5%[S:98.4%,D:1.1%],F:0.3%,M:0.2%,n:364	flye	47	47	GCA_947251845
Tiphia femorata	Hymenoptera	A	circular	824	1	1330166	C:98.1%[S:97.8%,D:0.3%],F:0.3%,M:1.6%,n:364	hifiasm	22	22	GCA_947251725
Ancistrocerus nigricornis	Hymenoptera	A	circular	549	1	1265149	C:99.5%[S:99.5%,D:0.0%],F:0.0%,M:0.5%,n:364	flye	16	16	GCA_947250785
Dolichovespula media	Hymenoptera	B	linear	479	3	1685384	C:99.1%[S:98.6%,D:0.5%],F:0.3%,M:0.6%,n:364	flye	13	13	GCA_947179505
Ischnura elegans	Odonata	B	circular	33	1	2192296	C:99.5%[S:98.4%,D:1.1%],F:0.0%,M:0.5%,n:364	hifiasm	3	3	GCA_947251585

Sympetrum striolatum	Odonata	A	linear	113	1	1516595	C:99.1%[S:98.6%,D:0.5%],F:0.3%,M:0.6%,n:364	hifiasm	6	6	GCA_947250715
Chorthippus brunneus	Orthoptera	B	linear	558	4	2013544	C:99.5%[S:99.2%,D:0.3%],F:0.0%,M:0.5%,n:364	flye	33	33	GCA_947179555
Chorthippus parallelus	Orthoptera	B	circular	260	1	1748465	C:99.5%[S:98.4%,D:1.1%],F:0.0%,M:0.5%,n:364	flye	20	20	GCA_947251815
