# Supplementary material for: Phylogenomic analysis of Wolbachia genomes from the Darwin Tree of Life biodiversity genomics project
Source: PLoS Biol. 2023 Jan 23;21(1):e3001972. doi: 10.1371/journal.pbio.3001972 (PMC9894559; doi:10.1371/journal.pbio.3001972)
Supplement: S3 Table — (PDF) [file pbio.3001972.s004.pdf]

S3 Table. *Wolbachia* reference genomes

Assembly identifier	Short name	Taxonomic order host	Wolbachia supergroup	Circular / linear	Numbers of scaffolds	Genome size (bp)	BUSCO completeness	Removed contigs
GCA_902636475.1	R_Col_DiaVir_A	Coleopta	A	linear	105	1269619	C:95.4%[S:95.1%,D:0.3%],F:2.5%,M:2.1%,n:364	CACPRN010000079.1 (Brevundimonas), CACPRN010000081.1 (Pythium), CACPRN010000092.1 (Stenotrophomonas), CACPRN010000095.1(Rhizobacter), CACPRN010000100.1 (Sphingopyxis), CACPRN010000101.1 (Singulisphaera), CACPRN010000102.1 (Chitinophaga), CACPRN010000103.1 (Bacillus), CACPRN010000105.1 (uncultured bacterium), CACPRN010000106.1 (Sphingobacterium), CACPRN010000107.1 (Bosea), CACPRN010000109.1 (Advenella), CACPRN010000111.1 (Devosia), CACPRN010000119.1 (Niabella)
GCF_017896245.1	R_Dip_AedAeg_A	Diptera	A	circular	1	1267786	C:99.5%[S:99.2%,D:0.3%],F:0.0%,M:0.5%,n:364	
GCF_018454455.1	R_Dip_CerCap_A	Diptera	A	linear	65	1239646	C:99.4%[S:98.9%,D:0.5%],F:0.0%,M:0.6%,n:364	
GCA_021609905.1	R_Dip_DelRad_A	Diptera	A	circular	1	1586584	C:98.9%[S:98.1%,D:0.8%],F:0.3%,M:0.8%,n:364	
GCF_008033215.1	R_Dip_DroAna_A	Diptera	A	circular	1	1401460	C:99.2%[S:99.2%,D:0.0%],F:0.3%,M:0.5%,n:364	
GCA_014129655.1	R_Dip_DroAra_A	Diptera	A	linear	10	1293235	C:99.5%[S:99.5%,D:0.0%],F:0.0%,M:0.5%,n:364	
GCF_017916175.1	R_Dip_DroAur_A	Diptera	A	linear	116	1319217	C:99.2%[S:99.2%,D:0.0%],F:0.0%,M:0.8%,n:364	
GCF_014129605.1	R_Dip_DroBai_A	Diptera	A	linear	26	1190958	C:97.5%[S:97.5%,D:0.0%],F:0.3%,M:2.2%,n:364	
GCF_014129645.1	R_Dip_DroBic_A	Diptera	A	linear	24	1182871	C:99.2%[S:99.2%,D:0.0%],F:0.0%,M:0.8%,n:364	
GCF_014129685.1	R_Dip_DroBif_A	Diptera	A	linear	17	1190237	C:99.2%[S:99.2%,D:0.0%],F:0.0%,M:0.8%,n:364	
GCF_014129615.1	R_Dip_DroBor_A	Diptera	A	linear	16	1214204	C:98.4%[S:98.4%,D:0.0%],F:0.0%,M:1.6%,n:364	
GCF_001758565.1	R_Dip_DroInc_A	Diptera	A	circular	1	1267840	C:95.6%[S:95.6%,D:0.0%],F:2.5%,M:1.9%,n:364	
GCF_016584355.1	R_Dip_DroMel_A	Diptera	A	circular	1	1330657	C:99.5%[S:99.2%,D:0.3%],F:0.0%,M:0.5%,n:364	
GCF_014129535.1	R_Dip_DroNeo_A	Diptera	A	linear	19	1355678	C:99.2%[S:99.2%,D:0.0%],F:0.0%,M:0.8%,n:364	
GCF_014107455.1	R_Dip_DroNik_A	Diptera	A	circular	1	1139744	C:99.7%[S:99.7%,D:0.0%],F:0.0%,M:0.3%,n:364	
GCF_014129565.1	R_Dip_DroOri_A	Diptera	A	linear	19	1363981	C:99.2%[S:98.9%,D:0.3%],F:0.0%,M:0.8%,n:364	
GCA_000742435.1	R_Dip_DroRec_A	Diptera	A	linear	43	1126656	C:99.2%[S:98.9%,D:0.3%],F:0.3%,M:0.5%,n:364	
GCF_018467135.1	R_Dip_DroSan_A	Diptera	A	circular	1	1409094	C:99.5%[S:99.5%,D:0.0%],F:0.0%,M:0.5%,n:364	
GCA_014354315.1	R_Dip_DroSec_A	Diptera	A	linear	83	1294885	C:99.4%[S:98.9%,D:0.5%],F:0.0%,M:0.6%,n:364	
GCF_000022285.1	R_Dip_DroSim_A	Diptera	A	circular	1	1445873	C:98.9%[S:98.9%,D:0.0%],F:0.5%,M:0.6%,n:364	
GCF_014107475.1	R_Dip_DroStu_A	Diptera	A	circular	1	1185354	C:99.7%[S:99.2%,D:0.5%],F:0.0%,M:0.3%,n:364	
GCA_002300525.1	R_Dip_DroSub_A	Diptera	A	linear	106	1420341	C:99.2%[S:99.2%,D:0.0%],F:0.3%,M:0.5%,n:364	
GCA_000333795.2	R_Dip_DroSuz_A	Diptera	A	linear	110	1415350	C:99.2%[S:99.2%,D:0.0%],F:0.3%,M:0.5%,n:364	
GCA_005862135.1	R_Dip_DroTei_A	Diptera	A	linear	122	1301330	C:99.5%[S:99.5%,D:0.0%],F:0.0%,M:0.5%,n:364	
GCF_014129515.1	R_Dip_DroTri_A	Diptera	A	linear	9	1289544	C:98.9%[S:98.9%,D:0.0%],F:0.0%,M:1.1%,n:364	
GCF_014129525.1	R_Dip_DroTro_A	Diptera	A	linear	13	1215752	C:99.5%[S:99.5%,D:0.0%],F:0.0%,M:0.5%,n:364	

GCA_005862115.1	R_Dip_DroYak_A	Diptera	A	linear	118	1288853	C:99.5%[S:99.5%,D:0.0%],F:0.0%,M:0.5%,n:364	
GCA_009732755.1	R_Dip_HaeIrr_A	Diptera	A	circular	1	1352354	C:99.5%[S:99.5%,D:0.0%],F:0.0%,M:0.5%,n:364	
GCF_018454475.1	R_Dip_RagCer1_A	Diptera	A	linear	16	1255676	C:99.5%[S:99.2%,D:0.3%],F:0.0%,M:0.5%,n:364	
GCF_017604245.1	R_Dip_RhaCin_A	Diptera	A	circular	1	1538351	C:99.5%[S:99.5%,D:0.0%],F:0.0%,M:0.5%,n:364	
GCA_014333535.1	R_Hym_AnoGra_A	Hymenoptera	A	linear	96	1202684	C:99.7%[S:99.7%,D:0.0%],F:0.0%,M:0.3%,n:364	
GCF_010820705.1	R_Hym_AptDen_A	Hymenoptera	A	linear	49	1174448	C:94.8%[S:92.6%,D:2.2%],F:1.4%,M:3.8%,n:364	NZ_SDUV01000004.1, NZ_SDUV01000005.1, NZ_SDUV01000007.1, NZ_SDUV01000011.1, NZ_SDUV01000012.1, NZ_SDUV01000017.1, NZ_SDUV01000019.1, NZ_SDUV01000020.1, NZ_SDUV01000029.1, NZ_SDUV01000048.1, NZ_SDUV01000049.1 (mitochondrion host)
GCF_902713635.1	R_Hym_CarObs_A	Hymenoptera	A	linear	148	1194983	C:99.2%[S:98.9%,D:0.3%],F:0.0%,M:0.8%,n:364	
GCA_017869155.1	R_Hym_CerSol_A	Hymenoptera	A	circular	1	1206967	C:98.9%[S:98.9%,D:0.0%],F:0.3%,M:0.8%,n:364	
GCA_902648475.2	R_Hym_DiaAll_A	Hymenoptera	A	linear	169	1249264	C:97.3%[S:97%,D:0.3%],F:1.4%,M:1.3%,n:364	CACRJV020000057.1, CACRJV020000099.1, CACRJV020000109.1, CACRJV020000112.1, CACRJV020000115.1, CACRJV020000128.1, CACRJV020000140.1, CACRJV020000144.1, CACRJV020000153.1, CACRJV020000156.1 (Rickettsia)
GCA_902636375.1	R_Hym_DufNov_A	Hymenoptera	A	linear	221	1188140	C:95.3%[S:95.3%,D:0.0%],F:3.0%,M:1.7%,n:364	CACPRR010000135.1 (mitochondrion host)
GCA_902636315.1	R_Hym_LasAlb_A	Hymenoptera	A	linear	228	1195679	C:97.6%[S:97.3%,D:0.3%],F:1.9%,M:0.5%,n:364	CACPRV010000175.1 (Sodalis)
GCF_009012935.1	R_Hym_NasOne_A	Hymenoptera	A	linear	47	1293406	C:95.6%[S:95.3%,D:0.3%],F:0.3%,M:4.1%,n:364	
GCA_001983615.1	R_Hym_NasVit_A	Hymenoptera	A	linear	136	1198879	C:99.5%[S:99.2%,D:0.3%],F:0.0%,M:0.5%,n:364	MUJM01000137, MUJM01000138 (mitochondrion host), MUJM01000139-MUJM01000142 (Proteus)
GCA_001675785.1	R_Hym_NomFer_A	Hymenoptera	A	linear	231	1337641	C:98.6%[S:98.1%,D:0.5%],F:0.5%,M:0.9%,n:364	
GCF_001675695.1	R_Hym_NomFla_A	Hymenoptera	A	linear	167	1332780	C:98.7%[S:98.4%,D:0.3%],F:0.3%,M:1.0%,n:364	
GCA_001675715.1	R_Hym_NomLeu_A	Hymenoptera	A	linear	182	1366719	C:98.9%[S:98.9%,D:0.0%],F:0.3%,M:0.8%,n:364	
GCA_001675775.1	R_Hym_NomPan_A	Hymenoptera	A	linear	191	1344355	C:98.6%[S:95.9%,D:2.7%],F:0.5%,M:0.9%,n:364	
GCA_017869285.1	R_Hym_WiePum_A	Hymenoptera	A	circular	1	1278697	C:99.4%[S:97.5%,D:1.9%],F:0.0%,M:0.6%,n:364	
GCF_006542295.1	R_Lep_CarSas_A	Lepidoptera	A	circular	1	1449344	C:99.2%[S:99.2%,D:0.0%],F:0.0%,M:0.8%,n:364	
GCA_902646255.1	R_Ara_TetUrt_B	Arachnida	B	linear	149	1157560	C:99.5%[S:99.5%,D:0.0%],F:0.0%,M:0.5%,n:364	
GCF_020405475.1	R_Col_TriCon_B	Coleopta	B	linear	12	1418452	C:96.9%[S:96.4%,D:0.5%],F:0.0%,M:3.1%,n:364	
GCF_004171285.1	R_Dip_AedAlb_B	Diptera	B	circular	1	1484007	C:99.2%[S:98.4%,D:0.8%],F:0.3%,M:0.5%,n:364	
GCF_018491735.1	R_Dip_AnoDem_B	Diptera	B	linear	64	1232500	C:98.9%[S:98.9%,D:0.0%],F:0.0%,M:1.1%,n:364	
GCF_018491625.2	R_Dip_AnoMou_B	Diptera	B	circular	1	1121812	C:98.9%[S:98.6%,D:0.3%],F:0.3%,M:0.8%,n:364	
GCF_008245065.1	R_Dip_ChrMeg_B	Diptera	B	circular	1	1376868	C:99.7%[S:98.9%,D:0.8%],F:0.0%,M:0.3%,n:364	
GCA_000723225.2	R_Dip_CulMol_B	Diptera	B	linear	103	1435676	C:97.8%[S:97.5%,D:0.3%],F:1.1%,M:1.1%,n:364	
GCF_000073005.1	R_Dip_CulQui_B	Diptera	B	circular	1	1482455	C:99.4%[S:98.9%,D:0.5%],F:0.3%,M:0.3%,n:364	
GCF_004795975.1	R_Dip_DroMau_B	Diptera	B	circular	1	1273530	C:99.2%[S:98.9%,D:0.3%],F:0.3%,M:0.5%,n:364	
GCF_000376585.1	R_Dip_DroSim_B	Diptera	B	circular	1	1301823	C:99.8%[S:99.5%,D:0.3%],F:0.0%,M:0.2%,n:364	
GCF_018454445.1	R_Dip_RagCer5_B	Diptera	B	linear	57	1180723	C:98.3%[S:97.8%,D:0.5%],F:0.5%,M:1.2%,n:364	

GCF_003999585.1	R_Hem_BemTab_B	Hemiptera	B	circular	1	1306495	C:96.4%[S:94.8%,D:1.6%],F:1.4%,M:2.2%,n:364	
GCF_013458815.1	R_Hem_DiaCit_B	Hemiptera	B	circular	1	1656288	C:99.7%[S:99.2%,D:0.5%],F:0.0%,M:0.3%,n:364	
GCA_018224395.1	R_Hem_HomVit_B	Hemiptera	B	linear	1	1712771	C:98.9%[S:97.5%,D:1.4%],F:0.3%,M:0.8%,n:364	
GCF_007115015.1	R_Hem_LaoStr_B	Hemiptera	B	linear	2	1786382	C:99.4%[S:98.9%,D:0.5%],F:0.0%,M:0.6%,n:364	
GCF_007115045.1	R_Hem_NilLug_B	Hemiptera	B	linear	2	1542238	C:99.5%[S:98.4%,D:1.1%],F:0.0%,M:0.5%,n:364	
GCF_006334525.1	R_Hym_LepCla_B	Hymenoptera	B	linear	46	1150755	C:98.9%[S:98.4%,D:0.5%],F:0.3%,M:0.8%,n:364	
GCF_001439985.1	R_Hym_TriPre_B	Hymenoptera	B	circular	1	1133809	C:99.5%[S:99.5%,D:0.0%],F:0.0%,M:0.5%,n:364	
GCF_001027565.1	R_Iso_ArmVul_B	Isopoda	B	linear	10	1663741	C:97.8%[S:97.5%,D:0.3%],F:1.1%,M:1.1%,n:364	
GCF_020995475.1	R_Lep_CorCep_B	Lepidoptera	B	circular	1	1359904	C:99.5%[S:99.5%,D:0.0%],F:0.0%,M:0.5%,n:364	
GCF_018555315.1	R_Lep_EreCas_B	Lepidoptera	B	linear	2	1423447	C:94.5%[S:94.2%,D:0.3%],F:0.0%,M:5.5%,n:364	
GCF_000333775.1	R_Lep_HypBol_B	Lepidoptera	B	linear	144	1377933	C:99.2%[S:98.9%,D:0.3%],F:0.3%,M:0.5%,n:364	
GCF_001266585.1	R_Lep_OphBru_B	Lepidoptera	B	linear	120	1121046	C:98.1%[S:98.1%,D:0.0%],F:0.5%,M:1.4%,n:364	
GCA_902636855.1	R_Lep_ParAeg_B	Lepidoptera	B	linear	64	1268638	C:99.5%[S:99.5%,D:0.0%],F:0.3%,M:0.2%,n:364	
GCA_002318985.1	R_Lep_PluAus_B	Lepidoptera	B	linear	95	1158805	C:99.7%[S:99.7%,D:0.0%],F:0.0%,M:0.3%,n:364	
GCF_018141665.1	R_Lep_SpoPic_B	Lepidoptera	B	circular	1	1339720	C:99.4%[S:98.9%,D:0.5%],F:0.0%,M:0.6%,n:364	
GCF_013365455.1	R_Nem_DirImm_C	Nematoda	C	circular	1	920122	C:94.5%[S:94.5%,D:0.0%],F:0.3%,M:5.2%,n:364	
GCF_000306885.1	R_Nem_OncOch_C	Nematoda	C	circular	1	957990	C:95.1%[S:95.1%,D:0.0%],F:0.0%,M:4.9%,n:364	
GCF_000530755.1	R_Nem_OncVol_C	Nematoda	C	linear	1	960618	C:94.8%[S:94.8%,D:0.0%],F:0.3%,M:4.9%,n:364	
GCF_000008385.1	R_Nem_BruMal_D	Nematoda	D	circular	1	1080084	C:98.6%[S:98.6%,D:0.0%],F:0.3%,M:1.1%,n:364	
GCF_012030695.1	R_Nem_BruPah_D	Nematoda	D	circular	1	1072967	C:98.4%[S:98.4%,D:0.0%],F:0.3%,M:1.3%,n:364	
GCF_013366805.1	R_Nem_LitBra_D	Nematoda	D	linear	41	1046149	C:94.5%[S:94.5%,D:0.0%],F:0.0%,M:5.5%,n:364	
GCF_013365435.1	R_Nem_LitSig_D	Nematoda	D	circular	1	1045802	C:95.1%[S:95.1%,D:0.0%],F:0.3%,M:4.6%,n:364	
GCF_002204235.2	R_Nem_WucBan_D	Nematoda	D	linear	100	1060850	C:95.3%[S:95.3%,D:0.0%],F:1.9%,M:2.8%,n:364	
GCA_019061405.1	R_Ara_FraSet_E	Arachnida	E	linear	30	1082514	C:98.4%[S:98.1%,D:0.3%],F:0.3%,M:1.3%,n:364	
GCF_001931755.2	R_Col_FolCan_E	Collembola	E	circular	1	1801626	C:99.5%[S:99.2%,D:0.3%],F:0.0%,M:0.5%,n:364	
GCF_014534705.1	R_Hem_PenNig_E	Hemiptera	E	linear	182	1457187	C:98.7%[S:98.4%,D:0.3%],F:0.8%,M:0.5%,n:364	
GCA_001752665.1	R_Nem_PratPen_E	Nematoda	E	linear	12	975127	C:95.6%[S:95.1%,D:0.5%],F:2.7%,M:1.7%,n:364	
GCF_012277295.1	R_Sip_CteFel_E	Siphonaptera	E	circular	1	1495538	C:98.9%[S:97.0%,D:1.9%],F:0.3%,M:0.8%,n:364	
GCF_000829315.1	R_Hem_CimLex_F	Hemiptera	F	circular	1	1250060	C:97.8%[S:97.3%,D:0.5%],F:0.8%,M:1.4%,n:364	
GCA_902636395.1	R_Hym_OsmCae_F	Hymenoptera	F	linear	163	1232261	C:98.7%[S:98.4%,D:0.3%],F:0.5%,M:0.8%,n:364	
GCF_013366855.1	R_Nem_MadHie_F	Nematoda	F	linear	208	1025329	C:93.1%[S:92.6%,D:0.5%],F:3.8%,M:3.1%,n:364	
GCF_020278625.1	R_Nem_ManOzz_F	Nematoda	F	linear	93	1073310	C:95.3%[S:95.3%,D:0.0%],F:1.6%,M:3.1%,n:364	
GCF_012277315.1	R_Sip_CteFel_F	Siphonaptera	F	circular	1	1201647	C:99.5%[S:99.2%,D:0.3%],F:0.3%,M:0.2%,n:364	
GCF_013365475.1	R_Nem_CruTub_J	Nematoda	J	circular	1	863988	C:94.0%[S:94.0%,D:0.0%],F:0.8%,M:5.2%,n:364	
GCF_013365495.1	R_Nem_DipCau_J	Nematoda	J	circular	1	863427	C:92.3%[S:92.3%,D:0.0%],F:0.5%,M:7.2%,n:364	
GCA_013317055.1	R_Ara_AtePolK5_S	Arachnida	S	linear	200	1404177	C:86.5%[S:83.2%,D:3.3%],F:5.8%,M:7.7%,n:364	
GCF_013309895.1	R_Ara_AtePolK3_S	Arachnida	S	linear	373	1445964	C:88.5%[S:83.8%,D:4.7%],F:3.8%,M:7.7%,n:364	
