# Supplementary material for: Phylogenomic analysis of Wolbachia genomes from the Darwin Tree of Life biodiversity genomics project
Source: PLoS Biol. 2023 Jan 23;21(1):e3001972. doi: 10.1371/journal.pbio.3001972 (PMC9894559; doi:10.1371/journal.pbio.3001972)
Supplement: S4 Table — (PDF) [file pbio.3001972.s005.pdf]

S4 Table. Gene modules used to identify prophage regions

Module	Gene family	Gene identifier
Connector /Baseplate	OG0000021	gpI
Connector /Baseplate	OG0000032	gpJ
Connector /Baseplate	OG0000034	gpW
Connector /Baseplate	OG0000058	PAAR
Connector /Baseplate	OG0000045	gpV
Connector /Baseplate	OG0000048	Collar
Connector /Baseplate	OG0000044	gpZ
Connector /Baseplate	OG0000041	gpFII
Head	OG0000036	Major capsid protein GpE
Head	OG0000042	Head decoration protein D
Head	OG0000035	Minor capsid protein Orf7
Head	OG0000025	Phage portal protein, lambda family
Head	OG0000049	Head-to-tail joining protein W
Head	OG0000030	Phage terminase
Tail	OG0000068	Tail sheath protein
Tail	OG0000064	Major tail tube protein
Tail	OG0000075	gpG/GT
Tail	OG0000059	Phage tail tape measure protein
Tail	OG0000080	gpX (Tail protein X)
Tail	OG0000066	gpD (late control)
Fibre	OG0000088	Hypothetical protein
Fibre	OG0000069	Domain of unknown function DUF4815
Fibre	OG0000077	Hypothetical protein
Fibre	OG0000100	Hypothetical protein
Fibre	OG0000079	Hypothetical protein
Fibre	OG0000104	Hypothetical protein
Phage	OG0000000	ankyrin repeat domain-containing protein,IS4 family transposase
Phage	OG0000002	IS5 family transposase, IS5-like element ISWpi1 family transposase
Phage	OG0000006	Rpn family recombination-promoting nuclease/putative transposase
Phage	OG0000007	IS630 family transposase
Phage	OG0000008	IS66-like element ISWen3 family transposase, IS66 family transposase
Phage	OG0000009	IS630 transposase-related protein,IS630 family transposase
Phage	OG0000017	IS110-like element ISWen2 family transposase
Phage	OG0000020	recombinase family protein
Phage	OG0000023	IS982 family transposase
Phage	OG0000024	DUF6290 family protein

Phage	OG0000029	AAA family ATPase
Phage	OG0000038	ankyrin repeat domain-containing protein
Phage	OG0000039	type II toxin-antitoxin system RelE/ParE family toxin,type II toxin-antitoxin system mRNA interferase toxin, RelE/StbE family
Phage	OG0000040	HNH endonuclease,hypothetical protein,reverse transcriptase N-terminal domain-containing protein
Phage	OG0000043	AAA family ATPase
Phage	OG0000047	ankyrin repeat domain-containing protein
Phage	OG0000051	patatin-like phospholipase family protein
Phage	OG0000055	hypothetical protein
Phage	OG0000056	ankyrin repeat domain-containing protein
Phage	OG0000057	Holliday junction resolvase,hypothetical protein,endonuclease
Phage	OG0000060	DUF2924 domain-containing protein
Phage	OG0000061	Ulp1 family isopeptidase
Phage	OG0000062	DNA modification methylase
Phage	OG0000063	IS110-like element ISWpi13 family transposase,IS110 family transposase
Phage	OG0000074	hypothetical protein
Phage	OG0000078	ATP-binding protein
Phage	OG0000084	ankyrin repeat domain-containing protein
Phage	OG0000090	hypothetical protein
Phage	OG0000094	sigma-70 family RNA polymerase sigma factor
Phage	OG0000108	hypothetical protein
Phage	OG0000109	phospholipase D family protein
Phage	OG0000113	DUF1016 family protein,DUF1016 N-terminal domain-containing protein,DUF1016 domain-containing protein,PDDEXK nuclease domain-containing protein
Phage	OG0000114	NAD-dependent DNA ligase LigA
Phage	OG0000121	hypothetical protein
Phage	OG0000214	XRE family transcriptional regulator,helix-turn-helix domain-containing protein
Phage	OG0000845	IS110 family transposase
Phage	OG0000900	glycoside hydrolase family 25 protein
Phage	OG0000938	AAA family ATPase
Phage	OG0000950	XRE family transcriptional regulator
Phage	OG0000951	recombinase family protein
Phage	OG0001032	ankyrin repeat domain-containing protein
Phage	OG0001048	hypothetical protein
Phage	OG0001070	hypothetical protein
Phage	OG0001075	ankyrin repeat domain-containing protein
Phage	OG0001088	hypothetical protein
Phage	OG0001097	ankyrin repeat domain-containing protein
Phage	OG0001102	site-specific DNA-methyltransferase
Phage	OG0001157	ankyrin repeat domain-containing protein

Phage	OG0001253	ankyrin repeat domain-containing protein
Phage	OG0001277	hypothetical protein
Phage	OG0001298	DUF5372 family protein
Phage	OG0001510	hypothetical protein
Phage	OG0001530	hypothetical protein
Phage	OG0001544	hypothetical protein
Phage	OG0001578	XRE family transcriptional regulator
Phage	OG0001601	hypothetical protein
Phage	OG0001671	hypothetical protein
Phage	OG0001721	ankyrin repeat domain-containing protein
Phage	OG0001855	hypothetical protein
Phage	OG0002322	hypothetical protein
Phage	OG0002323	ankyrin repeat domain-containing protein
Phage	OG0002331	ankyrin repeat domain-containing protein
Phage	OG0002332	type IV toxin-antitoxin system AbiEi family antitoxin
Phage	OG0002333	nucleotidyl transferase AbiEii/AbiGii toxin family protein
Undecim	OG0000095	bifunctional UDP-N-acetylglucosamine diphosphorylase/glucosamine-1-phosphate N-acetyltransferase GlmU,NTP transferase domain-containing protein
Undecim	OG0000141	ABC transporter ATP-binding protein/permease,ABC transporter ATP-binding protein
Undecim	OG0000258	DMT family transporter
Undecim	OG0000868	aminotransferase class I/II-fold pyridoxal phosphate-dependent enzyme,threonine aldolase family protein
Undecim	OG0000874	UDP-glucose/GDP-mannose dehydrogenase family protein
Undecim	OG0000879	MFS transporter
Undecim	OG0000893	phytanoyl-CoA dioxygenase family protein
Undecim	OG0000894	threonine aldolase family protein
Undecim	OG0000897	hypothetical protein,WG repeat-containing protein
Undecim	OG0000899	glycosyltransferase
Undecim	OG0000902	SDR family oxidoreductase,NAD(P)-dependent oxidoreductase,GDP-mannose 4,6-dehydratase
Undecim	OG0001775	glycosyltransferase
EAM	OG0000001	IS5 family transposase,transposase
EAM	OG0000003	IS256 family transposase,hypothetical protein
EAM	OG0000005	helix-turn-helix domain-containing protein,helix-turn-helix transcriptional regulator
EAM	OG0000010	ankyrin repeat domain-containing protein,hypothetical protein
EAM	OG0000012	DNA repair protein RadC
EAM	OG0000014	hypothetical protein
EAM	OG0000018	group II intron reverse transcriptase/maturase
EAM	OG0000022	hypothetical protein
EAM	OG0000027	IS4 family transposase
EAM	OG0000037	ankyrin repeat domain-containing protein,hypothetical protein

EAM	OG0000050	helix-turn-helix domain-containing protein
EAM	OG0000052	group II intron reverse transcriptase/maturase,hypothetical protein
EAM	OG0000054	PD-(D/E)XK nuclease family transposase,Rpn family recombination-promoting nuclease/putative transposase
EAM	OG0000072	IS3 family transposase,hypothetical protein
EAM	OG0000087	cytoplasmic incompatibility factor CifA,hypothetical protein
EAM	OG0000092	DNA mismatch repair endonuclease MutL
EAM	OG0000093	hypothetical protein
EAM	OG0000112	hypothetical protein,Rpn family recombination-promoting nuclease/putative transposase
EAM	OG0000118	hypothetical protein
EAM	OG0000134	hypothetical protein
EAM	OG0000137	integrase core domain-containing protein,IS481-like element ISWpi4 family transposase,IS481 family transposase,DDE-type integrase/transposase/recombinase
EAM	OG0000140	virulence RhuM family protein,hypothetical protein
EAM	OG0000154	pyrimidine dimer DNA glycosylase/endonuclease V,hypothetical protein
EAM	OG0000156	hypothetical protein
EAM	OG0000158	hypothetical protein
EAM	OG0000165	hypothetical protein
EAM	OG0000221	IS4 family transposase
EAM	OG0000846	ankyrin repeat domain-containing protein
EAM	OG0000910	ankyrin repeat domain-containing protein
EAM	OG0000919	hypothetical protein
EAM	OG0000945	Hsp20/alpha crystallin family protein
EAM	OG0000969	DEAD/DEAH box helicase
EAM	OG0000983	Fic family protein
EAM	OG0001000	hypothetical protein,ankyrin repeat domain-containing protein
EAM	OG0001005	hypothetical protein
EAM	OG0001006	hypothetical protein
EAM	OG0001017	ankyrin repeat domain-containing protein,hypothetical protein
EAM	OG0001024	ankyrin repeat domain-containing protein
EAM	OG0001039	hypothetical protein
EAM	OG0001047	hypothetical protein
EAM	OG0001062	ankyrin repeat domain-containing protein
EAM	OG0001104	ankyrin repeat domain-containing protein
EAM	OG0001106	hypothetical protein
EAM	OG0001128	ankyrin repeat domain-containing protein
EAM	OG0001155	ankyrin repeat domain-containing protein
EAM	OG0001179	AAA family ATPase
EAM	OG0001188	ankyrin repeat domain-containing protein
EAM	OG0001213	hypothetical protein

EAM	OG0001218	ankyrin repeat domain-containing protein
EAM	OG0001274	hypothetical protein
EAM	OG0001432	ATP-binding protein
EAM	OG0001554	hypothetical protein
