# Supplementary material for: Phylogenomic analysis of Wolbachia genomes from the Darwin Tree of Life biodiversity genomics project
Source: PLoS Biol. 2023 Jan 23;21(1):e3001972. doi: 10.1371/journal.pbio.3001972 (PMC9894559; doi:10.1371/journal.pbio.3001972)
Supplement: S5 Table — (PDF) [file pbio.3001972.s006.pdf]

S5 Table. Summary toxin genes

	A	B	C	D	E	F	J	S
**CifA-CifB**	98/144(76)	115/159 (64)				0/2 (2)		
**RelE/RelB**	131/381 (106)	41/164 (61)		0/2 (2)	3/4 (2)	0/7 (2)		
**Latrotoxin**	36/65 (36)	93/187 (75)			1/1 (1)	0/2 (2)		
**TcA**	26/34 (20)	16/35 (15)						
**TcB-C**	7/11 (7)	12/24 (13)						
**ParD**	1/23(23)	0/37 (37)			2/3 (3)	0/1 (1)		
**ParE**	0/8 (8)	0/34 (34)			1/2 (2)			
**FIC**	3/99 (93)				1/4 (3)	0/3 (3)		0/2 (2)
**AbiEii/AbiGii-AbiEi**					1/2 (2)			

Number of genes located in the prophage region compared to the total number of toxin genes. In brackets is the number of species containing the toxin.
