# Supplementary material for: Phylogenomic analysis of Wolbachia genomes from the Darwin Tree of Life biodiversity genomics project
Source: PLoS Biol. 2023 Jan 23;21(1):e3001972. doi: 10.1371/journal.pbio.3001972 (PMC9894559; doi:10.1371/journal.pbio.3001972)
Supplement: S1 Fig — Selected tissue and incidence of Wolbachia presence (green) and absence (purple) of DToL samples. (PDF) [file pbio.3001972.s007.pdf]

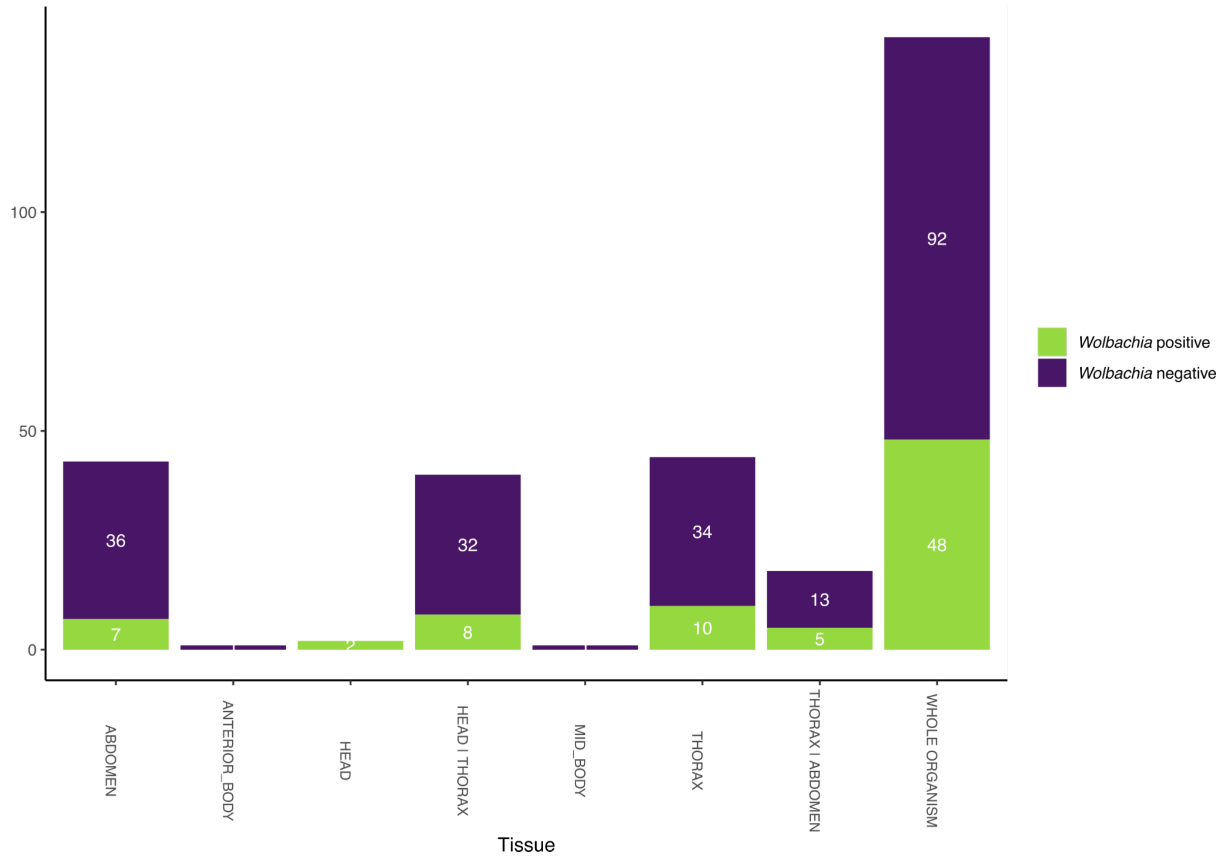

S1 Fig. Selected tissue and incidence of *Wolbachia* presence (green) and absence (purple) of DToL samples.
