# Supplementary material for: Phylogenomic analysis of Wolbachia genomes from the Darwin Tree of Life biodiversity genomics project
Source: PLoS Biol. 2023 Jan 23;21(1):e3001972. doi: 10.1371/journal.pbio.3001972 (PMC9894559; doi:10.1371/journal.pbio.3001972)
Supplement: S2 Fig — Sampling locations and incidence of Wolbachia presence (green) and absence (purple) of DToL samples from Britain and Ireland. The map was drawn using the maps library (version 3.4.0) in R, which imports data from the public domain (Natural Earth project) (https://www.naturalearthdata.com/downloads/50m-physical-vectors/). The size of the pie charts reflects the number of collected samples per location. Most samples came from Wytham Woods Genomic Observatory near Oxford. The data underlying this Figure can be found in S1 Data. (PDF) [file pbio.3001972.s008.pdf]

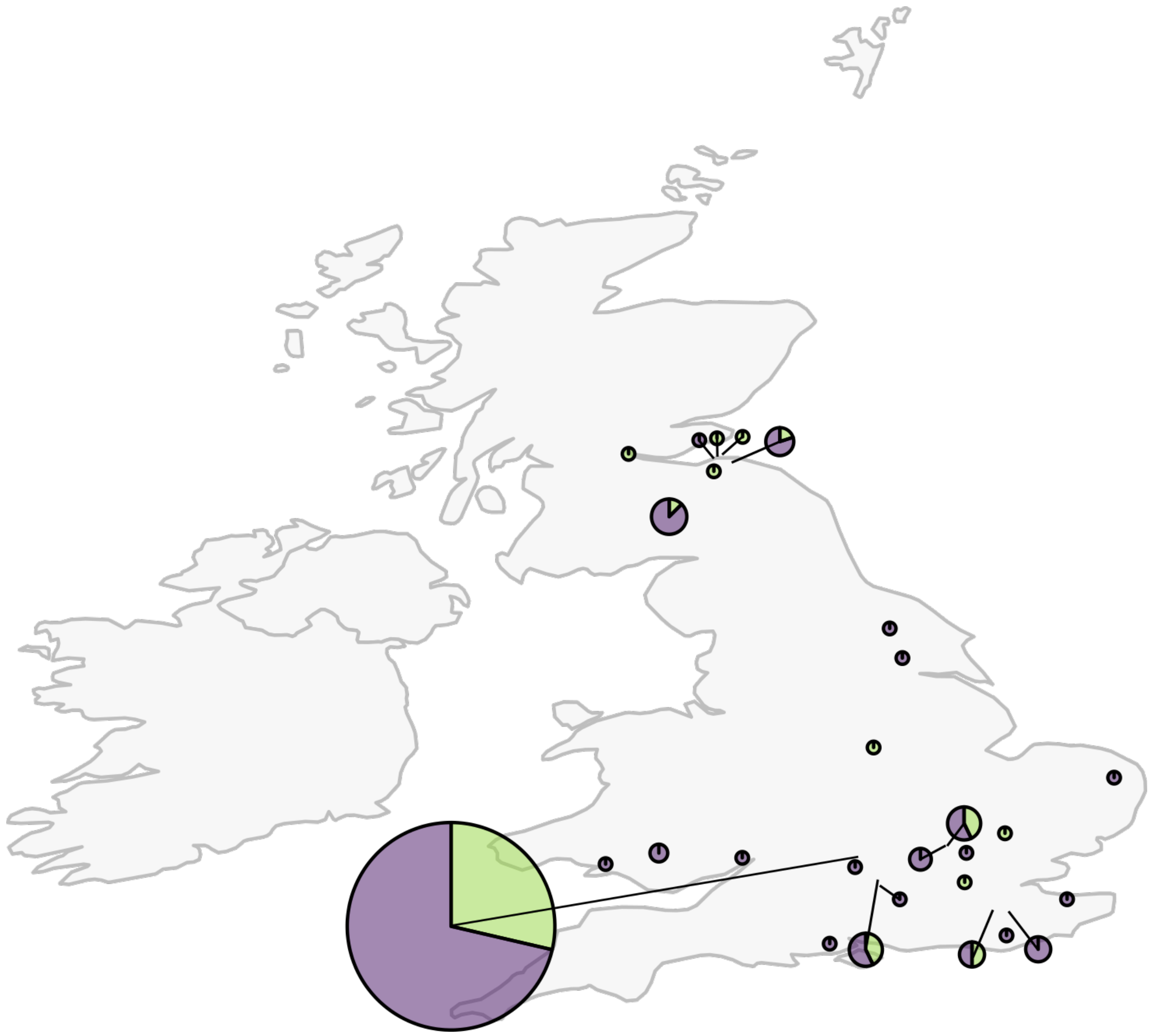

S2 Fig. Sampling locations and incidence of *Wolbachia* presence (green) and absence (purple) of DToL samples from Britain and Ireland. The map was drawn using the maps library (version 3.4.0) in R, which imports data from the public domain (Natural Earth project) (https://www.naturalearthdata.com/downloads/50m-physical-vectors/). The size of the pie charts reflects the number of collected samples per location. Most samples came from Wytham Woods Genomic Observatory near Oxford. The data underlying this Figure can be found in S1 Data.
