# Supplementary material for: Phylogenomic analysis of Wolbachia genomes from the Darwin Tree of Life biodiversity genomics project
Source: PLoS Biol. 2023 Jan 23;21(1):e3001972. doi: 10.1371/journal.pbio.3001972 (PMC9894559; doi:10.1371/journal.pbio.3001972)
Supplement: S3 Fig — (A, B) Contiguity and genome size distribution of Wolbachia genomes assembled in this study (black) vs. reference genomes from other projects available in NCBI (grey). The data underlying this Figure can be found in S1 Data. (C) Genome size distribution of Wolbachia. Supergroups A (above) and B (below), in this study (black) and reference genomes from other projects available in NCBI (grey) were compared by Wilcoxon rank sum test. The data underlying this Figure can be found in S1 Data. (PDF) [file pbio.3001972.s009.pdf]

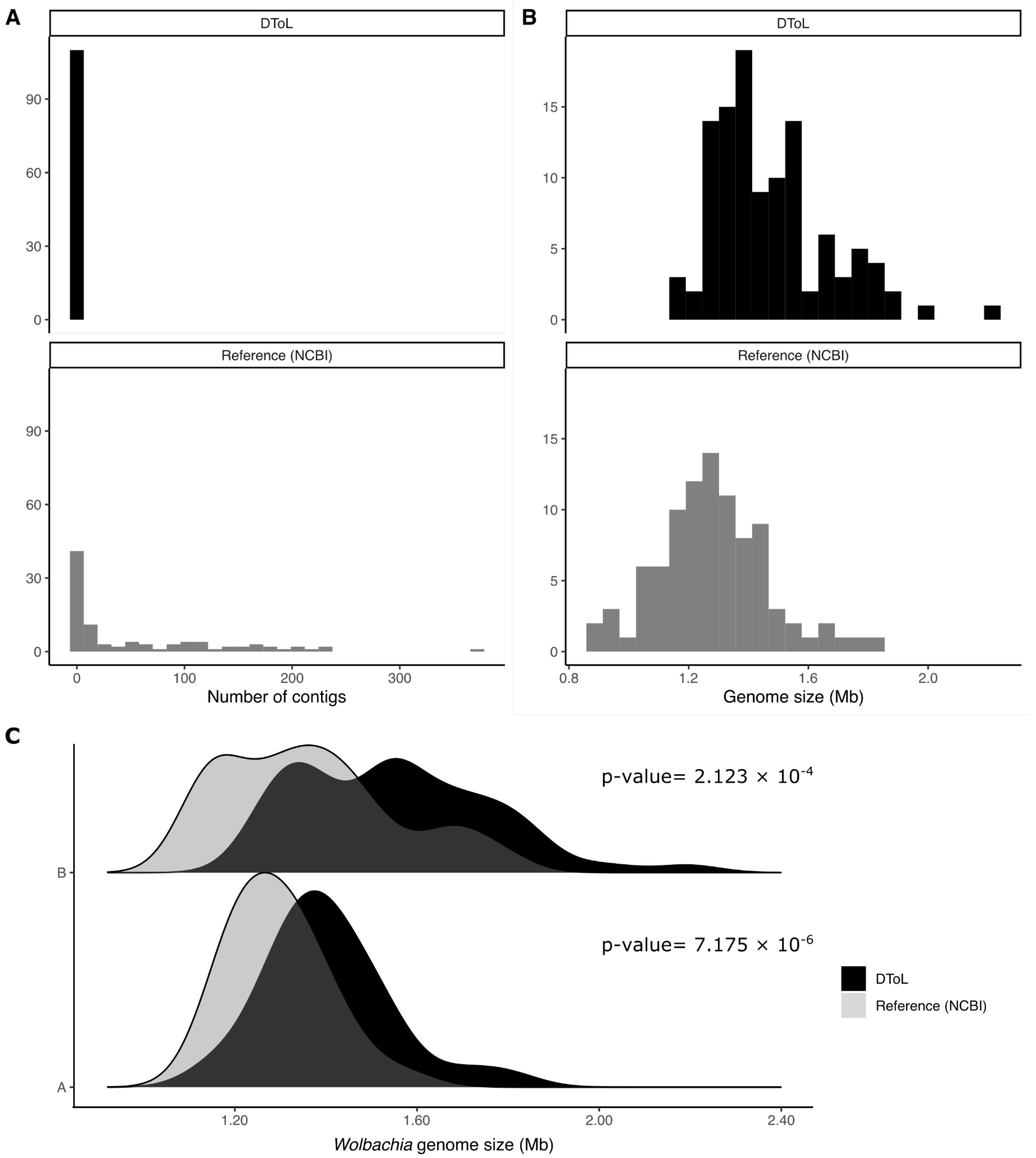

S3 Fig. A,B Contiguity and genome size distribution of *Wolbachia* genomes assembled in this study (black) vs reference genomes from other projects available in NCBI (grey). The data underlying this Figure can be found in S1 Data. C. Genome size distribution of *Wolbachia*. supergroup A (above) and B (below), in this study (black) and reference genomes from other projects available in NCBI (grey) were compared by Wilcoxon rank sum test. The data underlying this Figure can be found in S1 Data.
