# Supplementary material for: Phylogenomic analysis of Wolbachia genomes from the Darwin Tree of Life biodiversity genomics project
Source: PLoS Biol. 2023 Jan 23;21(1):e3001972. doi: 10.1371/journal.pbio.3001972 (PMC9894559; doi:10.1371/journal.pbio.3001972)
Supplement: S4 Fig — Phylogeny of supergroup A and B Wolbachia, visualised with the root placed between the A and B supergroups and the remaining supergroups (C, D, E, F, J, S; nodes collapsed as grey wedge), highlighting nodes with bootstrap value higher than 80 with a black label. (PDF) [file pbio.3001972.s010.pdf]

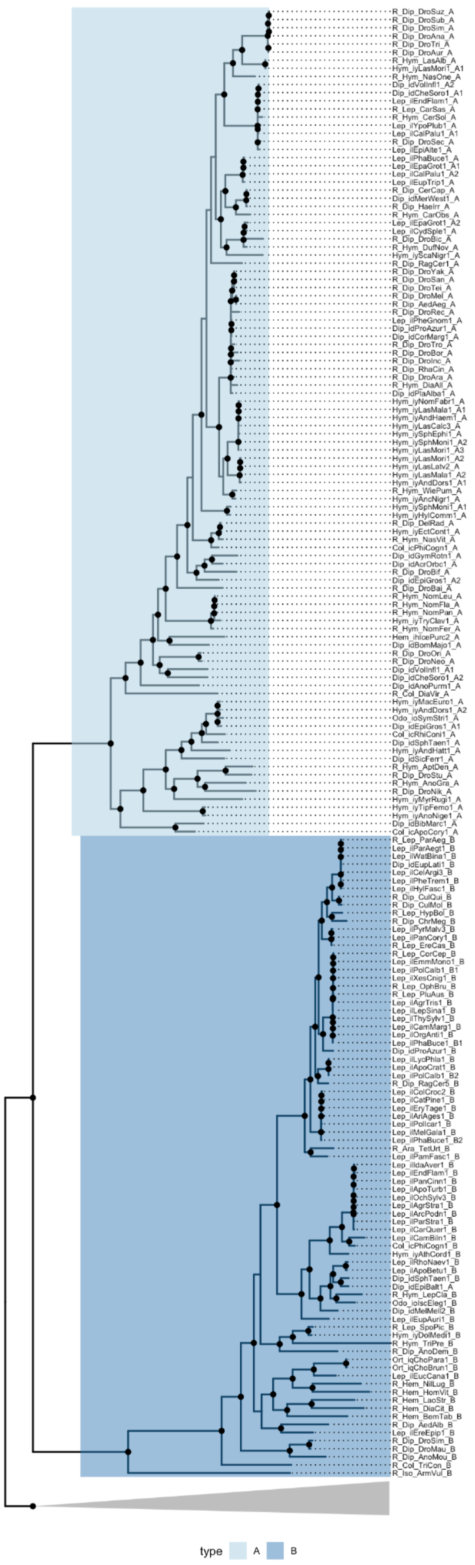

S4 Fig. Phylogeny of supergroup A and B *Wolbachia*, visualised with the root placed between the A and B supergroups and the remaining supergroups (C,D,E,F, J, S; nodes collapsed as grey wedge), highlighting nodes with bootstrap value higher than 80 with a black label.
