# Supplementary material for: Phylogenomic analysis of Wolbachia genomes from the Darwin Tree of Life biodiversity genomics project
Source: PLoS Biol. 2023 Jan 23;21(1):e3001972. doi: 10.1371/journal.pbio.3001972 (PMC9894559; doi:10.1371/journal.pbio.3001972)
Supplement: S5 Fig — Distribution of average nucleotide identity (ANI) between pairs of Wolbachia genomes if specimens were both sampled from Wytham Woods (upper panel) or any other locality (lower panel). Distributions are separated by the classification of the two genomes, i.e., both belonging to supergroup A, both belonging to supergroup B, comparisons of A with B, or comparisons between other supergroups. The data underlying this Figure can be found in S1 Data. (PDF) [file pbio.3001972.s011.pdf]

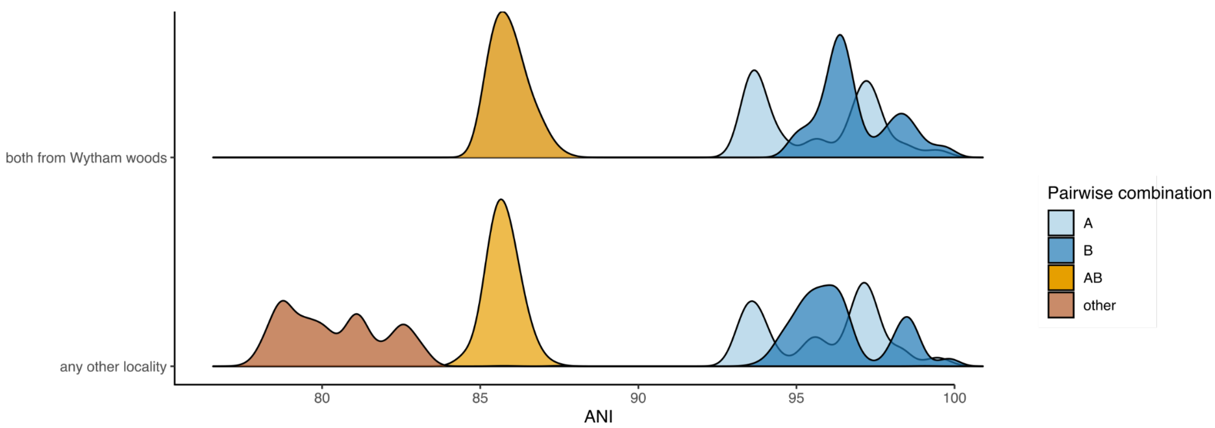

S5 Fig. Distribution of average nucleotide identity (ANI) between pairs of *Wolbachia* genomes if specimens were both sampled from Wytham Woods (upper panel) or any other locality (lower panel). Distributions are separated by the classification of the two genomes, i.e. both belonging to supergroup A, both belonging to supergroup B, comparisons of A with B, or comparisons between other supergroups. The data underlying this Figure can be found in S1 Data.
