# Supplementary material for: Phylogenomic analysis of Wolbachia genomes from the Darwin Tree of Life biodiversity genomics project
Source: PLoS Biol. 2023 Jan 23;21(1):e3001972. doi: 10.1371/journal.pbio.3001972 (PMC9894559; doi:10.1371/journal.pbio.3001972)
Supplement: S6 Fig — Number of predicted protein-coding genes for Wolbachia supergroups A (above) and B (below), in this study (black) and reference genomes from other projects available in NCBI (grey) were compared by Wilcoxon rank sum test. The data underlying this Figure can be found in S1 Data. (PDF) [file pbio.3001972.s012.pdf]

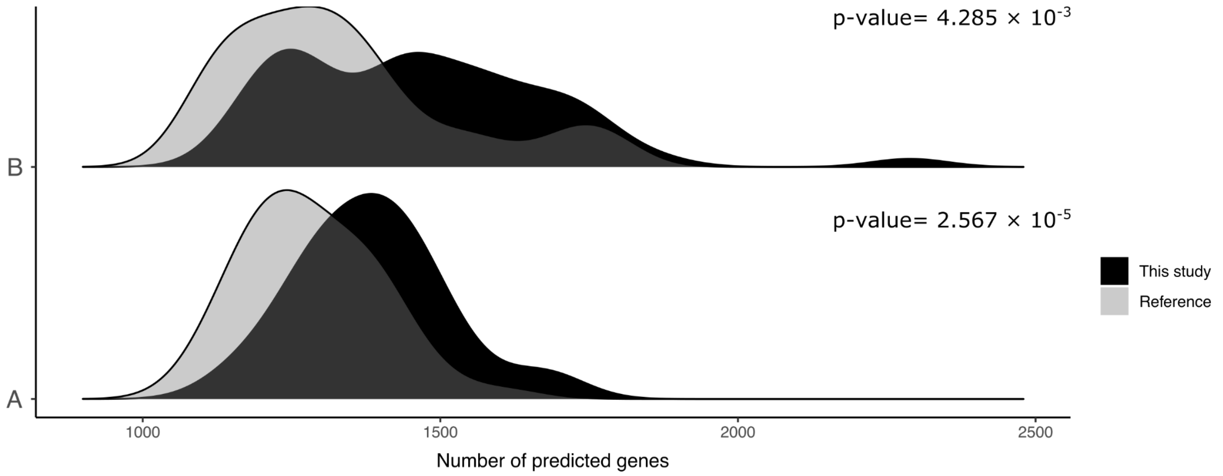

S6 Fig. Number of predicted protein-coding genes for *Wolbachia* supergroup A (above) and B (below), in this study (black) and reference genomes from other projects available in NCBI (grey) were compared by Wilcoxon rank sum test. The data underlying this Figure can be found in S1 Data.
