# Supplementary material for: Phylogenomic analysis of Wolbachia genomes from the Darwin Tree of Life biodiversity genomics project
Source: PLoS Biol. 2023 Jan 23;21(1):e3001972. doi: 10.1371/journal.pbio.3001972 (PMC9894559; doi:10.1371/journal.pbio.3001972)
Supplement: S7 Fig — Percentage of protein-coding genes present in WO prophage regions versus percentage of strain-specific protein-coding genes in those regions of Wolbachia genomes with at least 10 strain-specific genes. Size of points is reflective of the total number of strain-specific genes. Linear regression line with confidence interval is displayed. The data underlying this Figure can be found in S1 Data. (PDF) [file pbio.3001972.s013.pdf]

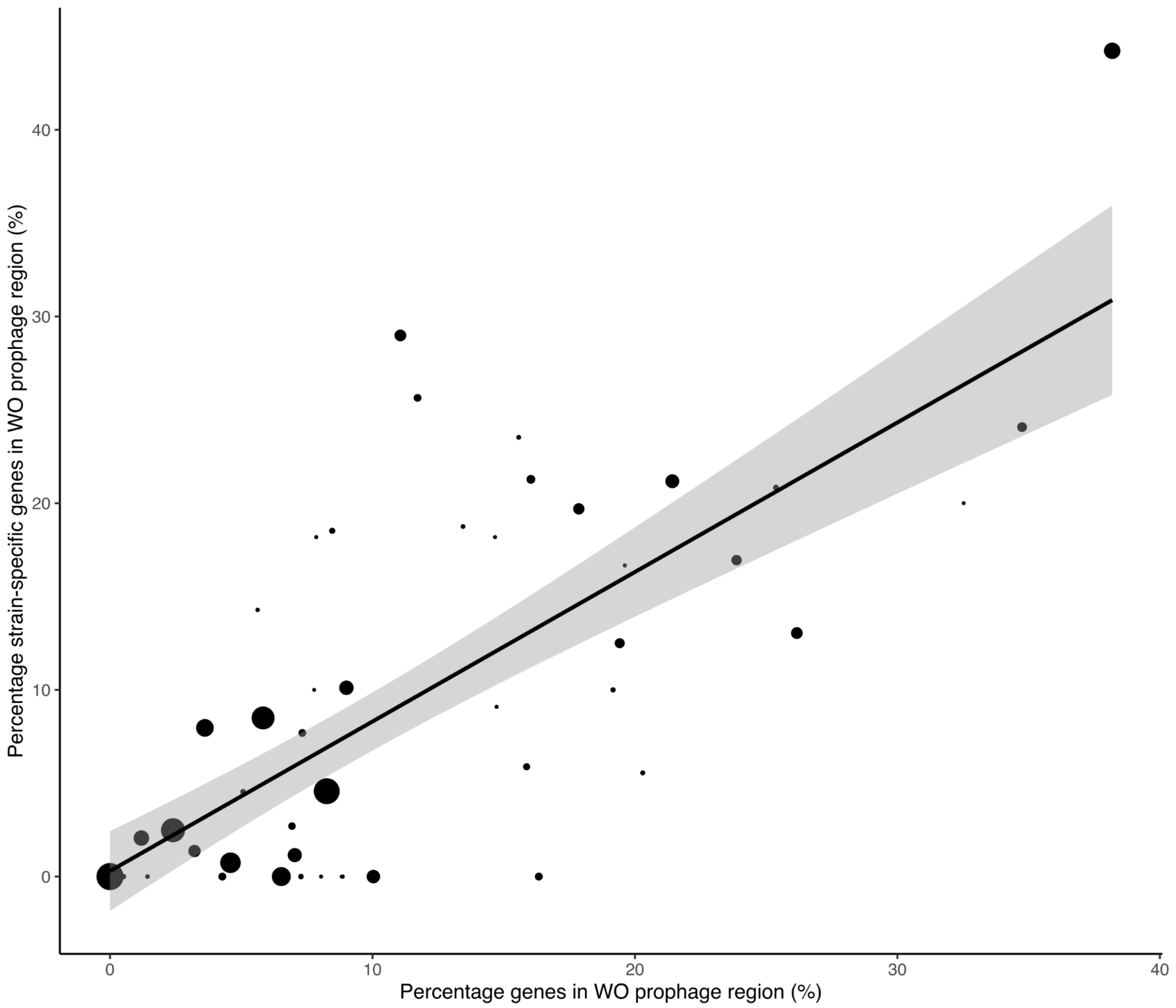

S7 Fig. Percentage of protein-coding genes present in WO prophage regions versus percentage of strain-specific protein-coding genes in those regions of *Wolbachia* genomes with at least 10 strain-specific genes. Size of points is reflective of the total number of strain-specific genes. Linear regression line with confidence interval is displayed. The data underlying this Figure can be found in S1 Data.
