# Supplementary material for: Phylogenomic analysis of Wolbachia genomes from the Darwin Tree of Life biodiversity genomics project
Source: PLoS Biol. 2023 Jan 23;21(1):e3001972. doi: 10.1371/journal.pbio.3001972 (PMC9894559; doi:10.1371/journal.pbio.3001972)
Supplement: S8 Fig — Comparison between phylogenies of Wolbachia genomes containing the biotin locus, based on tree in Fig 2A (left) and a phylogeny inferred from the six nucleotide genes constituting the biotin synthesis operon (BioA-D, BioF, BioH) (right). Internal nodes with bootstrap support higher than 80 are highlighted with black circles. (PDF) [file pbio.3001972.s014.pdf]

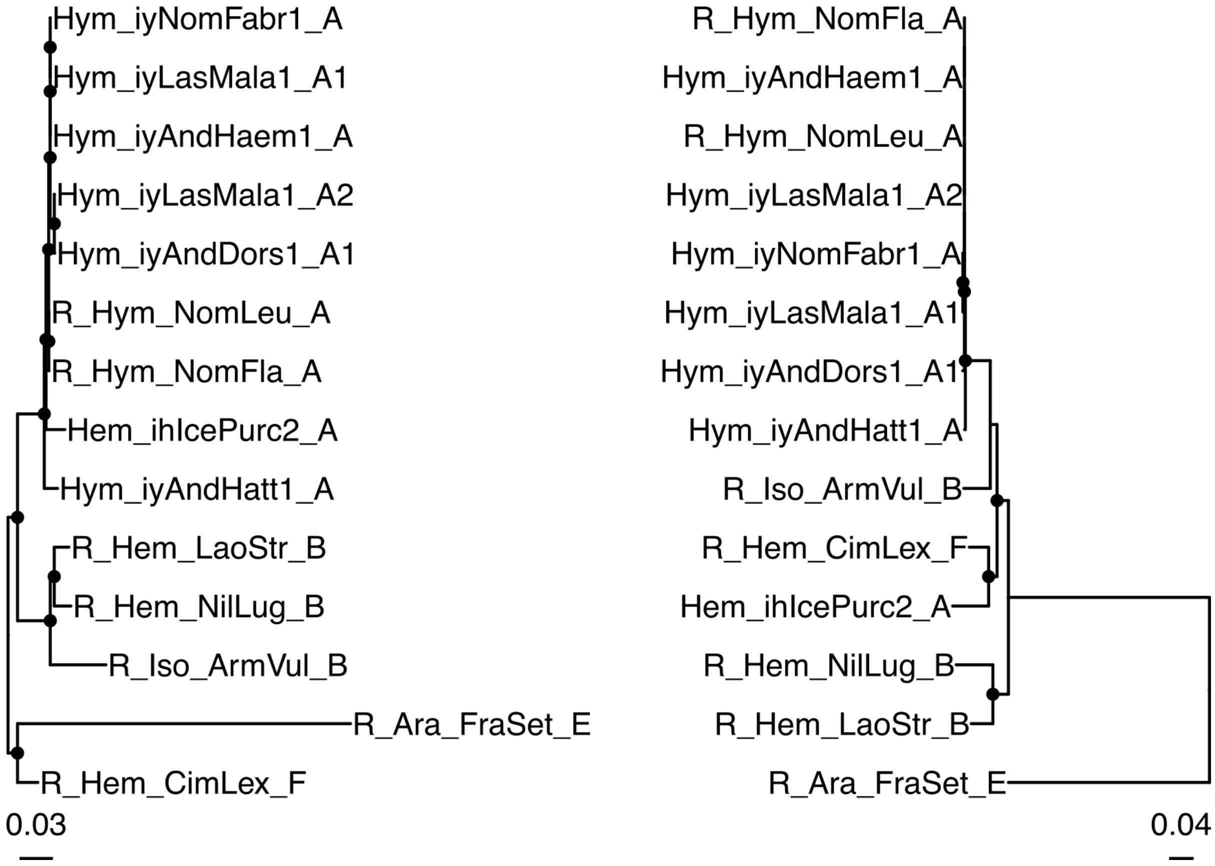

S8 Fig. Comparison between phylogenies of *Wolbachia* genomes containing the biotin locus, based on tree in Fig 2A (left) and a phylogeny inferred from the six nucleotide genes constituting the biotin synthesis operon (BioA-D,BioF, BioH) (right). Internal nodes with bootstrap support higher than 80 are highlighted with black circles.
