# Supplementary material for: Phylogenomic analysis of Wolbachia genomes from the Darwin Tree of Life biodiversity genomics project
Source: PLoS Biol. 2023 Jan 23;21(1):e3001972. doi: 10.1371/journal.pbio.3001972 (PMC9894559; doi:10.1371/journal.pbio.3001972)
Supplement: S10 Fig — Phylogeny of CifB toxin genes, highlighting nodes with a bootstrap value higher than 80 with a circle. (PDF) [file pbio.3001972.s016.pdf]

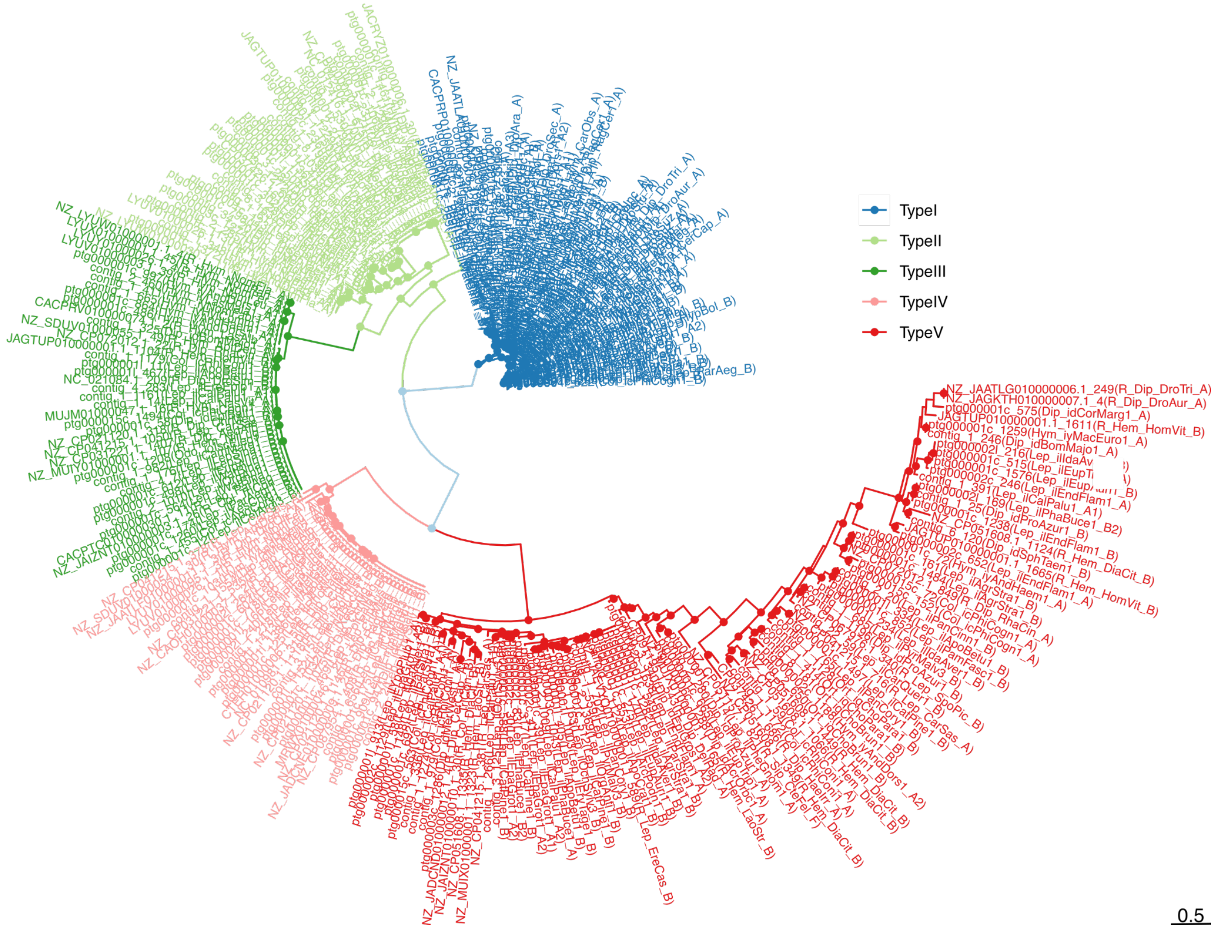

S10 Fig. Phylogeny of CifB toxin genes, highlighting nodes with a bootstrap value higher than 80 with a circle.
