# Supplementary material for: Phylogenomic analysis of Wolbachia genomes from the Darwin Tree of Life biodiversity genomics project
Source: PLoS Biol. 2023 Jan 23;21(1):e3001972. doi: 10.1371/journal.pbio.3001972 (PMC9894559; doi:10.1371/journal.pbio.3001972)
Supplement: Attachment — Submitted filename: ReviewerReport_91222.pdf [file pbio.3001972.s022.pdf]

Dear Editor and Reviewers,

We would like to thank you for your careful review and insights. Please find our answers to all reviewer comments with details below.

1) We try to avoid punctuation in titles; please could you N-terminally truncate yours to "Phylogenomic analysis of Wolbachia genomes from the Darwin Tree of Life biodiversity genomics project"?

We have shortened the title following your suggestion.

2) Given the concerns raised by reviewer #3 about the limited novel biological insights (these were shared by the staff editors and Academic Editor before review), and in recognition that your study is nonetheless potentially of significant importance to our readership, please change the article type to "Methods and Resources" when you re-submit. No re-formatting is required.

Dear editor, we are happy to submit the manuscript as a "Methods and Resources" paper, however during re-submission we couldn't find the input field to change the article type.

3) We note that reviewer #2 suggests some additional analyses that would certainly add value and interest to your study (e.g. wmk and toxin genes); we leave it to you to decide whether to include these.

See comment reviewer #2.

4) Please provide a blurb, according to the instructions in the submission form. → Write a short, appealing statement summarizing your research, about 20-30 words or 1-2 sentences long

> ***TEXT ADDED***
> *Wolbachia* are common bacterial endosymbionts that manipulate reproductive biology in their hosts. Emmelien Vancaester and Mark Blaxter have assembled the genomes of 110 *Wolbachia* coincidentally sequenced with their hosts and find a rich diversity of host manipulation loci.

5) Please address my Data Policy requests below; specifically, we need you to supply the numerical values underlying Figs 1ACD, 2ABC, 3AB, 4AB, 5B, S1, S2ABC, S3, S4, S5, S6, S7, either as a supplementary data file or as a permanent DOI'd deposition. Please cite the location of the data clearly in all relevant main and supplementary Figure legends, e.g. "The data underlying this Figure can be found in S1 Data".

A supplemental dataset file has been created and for all relevant figures and this sentence has been added. Moreover, in the meantime all genome assemblies have been submitted and these identifiers are now available in table S2.

## Reviewer 1

1) Line 73: the authors should also refer to the Pascar & Chandler 2018 study (PMID: 30202647) who generated many Wolbachia draft genomes using a similar approach.

Thank you for pointing us to this study, this reference is now added to the manuscript.

2) Line 85-86: Does that mean that somatic tissues were selected for sequencing and ovaries generally discarded? Maybe specify. If that is the case, it would be useful to make it clear that some Wolbachia infections in this collection of insects may have been missed since Wolbachia infections can vary greatly in their tissue distribution, some being restricted to germline tissues (e.g. see Strunov et al 2022, PMID: 35357208).

To clarify which specific tissue was used for DNA extraction for PacBio sequencing, these data have been added to table S1. The figure below summarizes for each tissue the proportion that were positive for *Wolbachia* infection. No bias in the infection level for a tissue type was observed in our sampling set.

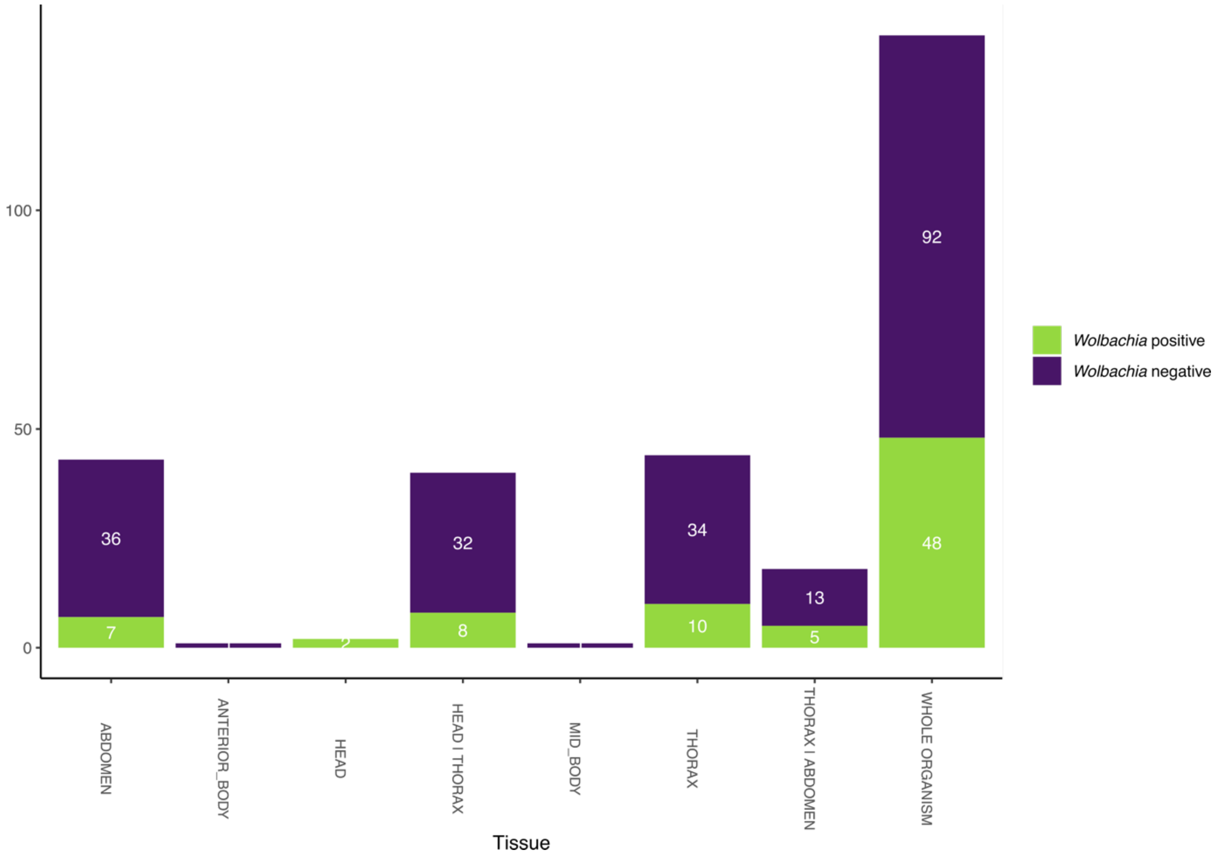


***DELETED***
The DToL project sequences genomes from individual, wild-caught specimens of target species, and thus will also generate data for the cobiome present in each specimen at the time of sampling. Where possible, DToL processing usually avoids body parts or tissues that are expected to have a high relative mass of cobionts. In smaller-bodied species, where the whole organism is extracted, and in cases where *Wolbachia* disseminates widely within an organism it is inevitable that cobiont genomes will be sequenced alongside the host genome.

***REPLACED WIITH***
The DToL project sequences genomes from individual, wild-caught specimens of target species, and thus will also generate data for the cobiome present in each specimen at the time of sampling. For many smaller-bodied insects the whole organism is extracted. Where *Wolbachia* disseminates widely within an organism it is inevitable that cobiont genomes will be sequenced alongside the host genome.

And included the following sentence in the results, referring to the figure above as an additional supplemental figure.

***TEXT ADDED***
Although maternal inheritance requires that *Wolbachia* are predominantly localised in the germline, tropism to somatic cell types has been shown to be highly regulated during host development (25,26). We did not observe a bias in infection level by analysed tissue type (Figure S1), or by gender, with an equal prevalence of infection in samples identified as female (39/138, 28%) and male (45/153, 29%) (Figure 1B)

3) Line 107-109: this is also in line with the more recent Weinert et al. 2015 study which accounted for sampling bias (PMID: 25904667).

I have included this additional reference on estimated Wolbachia arthropod incidence and slightly restructured this paragraph (see 4).

4) Lines 114-120: it might be useful to clearly define the difference between prevalence (proportion of infected individuals in a population/species) and incidence (proportion of host species where the symbiont is present in a given host clade).

These definitions have now been added to the text to help readability.

***DELETED***
Wolbachia SSU sequences were detected in 111 (30%) of the species. This degree of infection is similar to previous estimates (ranging from 22%(19,20) to 40%(11) of all arthropods). Wolbachia prevalence and infection intensity varies between species and between populations within a species(22,23). As only one individual was analysed for each taxon screened, the true level of infection within the insect biota surveyed by DToL is likely much higher.

***REPLACED WITH***
*Wolbachia* SSU sequences were detected in 111 (30%) of the species. This level of infection is not reflective of total incidence, the proportion of host species susceptible to infection, as only one individual was analysed for each taxon screened. *Wolbachia* prevalence, the proportion of infected individuals in a population, and infection intensity vary between species and between populations within a species (24,25). Therefore, the true incidence of infection within the insect biota surveyed by DToL is likely much higher. However, the measured incidence of infection is similar to previous survey-based estimates (22-23% (20,21)) but, as expected, is lower than estimates deploying mathematical models to account for sampling bias (40-50% (11,22)).

5) Line 146-148: there is evidence in the literature that Wolbachia titer/abundance within hosts can be controlled both by the host and the bacterial genomes. However, for most associations, the genetic determinants of Wolbachia proliferation have not been characterized. Therefore the statement "Most infected hosts tightly control Wolbachia proliferation" is inaccurate/not supported since looking at Wolbachia titers alone does not tell us whether it is controlled by the host, the symbiont or both.

Thank you for pointing this out, this has now been rephrased to avoid giving an active role to the host.

***DELETED***
Most infected hosts tightly control *Wolbachia* proliferation and have a relative abundance below ten *Wolbachia* genomes per host nuclear genome.

***REPLACED WITH***

*Wolbachia* proliferation seems to be tightly controlled and a relative abundance below ten *Wolbachia* genomes per host nuclear genome was observed in most infected hosts.

6) Line 149: 48 Wolbachia per host "genome".

This has been adapted.

7) Line 259-262: I suspect that a lot of these "novel genes" might be pieces of pseudogenes. The fact that they were found to be much shorter on average and mostly annotated as transposon/mobile elements is not surprising since transposase and reverse transcriptase genes are abundant in Wolbachia genomes and are often highly degraded. On top of this, these mobile elements often insert themselves within and disrupt other genes. As I understand, the genome annotations were not manually curated to reannotate pseudogenes and I wonder how much of these "novel genes" are simply degraded/split/truncated copies of genes that are present in full length in other genomes, but that OrthoFinder failed to place in the correct orthogroup, instead clustering them into a one/two member orthogroup. Could the authors elaborate on this and mention in the manuscript whether they think this is a limitation for defining what a novel gene is? I guess this is to be kept in mind when estimating the size of the core/pan-genomes and looking at variation between Wolbachia supergroups (some supergroups could have more degraded genomes for example which could artificially inflate the number of orthogroups detected).

We agree that a fraction of the novel genes might be the result of pseudogenisation (this is why we reported the average size reduction) and therefore the link with the mobile elements isn't unexpected. However, we do show that innovation with biological implications can be found in this set as exemplified by the novel metabolic genes in wCfeT. We have expanded this paragraph to clarify this. The core-and pangenome size were calculated excluding these novel genes.

***DELETED***
This suggests that much of the novelty arose through mobile elements other than WO phage.

***REPLACED WITH***
This suggests that much of the novelty is associated with mobile elements other than WO phage, but we note that the expansion in gene number may be due to mobile element-driven pseudogenisation.

8) Lines 314-315: can the authors explain their rationale for using the 80% threshold to define a prophage region as complete and what they mean by "complete"? If a prophage region carries >80% of genes of each phage module but is lacking an essential structural phage gene preventing it to produce phage particles, should it be called complete? Also, in line 476, defining a phage copy as "active" would mean that there is some evidence the phage is replicating and/or producing phage particles. I was wondering if the authors have any indication on which prophage region might be active, based on variation in sequencing depth. If not, I would probably avoid calling them active.

We agree that it is inaccurate to call these regions complete, we have changed this in the manuscript to putative complete. We allowed the absence of 1 gene per module to avoid gene annotation and gene clustering errors resulting in a decrease in number of "complete" WO regions. We have rephrased the corresponding part of the sentence in the abstract and discussion.

***DELETED***

Prophage regions were deemed complete when all four modules were observed with at least 80% of genes of each module present. An abundance of putative intact and pseudogenised WO phage were identified.

***REPLACED WITH***
Prophage regions were deemed putatively complete when all four modules were observed with at least 80% of genes of each module present. An abundance of putative intact and pseudogenised WO phage were identified.

***DELETED***
Moreover, we observed that genome size in *Wolbachia* is correlated with the abundance of active and pseudogenised copies of bacteriophage WO.

***REPLACED WITH***
Moreover, we observed that genome size in *Wolbachia* is correlated with the abundance of copies of bacteriophage WO.

9) Lines 345-351: from the method section, it seems that the authors used a coverage threshold for the detection of cif genes that should miss typical type V cif genes. If I understand correctly, representative cif genes from Type I (cidA/B) and Type IV (cinA/B) were used as queries and only hits that had 80-120% coverage were retained. However, type V cifB genes are typically much longer than type I-IV (4-5x longer) due to the presence of additional domains such as ankyrin repeat and a C-terminal latrotoxin domain (some type V cifB genes are shorter due to premature stop codons indicating pseudogenization or streamlining processes, however, the truncated ankyrin/latrotoxin domains are often found downstream of the disrupted genes). Therefore, my guess is that more full-length type V cif genes are present in this set of new genomes than reported in line 350 (50 type V homologues). For that reason, I also wonder how many of the latrotoxin domain-containing proteins reported in line 352 are in fact full copy or the 3'-end of truncated type V cifB proteins. I would suggest that the authors use representative type V cif genes in addition to type I-IV as queries or instead mention that they might be missing a lot of type V genes in their analysis. Another solution would be to remove the 120% maximum coverage threshold to include the longer cifB genes.

Thank you for sharing this concern. When I looked at the analysis we performed, we realised we had already carried out the analysis with representatives of each cif type, including two cifV genes, exactly for the reason you list, but had not updated the methods. As the CifB Type V are quite variable in their domain composition and therefore also length, we repeated the analyses, now also including the protein sequences for Diachasma_alloeum_pair1, Diploeciton_nevermanni_pair5, wBor_pair2, wStri_pair1, wStri_pair2 and wTri-2_pair1. This increased the number of cif type V pairs from 50 to 90. The numbers are updated in the result section.

***DELETED***
Two hundred and sixty one full-length and likely functional Cif pairs were detected in 133 of the 181 (73%) supergroup A and B genomes. One Cif pair was detected in most genomes, but many had several, with seven copies in the Wolbachia strain infecting the holly tortrix moth (Rhopobota naevana). Most of the gene pairs (93) contained a deubiquitinase domain (type I, Cid), while the other four types occurred in roughly equal proportions (II: 40, III: 43, IV:35 and V:50). Many pairs (177/261; 69%) were located in the predicted EAM of the prophage.

***REPLACED WITH***
Three hundred and five full-length and likely functional Cif pairs were detected in 140 of the 181 (77%) supergroup A and B genomes. One Cif pair was detected in most

genomes, but many had several, with seven copies in the *Wolbachia* strain infecting the holly tortrix moth (*Rhopobota naevana*). Most of the gene pairs contained a deubiquitinase domain (type I, Cid) (87) or belonged to type V (90), while the other three types occurred in roughly equal proportions (II: 39, III: 44, IV:34). Many pairs (213/305; 70%) were located in the predicted EAM of the prophage.

***TEXT ADDED***
***The following was modified in the Methods section "Gene content analysis"***
CidA: WP_010962721.1, WP_182158704.1, WP_012673228.1, WP_006014162.1, CAQ54402.1, NZ_MUIX01000001.1_1324, OAM06111.1; CifB: WP_010962722.1, WP_182158703.1, WP_012673227.1, WP_006014164.1, CAQ54403.1, NZ_MUIX01000001.1_1323, OAM06112.1. Moreover, additional CifB type V genes were added as reference genes (Diachasma_alloeum_pair1, Diploeciton_nevermanni_pair5, wBor_pair2, wStri_pair1, wStri_pair2 and wTri-2_pair1).

10) Lines 397-399: the balance also depends on loss of infections through time (not only gains).

Thank you for this comment, the text has been adapted as follows:

***DELETED***
The distribution of *Wolbachia* in insect hosts is a function of the balance between co-speciation (vertical transmission of *Wolbachia* among daughters of the host species) and horizontal transmission where strains move between species.

***REPLACED WITH***
The distribution of *Wolbachia* in insect hosts is a function of the balance between retention through co-speciation (vertical transmission of *Wolbachia* to daughters of the host species), acquisition through horizontal transmission (where strains move between species) and loss events.

11) Table S3: The reference genome accession GCF_001931755.2 was isolated from the Springtail *Folsomia candida* (Collembola), not from a coleopteran host as indicated in Table S3.

Thank you for your careful reading through the supplemental material, this mistake has been fixed.

# Reviewer 2

I don't have many comments or suggestions to strengthen the manuscript. Although the paper is very well-written and easy to read, I didn't find the discussion added very much new that wasn't already mentioned in the results or introduction. I would have also been interested to see more information about Wolbachia toxin evolution diversity, including some more phylogenies. The authors report the presence of spaid-like toxins, and it would be useful to get some more information about these. The spaid toxin was recently found to cause male-killing in Spiroplasma bacteria, so it would be interesting to understand what related genes are doing in Wolbachia (and how related they actually are). Finally, it would be interesting to learn more about the distribution and diversity of wmk genes, as these have been recently implicated in male-killing by Wolbachia. A recent study in biorxiv by Arai et al. found an interesting connection between male-killing and wmk copy number, and the high quality long-read sequence data presented in the current study has great potential to illuminate on this.

We thank the reviewer for this comment. We have now performed and included a phylogenetic analysis of the CifA and CifB genes, as well as TcA, TcB-C, ParD and ParE toxins (see figures S9-S15). The spaid-like toxins were identified using WP_010962723.1 described in Massey et al. (PMID 34253453) as a reference, however we acknowledge more in depth analysis is required to detect the correct orthologs in the dataset. Therefore, the spaid-like genes are now no longer discussed in the text. Similarly, ortholog delineation of wmk genes which retain the male killing characteristic proved challenging as we have no phenotypic information for each infection and we believe this analysis would merit its own study.

# Reviewer 3

1) Line 183-192. This is unsurprising given the literature on this topic. This data, like other datasets, shows that Wolbachia and host trees show many incongruences. However, it is also clear they are not independent. It seems that at the least this should be noted as a counterpoint to the observation of incongruences (eg a Mantel test comparing genetic distances of hosts and symbionts). In the future, this may be a nice resource to understand what predicts which species Wolbachia jumps between.

We agree that this is indeed an important pattern to point out. While this was already addressed in the discussion, we have now added an additional sentence to this paragraph:

> ***TEXT ADDED***
> Although horizontal transmission seems to have been a dominant pattern in the evolutionary history of *Wolbachia*, the propensity of Lepidoptera to be infected by *Wolbachia* type B underlines the importance of distribution by co-speciation.

2) Figure 2C. This plot is most effective at showing synteny but it is used to demonstrate a recent host shift of Wolbachia between insect orders. There needs to be a clearer explanation of why this is the best way to show this - a tree seems more straightforward. I guess the answer is likely in Figure 3C, but this comes after.

We want to show with Figure 2C that this is a recent host switching event between different insect orders, as almost no rearrangements were observed among these *Wolbachia* strains. We agree that this can also be shown in the phylogenetic tree in 2B, and the branch containing these five species is now highlighted in a red box.

3) Figure 3A. Is ANI just coding sequence? State In legend. The description of this in the text seems a bit odd as the correlation of sequence and structural divergence seems somewhat inevitable - maybe commenting on the degree of rearrangements might be more useful? It looks like synteny is pretty low except between very closely related strains.

No, the average nucleotide identity (ANI) was measured on a genome-wide level. This has now been clarified in the legend. We agree that the correlation between sequence and structural divergence is indeed expected and have included an additional sentence at the end of this paragraph:

> ***TEXT ADDED***
> This broad range of nucleotide diversity, even within a supergroup, is indicative of the low level of conserved synteny within supergroups and the level of rearrangements occurring.

4) Line 218-231. The definition of GC skew/skewI needs a clearer explanation. Explain why it is plotted against GC content. The interpretation of this statistic is a bit unclear. It is stated that

groups A and B have low skew. Is this just relative to other supergroups, or bacteria in general? If the latter, does this translate directly into a measure of the rate of genome rearrangement.

We have clarified the definition of GC skew:

> ***TEXT ADDED***
> GC skew accumulates in stable bacterial genomes through differential mutation pressures on leading versus lagging strands. → Stable bacterial genomes accumulate more guanines than cytosines on the strand in the direction of replication. This phenomenon, GC skew, arises due to differential mutation pressures on leading versus lagging strands.

Groups A and B have a low level of GC skew relative to some of the other supergroups, but also to most bacterial groups. Calculation of SkewI of all complete bacterial genomes in NCBI's RefSeq library by Lu et al. (doi.org/10.1371/journal.pcbi.1008439) revealed that most genomes have strong GC skew patterns, with relatively few having SkewI values less than 0.5 (see their S2 Fig). For example, genomes from the genera of *Bacillus*, *Escherichia*, and *Salmonella* have consistently high SkewI values, with a mean close to 0.9. GC skew is thus indeed an indirect measure of the degree of rearrangement, as we already discussed in the last sentence of this paragraph:

> ***TEXT ADDED***
> A high degree of GC skew was previously reported in supergroup C *Wolbachia* strains infecting filarial nematodes (35) and these genomes also have low rearrangement levels and high gene-level synteny. In supergroups A and B the low level of skew is associated with high levels of chromosomal rearrangement (Figure 3A).

5) Line 352-362. The distribution of different toxin genes is interesting but a bit hard to follow. A supplementary table or figure would make it more digestible.

A supplementary table has been created which shows the number of detected toxin genes per supergroup as well as those positioned in prophage regions.

6) Line 406 'less likely' needs some justification. Line 412 seems to suggest ecological effects matter, and if there is preferential switching between hosts with shared ecology which will generate phylogenetic clustering. It is also unclear how 'host genetics' and 'Wolbachia' genetics differ in this list - presumably you mean the interaction of the two.

We have slightly rephrased these sentences, see comment below.

7) Line 423-426. This seems like a very important pattern, but the text here does not seem to flow clearly though.

We have inserted this statement earlier in the paragraph and combined these two to enhance readability.

> ***DELETED***
> The distribution of Wolbachia in insect hosts is a function of the balance between co-speciation (vertical transmission of Wolbachia among daughters of the host species) and horizontal transmission where strains move between species. Transmission among insect hosts was the dominant pattern underpinning Wolbachia distribution, but we identified two features of the distribution, one local and one general, that are of note. Lepidoptera were more likely to be infected with supergroup B Wolbachia than A, and Hymenoptera, Diptera and Coleoptera were more likely to be infected with supergroup A strains. Multi locus sequence typing (MLST) has previously shown that

supergroup B is the most common Wolbachia type in Lepidoptera(19,27–29). This suggests some non-exclusive specialisation of Wolbachia on their hosts, which may be driven by Wolbachia genetics, host genetics or (less likely) a distinct set of ecological transmission routes in each insect group. Many of our genomes derived from insects were collected at one site, the Wytham Woods Genomic Observatory (Figure S1) but this subset was no more closely related than other genomes from widely separated sites (Figure S5). It is likely that the mobility of hosts, including through seasonal migration, means that sampling from one geographical site is a valid approximation of more global sampling.

Close ecological association between host species may promote sharing of Wolbachia isolates and localised genetic exchange, for example within predator-prey systems. The close similarity of Wolbachia genomes from Andrena solitary bees and their Nomada cuckoo bee kleptoparasites, and the shared occurrence of the biotin synthesis operon (Figure 4C) may be a case of transmission within an ecological network. The presence of the biotin operon in Wolbachia of insects that largely or solely feed on low-protein plant fluids (nectar or phloem) suggests that the Wolbachia may be offering nutritional support to their hosts(48), and thus that this cluster of genomes may have been positively selected for their mutualist tendencies. Other genes whose distribution among isolates is driven by horizontal gene transfer, including by mobile elements such as phage, might be expected to have a distribution that is not explained by overall genome relatedness, and might reflect ecological association. We note that previous work has suggested that horizontal transmission rather than cospeciation may also explain closely related Wolbachia in closely related taxa. For example, genomic divergence between closely related Wolbachia in sister Drosophila species was too low to be the product of independent evolution since the last common ancestor of the flies(49,50).

***REPLACED WITH***

The distribution of *Wolbachia* in insect hosts is a function of the balance between retention through co-speciation (vertical transmission of *Wolbachia* to daughters of the host species), acquisition through horizontal transmission (where strains move between host species) and events of loss. Transmission among insect hosts was the dominant pattern underpinning *Wolbachia* distribution. We note that previous work has suggested that horizontal transmission rather than cospeciation may even explain the presence of closely related *Wolbachia* infecting closely related taxa. For example, genomic divergence between closely related *Wolbachia* in sister *Drosophila* species was too low to be the product of independent evolution since the last common ancestor of the flies(52,53). However, we identified two features of the distribution, one local and one general, that are of note. Lepidoptera were more likely to be infected with supergroup B *Wolbachia* than A, and Hymenoptera, Diptera and Coleoptera were more likely to be infected with supergroup A strains. Multi locus sequence typing (MLST) has previously shown that supergroup B is the most common *Wolbachia* type in Lepidoptera(22,31–33). This suggests some non-exclusive specialisation of *Wolbachia* on their hosts, which may be driven by the interaction of *Wolbachia* and host genetics and/or a distinct set of ecological transmission routes in each insect group. Many of our genomes derived from insects were collected at one site, the Wytham Woods Genomic Observatory (Figure S2) but this subset was no more closely related than other genomes from widely separated sites (Figure S5). It is likely that the mobility of hosts, including through seasonal migration, means that sampling from one geographical site is a valid approximation of more global sampling. Close ecological association between host species may promote sharing of *Wolbachia* isolates and localised genetic exchange, for example within predator-prey systems. The close similarity of *Wolbachia* genomes from *Andrena* solitary bees and their *Nomada* cuckoo bee kleptoparasites (Figure 4C, inset), and the shared occurrence of the biotin synthesis operon (Figure 4C) may be a case of transmission within an ecological

network. The presence of the biotin operon in *Wolbachia* of insects that largely or solely feed on low-protein plant fluids (nectar or phloem) suggests that *Wolbachia* may be offering nutritional support to their hosts(54), and thus that this cluster of genomes may have been positively selected for their mutualist tendencies.

8) Line 427. I guess you mean 'female hosts'. The rest of this paragraph could do with a few citations of similar work.

Thank you for pointing us to this mistake. We have added references which discuss the conflict among the different CI types and the different proposed models of rescue mechanisms and role of the host.

9) Are there any plasmids?

While we sometimes did observe additional small contigs during the assembly of the Wolbachia-classified reads, these were mostly linear. We didn't analyse these in depth, so these could be the result of

1) heteroplasmic variation or overlapping regions when multiple strains are present in a sample causing assembly errors
2) remnants of NUWTs (nuclear insertion of Wolbachia DNA in the host genome).
3) WO viroid particles or other DNA viruses present in the sample

10) Fig S5. What is the statistical test?

Thank you for pointing this out, we have now adapted the legend of Figure S3 and Figure S6. The statistical test used to compare distributions was the Wilcoxon rank sum test.
